# *Arabidopsis thaliana* BOLA proteins, new actors in plant growth and iron homeostasis

**DOI:** 10.1111/tpj.71196

**Published:** 2026-11-04

**Authors:** Maël Taupin‐Broggini, Anaïs Rodriguez‐Euzet, Brigitte Touraine, Nathalie Berger, Julie Bals, Daniel Marino, Christian Dubos, Florence Vignols

**Affiliations:** ^1^ IPSiM, Univ Montpellier, CNRS, INRAE, Institut Agro Montpellier France; ^2^ Department of Plant Biology and Ecology, Basque Center for Integrative Environmental and Human Health (EHU‐iBio) University of the Basque Country Bilbao Spain

**Keywords:** iron homeostasis, BOLA proteins, plant growth, iron–sulfur clusters, organelles, *Arabidopsis thaliana*

## Abstract

The universal iron–sulfur (Fe‐S) clusters embedded in proteins support numerous fundamental biological processes. However, many proteins involved in Fe‐S assembly machineries remain poorly characterized in plants. Here, we revealed that *BOLA1* and *BOLA4,* two members of the BOLA family previously described as Fe‐S assembly factors in various organisms, are critical growth regulators that play a role in Fe homeostasis in *Arabidopsis thaliana*. Expression analyses showed that *BOLA1* and *BOLA4* are ubiquitously expressed across most Arabidopsis tissues. Using protoplast transfection assays, we also demonstrated that both BOLA‐GFP fusion proteins localize to chloroplasts and mitochondria. Mutant analyses revealed that while single *bola1* or *bola4* knock‐out mutants are phenotypically indistinguishable from the wild type, double *bola1 bola4* mutants display severe growth defects, including early growth arrest of the primary root. Notably, this phenotype was rescued by grafting a wild‐type root system onto the *bola1 bola4* aerial part. Fe quantification, Perls staining, and RT‐qPCR analyses further demonstrated that *bola1 bola4* double mutants exhibit altered Fe distribution and Fe‐homeostasis‐related gene expression. Overall, our findings show that *BOLA1* and *BOLA4* act redundantly to maintain primary root growth and rosette development and are essential players in Fe signaling and partitioning in Arabidopsis.

## Significance Statement

This study investigates two members of the BOLA family which have been implicated in [Fe‐S] cluster delivery in several organisms, although their biological importance in plants remained unclear. Herein, we show that BOLA1 and BOLA4 act redundantly in both plastids and mitochondria to sustain root growth and maintain iron partitioning in *Arabidopsis thaliana*.

## INTRODUCTION

BOLA proteins are small ubiquitous proteins found across a wide range of living organisms. First discovered in *Escherichia coli*, they were initially described as transcription factors essential for the establishment of the stationary growth phase in stress conditions (da Silva et al., 2023). Subsequent studies conducted in various organisms revealed that BOLA proteins were also involved in Fe‐Sulfur ([Fe‐S]) assembly pathways (reviewed by Przybyla‐Toscano et al., 2018; Rey et al., 2019).

In eukaryotes, the BOLA family is composed of four members with distinct locations within cells. BOLA1 is also present in all organisms except archaea and cyanobacteria (Couturier et al., 2014), and resides in the mitochondria (Cameron et al., 2011; Melber et al., 2016; Uzarska et al., 2016), except in green phyla where it was initially described as a plastidial protein (Couturier et al., 2014; Qin et al., 2015). BOLA2 is the unique cytosolic isoform and is found in all phyla analyzed to date (Willems et al., 2013). Like BOLA1, BOLA3 is also detected in the mitochondria, but is restricted to non‐photosynthetic eukaryotes (Melber et al., 2016). Conversely, BOLA4 is found in green phyla with a dual location in the plastid and in the mitochondria (Couturier et al., 2014).

BOLA1 and BOLA3 in yeast and human are both implicated in mitochondrial [Fe‐S] protein maturation. *Saccharomyces cerevisiae* yeast *Δbol1bol3* mitochondrial loss‐of‐function mutants, but not *Δbol1* or *Δbol3* single mutants, exhibit a sharp decrease in [Fe‐S] protein content, protein lipoylation, and decreased respiratory chain activity, highlighting functional redundancy between these two BOLA proteins (Melber et al., 2016; Uzarska et al., 2016). Depletion of human BOLA1 and BOLA3 was also found to induce additional mitochondrial defects, including disruption of the cell redox state (Cameron et al., 2011; Maio & Rouault, 2020). The cytosolic BOLA2 proteins are involved in the maintenance of Fe homeostasis in various organisms (reviewed in Li & Outten, 2019; Rey et al., 2019; Talib & Outten, 2021). For instance, in mammals, the lack of BOLA2 was correlated with mild anemia in human and mice with the disruption of Fe homeostasis (Giannuzzi et al., 2019). Although it is unclear how BOLA2 can act in Fe homeostasis in mammals, it is hypothesized that BOLA2 can control the activity of IRP1, a post‐transcriptional regulator of Fe homeostasis (Xia et al., 2015). In Fe deficiency condition, IRP1 binds regions of mRNAs regions that encode for proteins involved in Fe assimilation and storage, which are called Fe Responsive Elements (IREs). According to the position of the IREs in mRNA sequences, IRP1 can stabilize the mRNA or block its translation. In Fe replete condition, IRP1 receives a [4Fe‐4S] cluster to acquire aconitase activity and interrupt its binding to IREs. BOLA2 would therefore acts as a Fe deficiency response repressor.

In plants, studies on BOLA proteins are scarce, with most studies focused on *Arabidopsis thaliana*. The family is composed of three canonical BOLA proteins (BOLA1, BOLA2, and BOLA4) and SULFUR E1 (SUFE1), a longer isoform exhibiting a N‐terminal SUFE domain fused to a BOLA1‐like region (Couturier et al., 2014). The physiological importance of SUFE1 and BOLA2 has been studied in Arabidopsis. SUFE1 was shown to enhance the cysteine desulfurase activity of NIFS‐LIKE CYSTEINE DESULFURASE 2 (NFS2), a crucial chloroplast component acting in the first step of the plastidial Fe‐S assembly machinery (Van Hoewyk et al., 2007). A potential role for cytosolic BOLA2 in resistance to Fe overload and oxidative stress has been suggested (Qin et al., 2015), but the mechanisms by which BOLA2 functions remain unclear. The importance and roles of BOLA1 and BOLA4 in plants have yet to be characterized. A preliminary step toward understanding their function was the discovery that both proteins can rescue the yeast *Δbol1bol3* mutant, indicating the conservation of at least the mitochondrial function of yeast BOLA in Arabidopsis (Bandyopadhyay et al., 2008; Uzarska et al., 2018). This finding also questions the current assumption of BOLA1 exclusive plastidial localization.

In the present study, we employed physiological, molecular, and genetic approaches to assess the importance and potential roles of BOLA1 and BOLA4 proteins in Arabidopsis. Collectively, the data presented highlight the crucial role of BOLA1 and BOLA4 proteins in plastids and mitochondria for proper plant growth and for Fe partitioning.

## RESULTS

### Expression analysis of *BOLA1* and *BOLA4*

The accumulation of *BOLA1* and *BOLA4* transcripts was first examined across various tissues from wild‐type (WT) Arabidopsis plants by quantitative reverse transcription‐polymerase chain reaction (RT‐qPCR) (Figure 1A). Both gene transcripts were detected in all tissues tested, with *BOLA4* consistently showing higher expression levels than *BOLA1*, which aligns with findings from large‐scale expression studies (Figures S1 and S2). To gain further insight into *BOLA1* and *BOLA4* expressions throughout the plant's lifespan, we analyzed their expression in plants of different ages from 2 to 15 days after germination (Figure 1B). The expression of both *BOLA* genes was detected in all samples, with higher expression levels observed for *BOLA4* compared to *BOLA1*.

**Figure 1:**
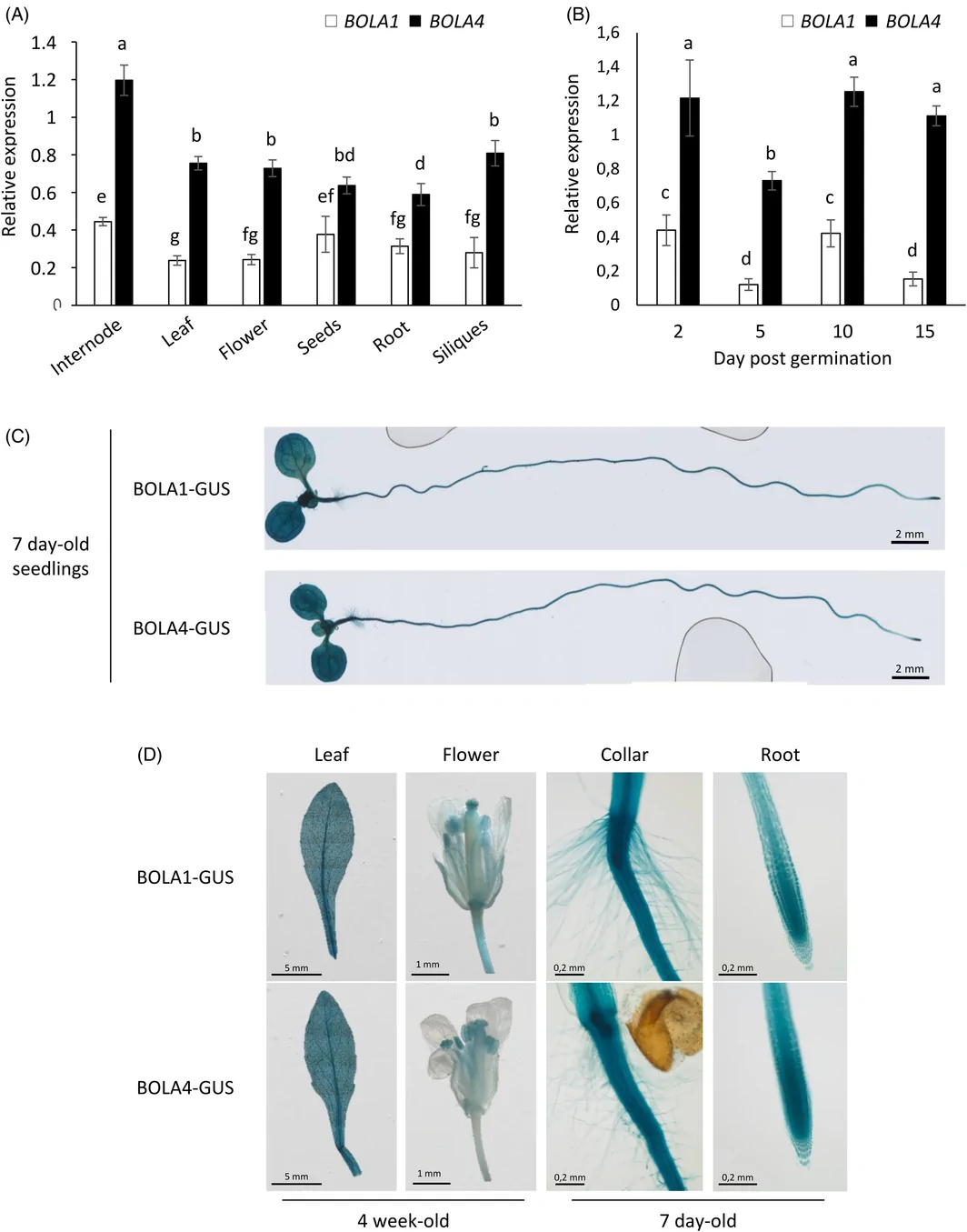
The *Arabidopsis thaliana BOLA1* and *BOLA4* genes are expressed throughout the whole‐plant body. *BOLA* transcript abundance was analyzed by RT‐qPCR in different tissues (A) and at different growth stages in wild‐type plants (B), using the *PP2AA3* gene as control. (A, B) Means within each condition with the same letter are not significantly different according to the Kruskal–Wallis test with post hoc analysis (Conover, 1999), *P* < 0.01, with Holm corrections. Error bars represent means ± SD. All analyses were done in 3 independent biological repeats, with each experiment done in triplicates. (C, D) GUS staining patterns of BOLA1‐GUS and BOLA4‐GUS fusion proteins expressed under their own promoter. Seedlings (C), collars, and roots (D) were photographed 7 days post germination on sterile ½MS media, while leaves and flower images were taken on four‐week‐old plants grown on soil. Images are representative of 3 independent lines for each fusion protein.

To further investigate *BOLA1* and *BOLA4* expression patterns, we analyzed transgenic plants expressing BOLA‐GUS fusion proteins driven by either the *proBOLA1:gBOLA1‐uidA* or *proBOLA4:gBOLA4‐uidA* transgenes. BOLA1‐GUS and BOLA4‐GUS proteins were detected in all the tissues examined, regardless of developmental stage or organ, with noticeably stronger GUS staining in secondary root initials at the collar region (Figure 1C,D). No differences were observed between the expression patterns of the two BOLA proteins.

### BOLA1 and BOLA4 display redundant functions

To explore the role of BOLA1 and BOLA4 in plants, we next analyzed Arabidopsis T‐DNA insertional *bola1* (i.e., *bola1‐3* and *bola1‐4*) and *bola4* (i.e., *bola4‐1*) single mutants (Figure S3A) in which the absence of *BOLA1* and *BOLA4* gene expression, respectively, was confirmed (Figures S4 and S5). We also created a second mutant allele for *BOLA4* by Crispr‐Cas9 genome editing (named *bola4‐2*), that carries a deletion of 5 nucleotides in the coding sequence leading to BOLA4 amino acid sequence frameshift, and premature stop codon (Figures S3B,C, S4 and S5), without impacting predicted off‐targets (Figure S6). Despite mutations, all the single *bola* mutants showed growth similar to that of the WT plants, with the exception of a slight reduction in root growth in the two *bola4* mutants (Figure S7).

Suspecting a functional redundancy between the two BOLA proteins, we constructed *bola1‐4 bola4‐1* and *bola1‐3 bola4‐2* double mutants by crossing single knock‐out mutants. In contrast to single mutants, both double mutants displayed severe growth defects, including a strong reduction in shoot development and root growth, resulting in dwarf plants (Figure S8A). We notably observed the early emergence of lateral roots visible at 6 days post‐germination, emerging under the collar and leading to a fascicular root system, with rapid primary root arrest (Figures S8B and S9). These analyses, combined with measurements of root growth and branching over time, highlighted that the double *bola* mutation strongly impaired the overall root system development.

To deeper characterize *bola1 bola4* double mutants, we selected *bola1‐4 bola4‐1* and its related *bola1‐4* and *bola4‐1* single mutants for further analyses. We found that the striking differences in growth and weight between the single *bola* mutants and the double *bola1‐4 bola4‐1* mutant persisted over time (Figure 2A–C). We also observed that the absence of *BOLA1* expression did not induce *BOLA4* expression in the *bola1‐4 bola4‐1* mutant, nor did the absence of *BOLA4* induce *BOLA1* expression (Figure 2D). To confirm that the observed phenotypes were due to the combined absence of *BOLA1* and *BOLA4* expression, the *bola1‐4 bola4‐1* double mutant was complemented with either the *proBOLA1:gBOLA1* or the *proBOLA4:gBOLA4* constructs. In both cases, the expression of either *BOLA1* or *BOLA4* successfully restored *bola1‐4 bola4‐1* double mutant growth similar to that of the WT (Figure 2E), confirming that BOLA1 and BOLA4 have redundant roles.

**Figure 2:**
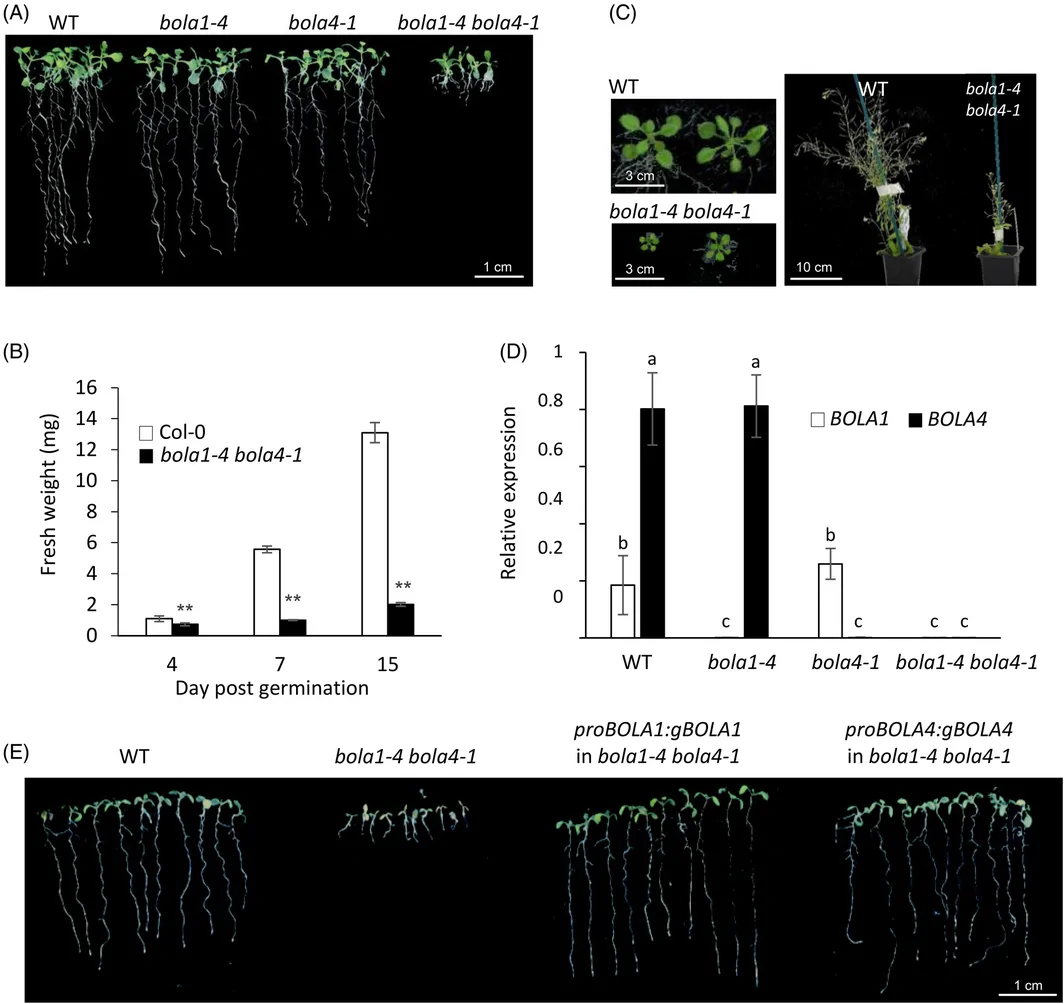
BOLA1 and BOLA4 are redundant proteins required for proper development of *Arabidopsis thaliana*. (A) Morphological comparison of 12‐day‐old wild type (WT), *bola1‐4*, *bola4‐1* and *bola1‐4 bola4‐1* mutants. (B) Fresh weight of the WT and *bola1‐4 bola4‐1* mutant lines at different growth stages. Means within each condition with the same letter are not significantly different according to one‐way ANOVA with post hoc Tukey test, *P* < 0.05. Bars represent means ± SD. (C) Comparison of the WT and *bola1‐4 bola4‐1* rosettes (Left panels) and mature plants (Right panel), 2 weeks and 3 months post germination, respectively. (D) Relative expression of *BOLA1* and *BOLA4* in 15‐day‐old WT and mutant lines shown in (A), using the *PP2AA3* gene as control. Means within each condition with the same letter are not significantly different according to Kruskal‐Wallis test with post hoc analysis (Conover, 1999), *P* < 0.01, with Holm corrections. Bars represent means ± SD. (E) Morphological comparisons between seven‐day‐old wild type, *bola1‐4 bola4‐1* mutant and the complemented transgenic mutant line with the endogenous version of *BOLA1* or *BOLA4* downstream their own promoter. (A, C, E) Plants were grown on ½ MS medium containing 1% sucrose, either vertically (A, E) or horizontally (C).

### Deciphering BOLA1 and BOLA4 subcellular localization *in planta*

Previous studies indicated that among plant BOLAs, only BOLA4 had a dual localization in plastid and mitochondria, whereas BOLA1 was confined to the plastid (Couturier et al., 2014). Based on our observations that BOLA1 and BOLA4 equally complement the double *bola1‐4 bola4‐1* mutant (Figure 2E) and the mitochondrial defect of the *bol1 bol3* yeast mutant (Uzarska et al., 2018), we decided to reassess the subcellular location of BOLA1. However, transfection of WT protoplasts with a construct expressing BOLA1‐GFP under its promoter (*proBOLA1:BOLA1‐GFP*) only confirmed the localization of BOLA1‐GFP in the plastid (Figure 3A and Figure S10A), while BOLA4‐GFP expressed under its own promoter could be detected in both the plastid and the mitochondria (Figure 3A).

**Figure 3:**
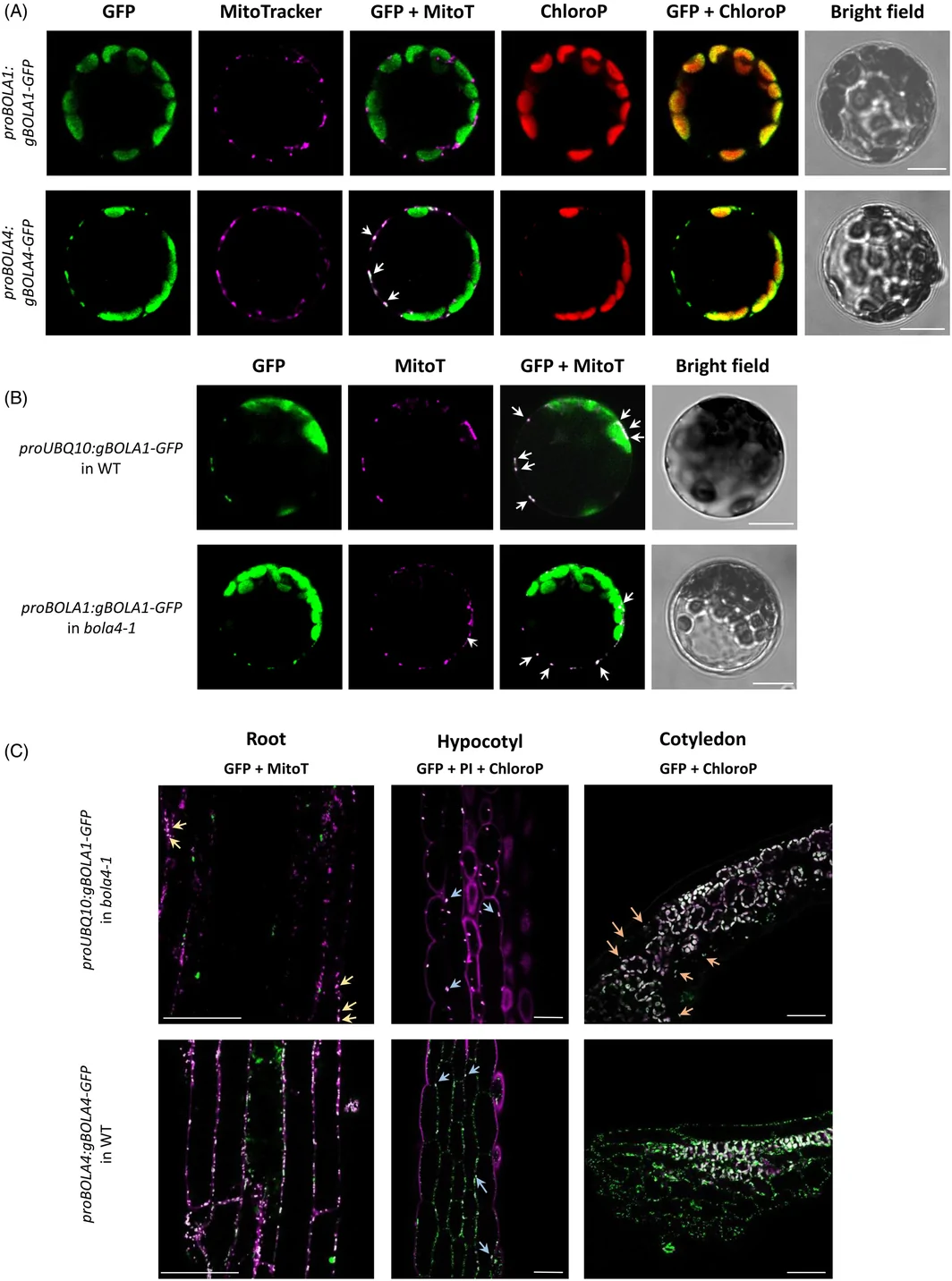
BOLA1, like BOLA4, localizes to plastids and mitochondria in Arabidopsis. (A) Subcellular localization of BOLA1‐GFP and BOLA4‐GFP fusion proteins expressed from genomic copies of their respective genes under the control of their native promoters (*proBOLA1:gBOLA1‐GFP* and *proBOLA4:gBOLA4‐GFP*) in wild type (WT) leaf protoplasts. (B) Localization of BOLA1‐GFP expressed either under the control of the *UBIQUITIN10* promoter (*proUBQ10:gBOLA1‐GFP*) in WT leaf protoplasts or under its native promoter (*proBOLA1:gBOLA1‐GFP*) in *bola4‐1* leaf protoplasts. (A, B) Arrows indicate mitochondria where BOLA1‐GFP or BOLA4‐GFP fluorescence overlaps with MitoTracker™ CMROS Orange (MitoT; pink). Chloroplasts (ChloroP; red) are identified by chlorophyll autofluorescence. Overlays of GFP and organelle marker fluorescence are shown in separate panels. Images were acquired by confocal microscopy 24 h after transfection and are shown without maximum Z‐axis projection (maximum projections are provided in Figure S11). All images are representative of five biological replicates, each comprising approximately 20 cells displaying identical protein localization. Scale bars = 10 μm. (C) Localization of BOLA1‐GFP and BOLA4‐GFP fusion proteins in 7‐day‐old *bola4‐1* and WT plant tissues, respectively. BOLA1‐GFP was expressed under the *UBQ10* promoter (upper panels), whereas BOLA4‐GFP was expressed under its native promoter (*proBOLA4*; lower panels). Panels show only merged images (white patterns in all tissues shown) where BOLA‐GFP fluorescence (green) colocalized with fluorescent markers for mitochondria (MitoT), cell walls (propidium iodide, PI), and chlorophyll (ChloroP). Extended views showing individual fluorescence channels are provided in Figure S11. Yellow and orange arrows indicate where BOLA1‐GFP colocalized with MitoT, whereas blue arrows highlight some of the plastids where both BOLA1‐GFP and BOLA4‐GFP localize. Images are representative of at least 10 plants from two independent transgenic lines per BOLA‐GFP analyzed. Scale bars = 50 μm.

BOLA1 has a much weaker expression than BOLA4 (Figure 1 and Figures S1 and S2). We thus reasoned that BOLA1 expressed under its native promoter may not be detectable in WT protoplast mitochondria because of accumulation levels remaining under the GFP detection threshold. To challenge this hypothesis, we over‐expressed BOLA1‐GFP under the strong *UBIQUITIN 10* (*UBQ10*) gene promoter in WT protoplasts. This test allowed us to detect BOLA1‐GFP in both the plastid and the mitochondria (Figure 3B and Figure S10B), supporting that BOLA1 can reside in certain conditions in this compartment. Since we observed functional redundancy between *BOLA1* and *BOLA4*, we sought to determine how BOLA1 behaves in the absence of BOLA4. To this end, we analyzed the localization patterns of the BOLA1‐GFP protein under the control of its own promoter in protoplasts prepared from the *bola4‐1* single mutant. In a *bola1‐4* background, a consistent fluorescent signal of BOLA1‐GFP similar to that obtained when expressed under the *UBQ10* promoter was observed in both organelles (Figure 3B and Figure S10B).

To validate the subcellular localization of BOLA1 and BOLA4 in plant tissues, we further analyzed seven‐day‐old transgenic plants expressing BOLA‐GFP fusion proteins driven by either the *proBOLA1:gBOLA1‐GFP* or *proBOLA4:gBOLA4‐GFP* transgenes (Figure 3C and Figure S11). In the WT background, the BOLA1‐GFP fluorescence signal was below the detection limit, consistent with the low endogenous expression of *BOLA1* (Figure S1). However, when expressed under the control of the *UBQ10* promoter in the *bola4‐1* background, BOLA1‐GFP fluorescence was readily detected in chloroplasts of cotyledons and hypocotyls (Figure 3C and Figure S11A,B). In addition, BOLA1‐GFP localized to a small number of mitochondria in epidermal cells of cotyledons (Figure 3C and Figure S11A) and roots (Figure 3C and Figure S11C,D). BOLA4‐GFP was robustly detected in mitochondria of epidermal cells in all WT tissues examined (Figure 3C and Figure S11C,D), as well as in multiple types of plastids, including chloroplasts in aerial tissues (Figure 3C and Figure S11A,B) and globular or stromule‐forming plastids in roots (Figure 3C and Figure S11C,D). These observations are consistent with the higher expression levels of *BOLA4* compared to *BOLA1* across most tissues analyzed (Figure 1 and Figure S1).

Collectively, these results support that BOLA1 and BOLA4 exhibit dual localization in plastids and mitochondria. BOLA4 appears to be the predominant mitochondrial isoform in root cells, while BOLA1 is only detectable in this organelle in the absence of BOLA4. This suggests that BOLA1 may function redundantly with BOLA4 in mitochondria under critical conditions.

### The *bola1 bola4* double mutant displays a constitutive Fe deficiency response

Plastids and mitochondria are among the cellular compartments with the highest Fe demand. Moreover, BOLA proteins in plants (Qin et al., 2015), yeast (Kumánovics et al., 2008; Li & Outten, 2012; Poor et al., 2014), and humans (Rouault & Maio, 2017) have been shown to be indirectly involved in Fe homeostasis. To determine whether BOLA1 and BOLA4 are also associated with this nutrient, we first examined their gene expression and found that both genes were induced in plants grown under excess‐Fe conditions (Figure 4A), without modification of the BOLA1 and BOLA4 distribution in plant tissues (Figure S12). We also analyzed the growth of the *bola1‐4 bola4‐1* double mutant under these conditions. As previously described (Gao et al., 2020), adding 250 μM Fe‐EDTA in the medium led to reduced growth in the WT plant, which was confirmed in our experiments (Figure 4B). The reduction of root growth was accompanied by a decrease in the WT fresh weight (Figure 4C). In contrast, high‐Fe concentrations weakly complemented the phenotypes of the *bola1‐4 bola4‐1* double mutant, including root and rosette growth and development (Figure 4B), as evidenced by a fresh weight increase (Figure 4C). This was accompanied by enhanced primary root growth (Figure 4D), in contrast to the WT's response under the same high‐Fe conditions. These observations indicate that Fe excess has slight beneficial effects on the *bola1‐4 bola4‐1* growth, although it *remained dwarf*.

**Figure 4:**
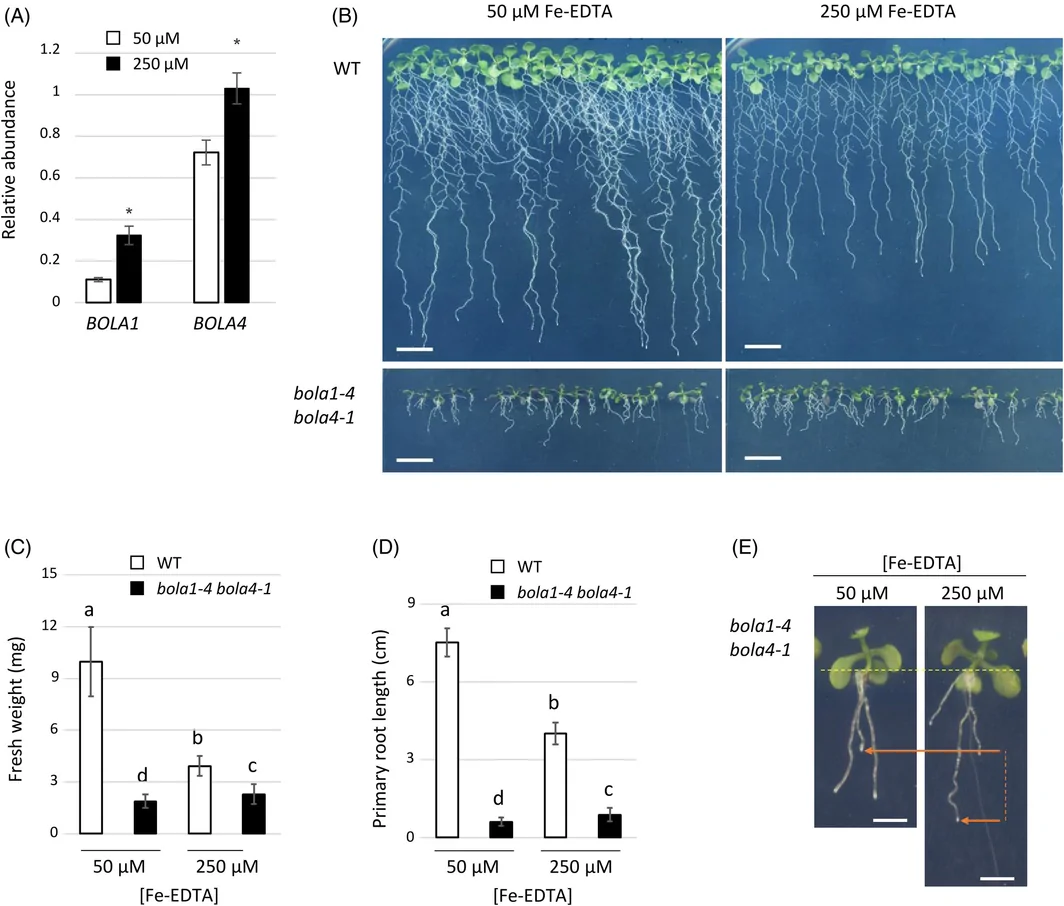
Excess iron slightly promotes the growth of *the bola1‐4 bola4‐1* mutant. (A) *BOLA* genes expression was analyzed by RT‐qPCR in 12‐day‐old wild type (WT) seedlings grown vertically on ½ MS medium containing 1% sucrose, under control (50 μM Fe‐EDTA) or high (250 μM Fe‐EDTA) iron conditions, and using the *PP2AA3* gene as control. Experiments shown illustrate one biological repeat out of 4, with each experiment performed in triplicates. (B) Effect of iron excess on 12‐day‐old WT and *bola1‐4 bola4‐1* plant growth. Scale bars = 1 cm. (C) Fresh weights measurement and (D) primary root length of plants grown as in (A). (A, C, D) Means within each condition with the same letter are not significantly different according to one‐way ANOVA with post hoc Tukey test, *P* < 0.01 (*n* > 30). Error bars show means ± SD. B) Picture of *bola1‐4 bola4‐1* plant growth as in (A). The arrows indicate the primary root. Scale bars = 2 mm.

To rule out the possibility that the phenotype observed in the mutant was due to the use of iron chelated with EDTA, we repeated the experiment using ethylenediamine di(o‐hydroxyphenylacetic) acid (EDDHA), a stable and effective Fe chelator commonly used for dicotyledonous plant cultures (Nadal et al., 2009). To this end, plants were grown on ½ MS medium supplemented with control (50 μM) or high (250 and 350 μM) Fe‐EDDHA concentrations (Figure S13A), and their growth was recorded. We observed the same trends as those obtained with Fe‐EDTA for both primary root length (Figure S13B,C) and fresh weight (Figure S13D), confirming that iron excess promotes *bola1‐4 bola4‐1* growth independently of the Fe chelator used.

To unravel *bola1‐4 bola4‐1* molecular responses to Fe excess, we next analyzed the expression of selected genes involved in Fe uptake, homeostasis regulation and storage by RT‐qPCR (Figure 5). Roots from seven‐day‐old WT and *bola1‐4 bola4‐1* double mutant were then grown under control (50 μM Fe) and high Fe (250 μM Fe). These analyses revealed a strong increase in the expression of *FRO2* (*FERRIC REDUCTASE OXIDASE 2*) and *IRT1* (*IRON‐REGULATED TRANSPORTER 1*), two genes involved in root Fe uptake and induced in response to Fe deficiency (Robinson et al., 1999; Vert et al., 2002), in the *bola1‐4 bola4‐1* double mutant under control condition compared to the WT (Figure 5A). Under Fe excess, their expression remained higher in *bola1‐4 bola4‐1* than in the WT, though to a lesser extent. Such increase of *IRT1* and *FRO2* gene expression was accompanied by a 23‐fold increase of FRO2 activity in the mutant root under Fe‐sufficient condition (Figure 5B), suggesting that the double *bola1‐4 bola4‐1* mutation stimulates the Fe uptake system at the root. Consistent with the increase of *IRT1* and *FRO2* gene expression, we also observed an increased expression of *FIT* (*FE‐DEFICIENCY INDUCED TRANSCRIPTION FACTOR 1*) and *bHLH39* (*BASIC HELIX‐TURN‐HELIX FACTOR 39*) (Figure 5C), two Fe deficiency response bHLH transcription factors that directly regulate the expression of *FRO2* and *IRT1* (Colangelo & Guerinot, 2004; Yuan et al., 2008).

**Figure 5:**
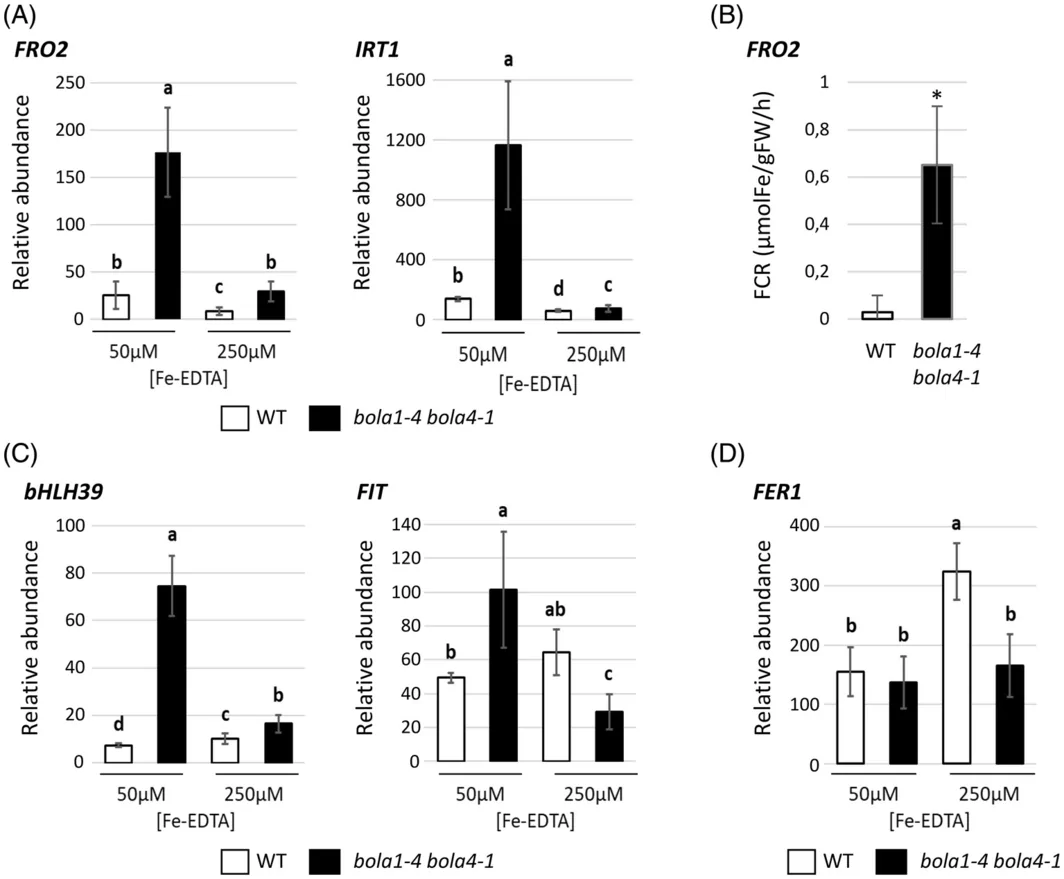
The *bola1‐4 bola4‐1* double mutation triggers an iron‐deficiency response in *Arabidopsis thaliana*. (A) Iron uptake‐related genes expression in seven‐day‐old wild‐type (WT) and *bola1‐4 bola4‐1* roots excised from seedlings grown vertically on ½ MS medium containing 1% sucrose, under control (50 μM Fe‐EDTA) or high‐iron (250 μM Fe‐EDTA). Root transcripts were quantified by RT‐qPCR, and gene expression was normalized to the *PP2AA3* reference gene. (B) FRO2 ferric‐chelate reductase (FCR) activity measured in total root protein extracts from WT and *bola1‐4 bola4‐1* seedlings grown vertically on ½ MS medium under sufficient iron conditions (50 μM Fe‐EDTA). Means were compared using a *t*‐test (*P* < 0.01). Data are from 6 independent biological replicates (pools of 10 WT and 30 *bola1‐4 bola4‐1* roots per biological replicate), each performed in technical triplicate. (C, D) Transcript abundance of genes involved in the regulation of iron homeostasis (C) and iron storage (D), assessed as described in (A). (A, C, D) Data are from 4 independent biological replicates (pools of 25 WT and 75 *bola1‐4 bola4‐1* roots per biological replicate), each performed in technical triplicate. Means within each condition sharing the same letter are not significantly different according to a Kruskal‐Wallis test followed by post hoc analysis (Conover, 1999) with Holm correction (*P* < 0.01). For all panels, error bars represent means ± SD.

The expression of *FER1* (*FERRITIN 1*), a marker gene of the Fe excess response involved in the transient storage of this micronutrient (Gao et al., 2020; Tissot et al., 2019), was also analyzed (Figure 5D). RT‐qPCR analysis revealed that under control condition, *FER1* expression in the *bola1‐4 bola4‐1* double mutant was similar to that of the WT. In contrast, under high‐Fe condition, no induction of *FER1* expression was observed in the *bola1‐4 bola4‐1* double mutant in contrast to what was observed for the WT.

Similar analyses were also conducted on 12‐day‐old plants, revealing similar gene expression patterns (Figure S14A). The main difference was observed for *FIT*, whose expression remained higher in the *bola1‐4 bola4‐1* mutant compared to the WT regardless of Fe conditions, and *FER1* that displayed opposite trends (Figure S14A).

Altogether, these data suggest that the *bola1‐4 bola4‐1 double* mutant remains responsive to fluctuations in Fe availability. However, the absence of *BOLA1* and *BOLA4* proteins in the mutant triggers a Fe deficiency response while reducing the perception of Fe excess.

### The *bola1 bola4* double mutant shows defects in Fe distribution

Since the expression of Fe deficiency and Fe excess marker genes was altered in the *bola1‐4 bola4‐1 double* mutant, we investigated whether Fe level and its distribution within the plant were also affected. To this end, Fe content was assessed in roots and shoots separately. While the *bola1‐4 bola4‐1 double* mutant and the WT plant displayed similar Fe content in the shoot, they differed significantly in root Fe levels, with the mutant accumulating approximately half the Fe level found in the WT (Figure 6A). Similar analysis of desiccated seeds indicated that this alteration in Fe content occurs after germination, as no significant differences between genotypes were observed in seeds (Figure 6B).

**Figure 6:**
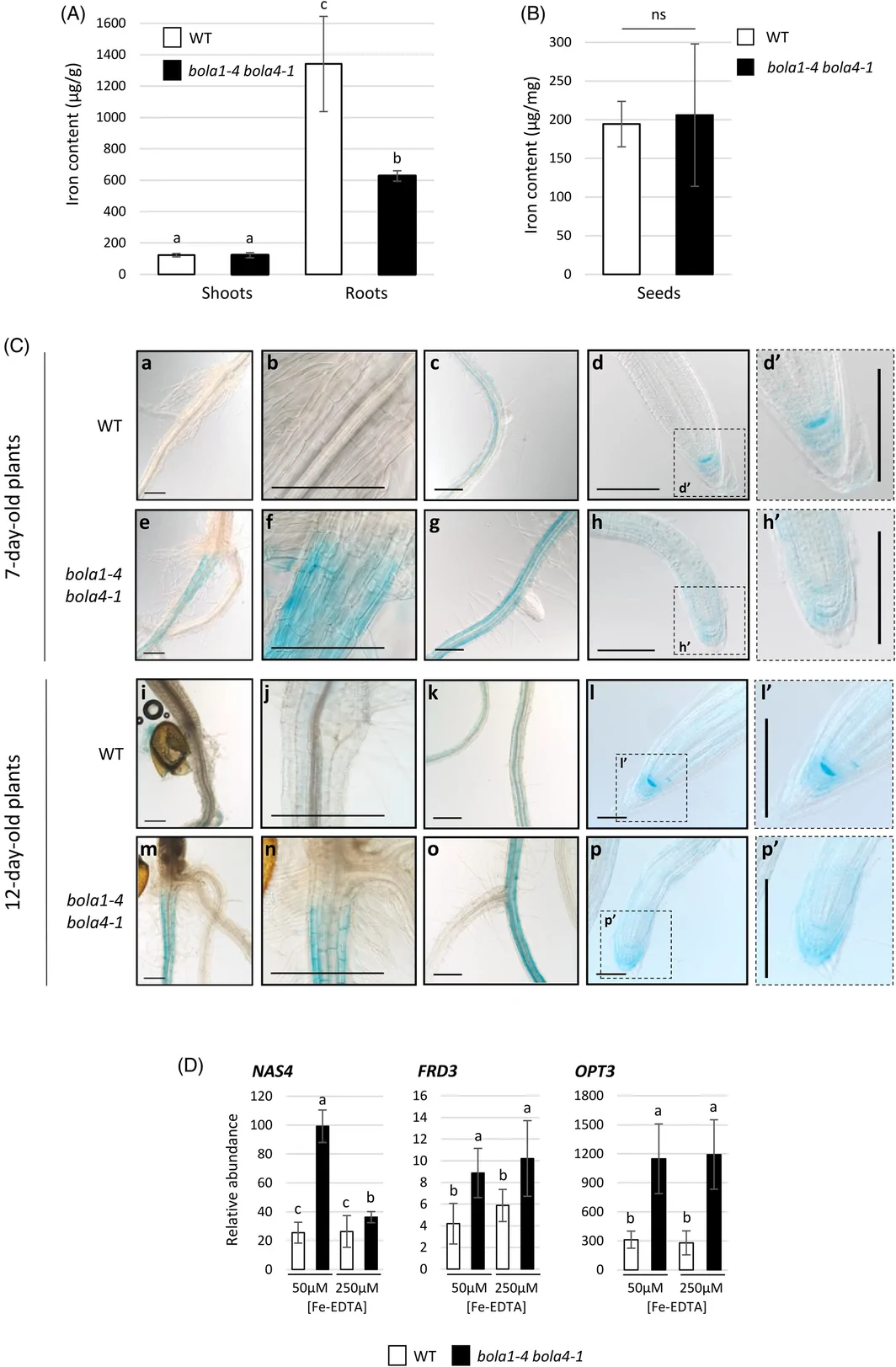
The double *bola1‐4 bola4‐1* mutation alters iron distribution in roots in *Arabidopsis thaliana*. Comparison of the total iron content between the wild‐type (WT) and *bola1‐4 bola4‐1* mutant lines was measured (A) on shoots and roots excised from 12‐day‐old plants, and (B) on desiccated seeds. Means within each condition with the same letter are not significantly different according to according to Wilcoxon test (*P* < 0.05). Error bars show ±SD. n = 4 independent biological repeats, with each experiment done in triplicate. (C) Perls staining was used to detect iron in seven‐day‐old and 12‐day‐old wild‐type (a‐d, i–l) and *bola1‐4 bola4‐1* (e–h, m–p) plants. (a, e, I, m) General views of the plantlets from the bottom of the hypocotyl to the upper part of the primary root. (b, f, j, n) Collar areas. (c, g, k, o) Primary roots in the maturation zone. (d, h, l, p) Root tips, with insets (d', h', l', p') highlighting the meristematic and elongations zones. Magnifications of the insets (panels framed in dotted lines) are also shown to the right of the corresponding panels (d, h, l, p). Scale bars = 0.2 mm. (D) Transcript abundance of iron transport marker genes in roots and shoot in seven‐day‐old WT (white bars) and *bola1‐4 bola4‐1* mutant (black bars). The expression of genes involved in nicotianamine transport (*NAS4*) and citrate efflux (*FRD3*) in root, and phloem‐specific iron (*OPT3*) transport in shoot was assessed by RT‐qPCR under control (50 μM Fe‐EDTA) or high (250 μM Fe‐EDTA) iron conditions, using the *PP2AA3* gene as control. Means within each condition with the same letter are not significantly different according to Kruskal‐Wallis test with post hoc analysis (Conover, 1999), *P* < 0.01, with Holm corrections. Error bars show means ± SD. Four independent biological repeats were performed, with each experiment done in technical triplicates. A biological repeat required 25 WT and 75 *bola1‐4 bola4‐1* seedlings.

We next examined the distribution of Fe in three‐day‐old WT and *bola1‐4 bola4‐1 mutant* seedlings using Perls/DAB staining. At this stage, no significant morphological differences or Fe distribution variations were observed between the two genotypes (Figure S15). In contrast, Perls staining without DAB amplification was sufficient to reveal a strong accumulation of Fe just below the collar of the seven‐day‐old *bola1‐4 bola4‐1* roots (Figure 6C, panels e,f), while the hypocotyl did not retain the stain. In comparison, no detectable Fe was observed in these regions of the WT (Figure 6C, panels a,b). It should be noted that the intense staining in the root of the mutant below the collar could be explained by the extremely reduced size of the primary root of *bola1‐4 bola4‐1* compared with that of the wild‐type, in which iron is distributed more evenly. Fe also accumulated in the maturation zone of the *bola1‐4 bola4‐1 root* (Figure 6C, panel g), at a higher extent than in the WT in the same area (Figure 6C, panel c). Conversely, Perls staining allowed the detection of Fe in the root meristem of the WT (Figure 6C, panels d,d'), while it could be detected only as a faint diffuse pattern in this region of the mutant root (Figure 6C, panels h,h').

All of these patterns persisted in 12‐day‐old plants (Figure S16A), but were attenuated under Fe‐excess conditions (Figure S16B, panels m, n) compared with Fe‐sufficient conditions (panels e‐f). Notably, they closely resembled the distribution observed in WT roots exposed to the same high‐Fe conditions (panels i–j). Fe accumulation at the maturation zone of the mutant root (panel g) also decreased under high‐Fe conditions (Figure S16A,B, panel o) while becoming newly detectable at the mutant root tip (Figure S16B, panels p, p’ versus h, h'). This pattern resembled that of the WT plant, although to a lesser extent (Figure S16B, panels d, d' versus l, l'). These observations indicated that Fe excess partially rescued Fe distribution in the mutant root.

To get insights into the molecular response associated with the modification of Fe partitioning in the *bola1‐4 bola4‐1* root, we next analyzed the expression of *NAS4* (*NICOTIANAMINE SYNTHASE 4*), *FRD3* (*FERRIC REDUCTASE DEFECTIVE 3*), and *OPT3* (*OLIGOPEPTIDE TRANSPORTER 3*). *NAS4* and *FRD3* are important genes for Fe homeostasis since they play a key role in Fe transport and partitioning via the phloem and xylem, respectively (Koen et al., 2013; Roschzttardtz et al., 2011). *OPT3*, which is mostly expressed in aerial tissues, plays a key role in systemic signaling of Fe deficiency from shoots to roots in Arabidopsis (Hendrix, 2023; Mendoza‐Cózatl et al., 2014). RT‐qPCR analyses revealed that the expression of the three genes was increased in the *bola1‐4 bola4‐1* root (*NAS4*, *FRD3*) and shoot (*OPT3*) under Fe sufficiency, when compared to the WT (Figure 6D), and maintained high under Fe excess conditions. Similar patterns were observed in older plants, except for *OPT3* (Figure S14B). These data are in agreement with *FRO2*, *IRT1*, *FIT* and *bHLH39* expression analyses (Figure 5), reinforcing the hypothesis that the *bola1‐4 bola4‐1 double mutant perceives* Fe deficiency signals resulting in the stimulation of genes involved in Fe translocation systems.

### BOLA1 and BOLA4 activity in root is essential for proper plant development

To determine whether the altered Fe distribution observed in *bola1‐4 bola4‐1* mutant tissues results from defects in the shoot, the root, or both, we performed reciprocal grafting experiments between WT plants and the *bola1‐4 bola4‐1* double mutant. In these experiments, each genotype was used interchangeably as rootstock or scion, and all grafted plants were genotypically validated by RT‐PCR analysis (Figure S17A). We observed that grafting a WT root system onto the rosette of the *bola1‐4 bola4‐1* mutant was the only treatment that restored growth to levels comparable to the positive control (Figure 7A). This rescued phenotype persisted into the mature stage (Figure S17B).

**Figure 7:**
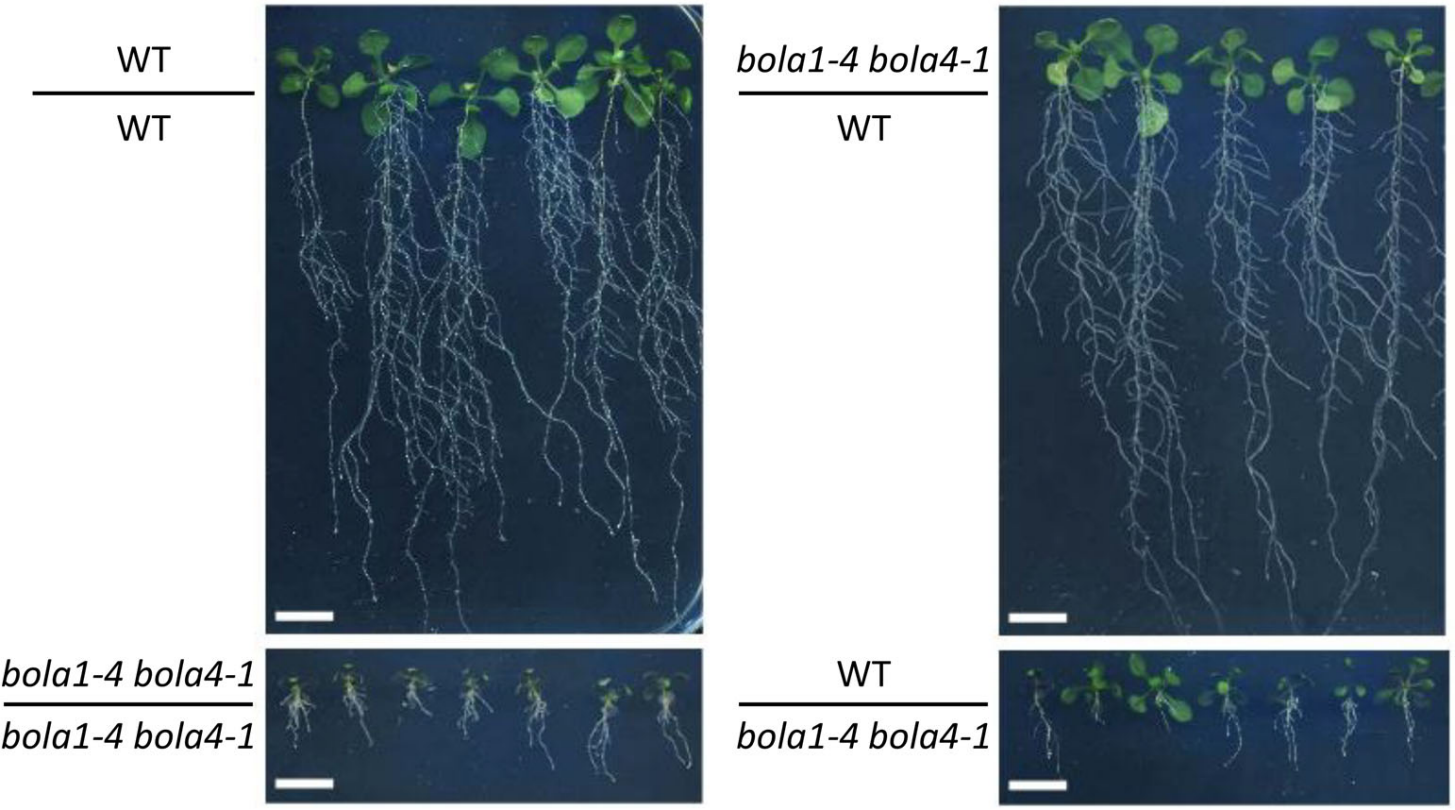
Grafting a wild‐type root to *bola1‐4 bola4‐1* shoot restores normal growth. Shoot scion and rootstock genotypes are indicated above and below the lines, respectively. WT/WT and *bola1‐4 bola4‐1/bola1‐4 bola4‐1* grafted plants were used as positive and negative controls, respectively. Plants were photographed 2 weeks post‐grafting. The images are representative of the results obtained from 5 independent biological replicates, with each grafting experiment requiring around 200 plants of each genotype. Scale bars = 1 cm. Validation of grafted genotype by RT‐PCR and pictures of 5‐week‐old plants are provided in Figure S17 and Table S2.

These results demonstrate that the *bola1‐4 bola4‐1* dwarf phenotype is due to some impaired function of BOLA1 and BOLA4 in the root system and indicate that the transfer of root signals over long distances to modulate the whole‐plant growth relies on the activity of both proteins.

## DISCUSSION

Like every organism, plants rely on Fe for essential biological processes. This is for instance the case for photosynthesis, respiration, and chlorophyll biosynthesis, making Fe a crucial micronutrient for plants. Thus, Fe deficiency leads to severe plant health issues, including chlorosis, stunted growth, and overall physiological decline. Conversely, excessive Fe can be toxic, generating Reactive Oxygen Species (ROS) that negatively affect biomass (Briat et al., 2015). Maintaining Fe homeostasis is therefore critical, requiring precise regulation of its uptake, transport, storage, and utilization through transcriptional activities (reviewed by Gao & Dubos, 2021; Li et al., 2023). Interestingly, certain Fe‐containing proteins, particularly [Fe‐S] proteins, not only depend on Fe availability but also play a crucial role in maintaining Fe homeostasis. For example, AtNEET, a [2Fe‐2S] binding protein localized in the chloroplast and the mitochondria (Nechushtai et al., 2012), has been suggested to function as an Fe sensor in plastids, contributing to the regulation of Fe balance to protect both plastids and cytosol from cellular stresses, such as nutrient deprivation or Fe overload (Zandalinas et al., 2020). Arabidopsis GRXS17 and BOLA2, two members of the cytosolic [Fe‐S] cluster assembly machinery (Couturier et al., 2014), also play a role in Fe homeostasis. GRXS17 is essential for tolerance to Fe deficiency (Cheng et al., 2020), while BOLA2 behaves as a repressor in the response to Fe excess (Qin et al., 2015). To date, BOLA2 is the only member of its family to have undergone functional characterization. In this study, we have characterized BOLA1 and BOLA4, two paralogs of BOLA2, whose physiological role remained unknown in plants.

An initial analysis of gene expression in Arabidopsis tissues revealed that, like *BOLA2* (Qin et al., 2015), both *BOLA1* and *BOLA4* are expressed throughout the whole‐plant body. Similar to *bola2* mutant, knock‐out alleles for *BOLA1* (*bola1‐3* and *bola1‐4*) and *BOLA4* (*bola4‐1* and *bola4‐2*) showed no major phenotypic differences under Fe‐sufficient conditions compared to the WT, except for a slight reduction in root length observed in *bola4* mutants. While BOLA1 and BOLA4 appeared dispensable for plant survival, Fe storage in seeds or the germination process, *bola1 bola4* double knock‐out mutants displayed a severe dwarf phenotype characterized by early primary root arrest and a fasciculate root architecture, which supports the likelihood of functional redundancy between BOLA1 and BOLA4. A similar redundancy was also observed in yeast, where mitochondrial BOL1 and BOL3 exhibit overlapping functions (Uzarska et al., 2018). This raised questions about the subcellular localization of the Arabidopsis BOLA1, first believed to be exclusively plastid‐localized based on transient expression assays in protoplasts, while BOLA4 was found in both the plastid and mitochondria (Couturier et al., 2014; Qin et al., 2015). By reassessing BOLA1 localization in Arabidopsis protoplasts, we show here that, contrary to previous reports, BOLA1 is not restricted to chloroplasts but also localizes to mitochondria, at least when *BOLA4* expression is low. This finding being in adequacy with proteomic studies that identified BOLA1 peptides in Arabidopsis mitochondria (Fuchs et al., 2020; Niehaus et al., 2020; Senkler et al., 2017). BOLA1 mitochondrial targeting in Arabidopsis also aligned with findings in all studied eukaryotes to date (da Silva et al., 2023; Talib & Outten, 2021; Uzarska et al., 2016). Our finding also indicates that, while BOLA1 and BOLA4 seem to be equally abundant in plastids, BOLA4 appears as the major isoform in plastids and mitochondria in plant tissues, consistent with the slight reduction in root growth in the two *bola4* mutants. Interestingly, we found that *BOLA1* expression was not induced in the *bola4* mutant, suggesting that trace amounts of BOLA1 would be sufficient to compensate BOLA4 absence. Another hypothesis could be that the absence of BOLA4 triggers BOLA1 presence in the mitochondria. Overall, our data indicate that BOLA1 and BOLA4 are dually targeted to plastids and mitochondria, where they likely share redundant important functions.

In addition to growth defects, the *bola1‐4 bola4‐1* double mutant exhibited significant disruption in multiple aspects of Fe homeostasis regulation. This was first evident from the induction of *bHLH39, FIT, FRO2*, and *IRT1* gene expression under both Fe‐sufficient and Fe excess conditions, suggesting a persistent Fe deficiency response in the mutant. This response was attenuated over time but maintained higher in the mutant compared to the WT plant. These data were consistent with the lower Fe content present in the mutant root system, mostly at the root tip according to Perls staining, and despite the strong accumulation of Fe under the root collar in the mutant root, visible in *bola1‐4 bola4‐1* mutant from the 6^th^ day of growth. Similarly, we observed an increased expression of *NAS4* and *FRD3*, two other marker genes of the Fe deficiency response under the same Fe‐sufficient and Fe excess conditions. NAS4 is the enzyme that catalyzes the biosynthesis of nicotianamine, the main chelator of ionic Fe in the phloem and is necessary for the proper translocation of Fe from the root to the aerial tissues (Klatte et al., 2009; Koen et al., 2013). FRD3 is a plasma membrane citrate efflux transporter that plays an important role in xylem Fe loading (Roschzttardtz et al., 2011). For these genes, expression levels remained consistently high over time. In contrast, the expression of *FER1*, a marker gene of Fe excess, was not induced in the mutant under high‐Fe conditions, and remained expressed at low levels compared to the WT over time. These data suggest that Arabidopsis plants affected by the *bola1 bola4* mutations experience Fe deficiency in the root apparatus and adjust the expression of Fe‐deficiency marker genes accordingly but have reduced capacities to perceive excess Fe in the media. OPT3, a key player in the systemic signaling of Fe deficiency from shoot to roots (Mendoza‐Cózatl et al., 2014; Zhai et al., 2014), was also induced in the leaf of *bola1‐4 bola4‐1* mutant. Strikingly, no difference in shoot Fe content was observed, suggesting that not all organs are affected by the *bola1‐4 bola4‐1* double mutation. This conclusion is supported by the absence of differences in leaf pigment content, photosynthetic activity, and Fe content in aerial parts between WT plants and the *bola1‐4 bola4‐1* mutant (Figure S17). Grafting experiments further indicated that the defect is restricted to the roots of the *bola1‐4 bola4‐1* mutant, highlighting the crucial role of BOLA1 and BOLA4 in this organ. It is noteworthy that the *bola1‐4 bola4*–1 mutant, which shows severe defects in primary root development, survives thanks to its ability to rapidly initiate numerous secondary roots emerging from the root collar. We thus hypothesize that the Fe demand, high in mitochondria and plastids, becomes particularly acute in this region, where many secondary roots are formed. This would explain the induction of the Fe deficiency responses, and the strong Fe accumulation we observed in this region. The cumulative absence of BOLA1 and BOLA4 may also affect the long‐distance signaling of certain compounds, including growth signals, from the root to the shoot, as highlighted by the overall dwarf phenotype of the double mutant and its general growth delay. Thus, long‐distance Fe transport in the mutant appears to be specifically mobilized to supply the root collar, where its immediate need for Fe to ensure survival is most critical. Consistent with this hypothesis, supplementation of the growth medium with 250 μM Fe partially rescued the short‐root phenotype of the mutant and alleviated its stunted rosette growth. This treatment also partially restored a WT‐like Fe distribution along the mutant root, with Fe levels remaining high at the collar, and partially reduced the elevated expression of the analyzed Fe marker genes. Overall, these data indicate that BOLA1 and BOLA4 activities are of central importance for Fe partitioning in the root and balanced Fe status signaling throughout the plant body.

Besides Fe acquisition and distribution among tissues, Fe transport across subcellular compartments is crucial for maintaining proper Fe homeostasis. In particular, mitochondria and chloroplasts require significant amounts of Fe, which is required by many proteins involved in the photosynthetic pathway and the respiratory electron transport chains. For instance, chloroplasts are the most Fe‐rich organelle, with 60–80% of the total leaf Fe found in chloroplasts (Shikanai et al., 2003; Terry & Low, 1982). Due to their localization (this study), the absence of BOLA proteins could lead to Fe imbalanced levels in plastids and mitochondria, resulting from defective [Fe‐S] cluster assembly and delivery. This is likely to have widespread effects on various [Fe‐S] client metabolic pathways within these compartments, aligning with the severe phenotype observed in the *bola1 bola4* double mutants. The regulation of Fe homeostasis in plastids and mitochondria, as well as on a systemic scale between root and shoot, must be precisely monitored and interpreted to ensure that one or more signals are relayed to the nucleus, allowing it to generate an appropriate adaptive response. In Arabidopsis, several plastidial and mitochondrial proteins, such as Fe transporters, may contribute to Fe signaling pathways. For instance, FPN3 (FERROPORTIN 3) is an Fe exporter dual‐targeted to mitochondria and chloroplasts, expressed in leaves independent of Fe conditions but whose transcripts accumulate under Fe deficiency in roots (Kim et al., 2021). *fpn3* mutations induce higher Fe levels in both compartments compared to a WT plant but mostly affect mitochondria ultrastructure, suggesting that FPN3 is a major Fe exporter in this compartment. NEET proteins, with their labile [2Fe‐2S] clusters, can also contribute to regulate Fe homeostasis (Tamir et al., 2015). While AtNEET's function remains unclear, its disruption impairs Fe acquisition and distribution (Nechushtai et al., 2012; Zandalinas et al., 2020). AtNEET transfers [2Fe‐2S] clusters to mitochondria (Nechushtai et al., 2012) and cytosol via DRE2 (Zandalinas et al., 2020), and is also found in the plastid stroma (Su et al., 2013), indicating its role in Fe metabolism across cellular compartments. Whether the *bola1 bola4* mutation modifies the activities of such plastidial and mitochondrial important components will require further investigation.

In Arabidopsis, several mutants affected in proteins acting in [Fe‐S] delivery exhibit either a dwarf phenotype, similar to *bola1 bola4* mutants, such as *nfu2* and *nfu3*, which are components of plastids (Nath et al., 2016; Touraine et al., 2019), or lethal phenotypes, such as *hcf101*, which affects plastids (Lezhneva et al., 2004), and *grxs15*, *nfu4* and *nfu5* (Moseler et al., 2021; Przybyla‐Toscano et al., 2022; Ströher et al., 2016), which affect mitochondria. This suggests that disruptions in plastidial or mitochondrial [Fe‐S] transport often severely impair plant growth. BOLA1 and BOLA4 are known interactors of class II GRXS that share the same subcellular compartments in Arabidopsis, namely GRXS15 in the mitochondria, and GRXS14 and GRXS16 in the plastid (Couturier et al., 2014). GRXS15 is the sole GRXS isoform in the mitochondria and is mandatory for embryo development (Ströher et al., 2016) and for [Fe‐S] assembly in proteins involved in the TCA cycle and respiration (Moseler et al., 2015). Interestingly, a *grxs15‐3* mutant plant exhibits a severe dwarf phenotype (Moseler et al., 2015), resembling that of the *bola1 bola4* mutant. This suggests that beyond their physical interaction capabilities, BOLA1 and BOLA4 could work in the same pathways, independently or in conjunction with GRXS15. On the other hand, plants lacking either GRXS14 or GRXS16 in plastids did not show any obvious phenotype (Rey et al., 2017), like *bola1* and *bola4* single mutants. Nevertheless, the rosette weight of lines knocked out for *GRXS14* and knocked down for *GRXS16* was reduced by more than 20%, indicating that the combined diminution of GRXs content in plastids also impairs growth (Rey et al., 2017). However, the lack of *grxs14 grxs16* double knock‐out mutants makes it difficult to determine the physiological significance of potential GRXS/BOLA complexes in the plastid. It is noteworthy that plastids and mitochondria house nearly a hundred [Fe‐S] client proteins, many of which play essential roles in various functions critical for plant growth and development. While not all of these proteins acquire their [Fe‐S] centers through GRXs, the potential role of BOLA proteins in modulating GRXS activity within these compartments could lead to widespread pleiotropic defects, which may explain the double *bola1 bola4* phenotype. To support this hypothesis, our preliminary results indicate that the combined absence of BOLA1 and BOLA4 leads to a slight increase in the abundance of the Fe‐S cluster shuttle protein GRXS15 in the mitochondria of the *bola1‐4 bola4‐1* double mutant, and to the decrease of the abundance of the mitochondrial succinate dehydrogenase subunit SDH2 and the cytosolic ACONITASE1 (Figure S19), two Fe‐S proteins that depend on the mitochondrial Fe‐S cluster assembly machinery. Further investigations are therefore needed to ascertain the exact contribution of BOLA1 and BOLA4 to GRXS functions in plants, by notably identifying the whole set of proteins they target to deliver [Fe‐S] clusters, alone or in collaboration with Fe‐S shuttle proteins.

Taken together, the data presented in this study shed new light on the function and cellular localization of BOLA1 and BOLA4 and highlight the importance of these two proteins in plant growth and Fe partitioning. These findings open interesting perspectives for future studies aimed at deciphering whether, and if so how, BOLA1 and BOLA4 extend their functions toward [Fe‐S] cluster assimilation and Fe homeostasis in plants.

## MATERIALS AND METHODS

### Plant materials, growth conditions and transformation

*Arabidopsis thaliana* var. Columbia 0 (Col‐0) was used as the wild‐type (WT) line in the study. The *bola1‐3* (Salk_059678), *bola1‐4* (*Salk_082155*), and *bola4‐1* (*Salk_050846*) T‐DNA insertion lines were obtained from the Nottingham Arabidopsis stock center (https://arabidopsis.info/) (Figure S3A). Homozygous lines of *bola1‐3, bola1‐4*, *and bola4‐1* were isolated by PCR genotyping and by sequencing the T‐DNA insertion sites from the left T‐DNA border (Tables S1 and S3; Figure S4). In addition, we generated a second homozygous loss‐of‐function mutant allele for *BOLA4* by Crispr‐Cas9 genome editing in the Col‐0 genetic background (Methods S1). A homozygous *cr‐bola4* line, validated by sequencing, was backcrossed with the WT to remove the Cas9 cassette, and the homozygous *bola4‐2* mutant allele was selected (Figures S3B,C and S4). All details about the sgRNA guide sequence, position, and off‐target analysis are given in Table S5 and Figure S6.

Double homozygous mutants (*bola1‐4 bola4‐1* and *bola1‐3 bola4‐2*) were obtained by crossing the corresponding *bola* single mutants and verified through PCR genotyping and sequencing with the primers listed in the Table S1. The absence of *BOLA1* and/or *BOLA4* gene expression was also validated by real‐time quantitative reverse transcription PCR (RT‐qPCR) analysis (Table S2). All transgenic lines generated in the frame of this study are described in the Cloning section.

Arabidopsis seeds were surface‐sterilized with a mix of ethanol (96%, v/v) and NaClO (0.15%, w/v) for 5 min, rinsed three times with 96% ethanol, and sown on ½‐strength Murashige and Skoog (½MS) medium containing 1% sucrose and solidified with 1% (w/v) agar (pH 5.7). After vernalization for 48 h at 4°C, plants were grown in controlled growth chambers with standard Arabidopsis growth conditions (22°C/20°C for day/night temperature, respectively, 65% relative humidity, and long‐day regime with 8 h dark /16 h light providing 150 μmol m^−2^ s^−1^ light intensity). Sufficient iron (Fe) availability in the growth media was obtained using 50 μM Fe‐EDTA, while Fe excess conditions were established by supplementing the ½MS media with either 250 μM Fe‐EDTA.

### Cloning

All the constructs obtained in the study were systematically checked by sequencing. Primers and vectors used for cloning and sequencing are listed in the Table S4.

All DNA fragments were cloned using the Gateway® cloning technology (Life Technologies™, Invitrogen), with BP and LR Clonase™ II Enzyme mixes for recombination into the pDONR207™ as the entry vector and into various destination vectors, respectively.

For functional complementation of the *bola1 bola4* mutant with *BOLA1* or *BOLA4* genes, the entire *BOLA* loci, including 2 kb promoter (*proBOLA1* or *proBOLA4*) and the whole genomic *BOLA* sequences, including their respective 3′ noncoding region (*gBOLA*), were cloned into the pGWB1 destination vector to give rise to *proBOLA1:gBOLA1* and *proBOLA4:gBOLA4* constructs.

To create vectors allowing the expression of BOLA1 and BOLA4 fused to the β‐GLUCURONIDASE (GUS) enzyme (BOLA1‐GUS and BOLA4‐GUS), the entire *BOLA* loci (without the stop codon) were cloned into the pGWB3 destination vector containing the bacterial GUS‐encoding *uidA* gene to give rise to *proBOLA1:gBOLA1‐uidA* and *proBOLA4:gBOLA4‐uidA* constructs.

To create vectors allowing the expression of BOLA1 and BOLA4 fused to the GREEN FLUORESCENT PROTEIN (GFP), the entire *BOLA* loci without its stop codon were also cloned into the pGWB4 destination vector expressing GFP to give rise to *proBOLA1:gBOLA1‐GFP* and *proBOLA4:gBOLA4‐GFP* constructs. The *proUBQ10:gBOLA1‐GFP* construct used to reassess the subcellular localization of BOLA1 in protoplasts was obtained after amplification of the genomic *BOLA1* encoding region without its stop codon and recombination into the pUBC‐DEST vector according to (Grefen et al., 2010).

For Arabidopsis Col‐0 transformation, 3‐week‐old plants grown under long‐day regimes were transformed using the *Agrobacterium tumefaciens*‐mediated floral dip method (Clough & Bent, 1998). Transformants were selected on ½MS medium containing 50 μg/mL hygromycin and the presence of the transgene was confirmed by PCR (Table S3) and by sequencing.

### Protoplast isolation and subcellular localization analyses

Arabidopsis protoplasts were isolated and transfected with *proBOLA:BOLA‐GFP* or *proUBQ10:BOLA1‐GFP* plasmids as described by Yoo et al., 2007 without vacuum infiltration. After transfection, mitochondria were stained with 200 nM MitoTracker® Orange CMTMR (Invitrogen, Thermo Fisher Scientific Biosciences GmbH, St. Leon‐Rot, Germany) for 3 min and washed twice with the WI buffer. The GFP fluorescence in Arabidopsis protoplasts was recorded between 500 and 550 nm with a SP8 laser‐scanning confocal microscope (Leica Microsystems) after excitation of the GFP with an argon laser at 488 nm. The fluorescence of the MitoTracker® Orange CMTMR was recorded between 580 and 620 nm after excitation with a diode‐pumped solid‐state laser at 561 nm.

Transgenic plants expressing BOLA‐GFP fusion proteins were analyzed after 7 days of growth on ½ MS medium using a Stellaris laser‐scanning confocal microscope (Leica Microsystems). Plants were stained with propidium iodide (PI) coupled or not with 200 nM MitoTracker® Orange CMTMR stain for 5 min. GFP fluorescence was recorded as described above. PI was recorded between 580 and 640 nm after excitation by a white laser at 561 nm. Chlorophyll autofluorescence was used for colocalization with plastids.

Confocal images were obtained at high resolution using the Leica LAS X software and subsequently transformed into TIFF files with the Adobe Photoshop CS4 software.

### Gene expression analyses

Arabidopsis tissues required for RT‐PCR and RT‐qPCR analyses were harvested and immediately frozen in liquid nitrogen. Total RNAs were extracted using the NucleoSpin® RNA purification kit (MACHEREY‐NAGEL, Hoerdt, France) and further digested with the rDNase at room temperature for 15 min according to the manufacturer's protocol. First strand cDNA synthesis was performed using 1 μg of total RNAs treated with DNase and the RevertAid first strand cDNA synthesis kit (Thermo Fisher Scientific Biosciences GmbH) according to the manufacturer's instructions.

RT‐PCR experiments were performed using equal amounts of cDNA obtained as described above with the DreamTaq enzyme (Thermo Fisher Scientific) over 30 cycles, with the *PROTEIN PHOSPHATASE2A SUBUNIT A3* (*PP2AA3*, AT1G13320) gene as control (Czechowski et al., 2005; Tissot et al., 2019).

RT‐qPCR amplifications were conducted using the TB Geen® Premix Ex Taq™ (TAKARA Bio Europe, Saint‐Germain‐en‐Laye, France) in a LightCycler 480® (Roche Diagnostics, Meylan, France) using freshly prepared cDNAs. The cycle threshold (Ct) method was used to relatively quantify transcript accumulation levels. Expression data were normalized to the *PP2AA3* gene expression. All primers used for RT‐qPCR reactions are shown in Table S2. All analyses were done in 4 independent biological replicates.

### Histochemical GUS staining

Transgenic Arabidopsis plants containing *proBOLA1* or *proBOLA4* fused upstream from the *uidA* gene were harvested and immediately infiltrated during 1 h under vacuum with the GUS staining buffer (100 mM sodium phosphate pH 7.2, 10 μM 5‐bromo‐4‐chloro‐3‐indolyl‐β‐D‐glucuronic acid, 10 mM EDTA pH 8 and 0.1% Triton X‐100). Infiltrated tissues were incubated during 16 h at 37°C, cleared in an EtOH 95% solution at 4°C during 24 h, and then analyzed using a bright‐field microscope (Axio Zoom V.16, Zeiss) and an Olympus BX61 microscope. Four independent lines per construct were analyzed, with each line including five experimental replicates.

### Histochemical staining of Fe with Perls/DAB procedure

Perls staining and DAB/H_2_O_2_ intensification were performed as described in Brumbarova and Ivanov (2014). Plantlets were first fixed in a methanol:chloroform:glacial acetic acid (6:3:1, v/v/v) solution for 2 h under vacuum, followed by three washes with distilled water. Samples were then incubated in a staining solution (K_4_Fe(Cn)_6_ 4% w/v, HCl 4% v/v) for 25, 3, or 4 min for 3, 7, or 10‐day‐old plants, respectively, followed by three washes with distilled water. Next, the samples were incubated at room temperature for 1 h in a preparation solution (NaN_3_ 0.01 M, H_2_O_2_ 0.3% v/v in methanol) followed by three washes with phosphate buffer (Na_2_HPO_4_ 0.1 M, NaH_2_PO_4_ 0.1 M pH 7). For intensification, samples were incubated in an intensification solution (0.1 M phosphate buffer, pH 7) containing 0.05% (w/v) DAB (Sigma‐Aldrich Chimie, Saint‐Quentin‐Fallavier, France), 0.005% (v/v) H_2_O_2_, and 0.005% (w/v) CoCl_2_ for 3 min or 5 min for 3‐day‐old and 10‐day‐old seedlings, respectively. For 7‐day‐old seedlings, the intensification solution was adapted (phosphate buffer 0.1 M pH 7, DAB (Sigma‐Aldrich Chimie) 0.005% w/v, H_2_O_2_ 0.005% v/v and CoCl_2_ 0.005% w/v) and the incubation time was increased to 18 min. The reaction was stopped by adding water. For Perls staining, the plants were observed in a clearing solution (chloral‐hydrate 0.01% w/v, 15% glycerol v/v) using an Olympus BX61 microscope. For Perls/DAB staining, whole‐plant observations were performed in water with bright‐field microscopy (Axio Zoom V.16, Carl Zeiss, Rueil‐Malmaison, France). Perls/DAB‐stained tissues are representative of at least five independent biological replicates.

### Fe quantification

Fe quantification in Arabidopsis root and shoot was performed using 10 mg of lyophilized materials, microwave digested in 10 mL of HNO_3_ for 15 min at 180°C. After filtration through MN 640 W paper, Fe content was measured by inductively connected plasma mass spectrometry (ICP‐MS; Thermo Fisher Scientific Inc., Waltham, MA, USA). For both tissues, four independent biological experiments were performed using approximately 110 (WT) and 360 (*bola1‐4 bola4‐1*) isolated roots, and 50 (WT) and 130 (*bola1‐4 bola4‐1*) isolated shoots, with each measurement being done in triplicates.

Fe quantification in Arabidopsis seeds was performed using 10 mg of dry seeds hydrolyzed in a solution of 50% (v/v) nitric acid and 17% (v/v) H_2_O_2_ for 8 to 16 h at 85°C for 8 h. Once the solution became completely transparent, Fe concentration was quantified using microwave plasma‐atomic emission spectroscopy (MP‐AES) with an Agilent 4210 MP‐AES device.

### Plant grafting

Arabidopsis plantlet grafting was performed under a sterile hood following Marsch‐Martínez et al. (2013) with slight modifications. Five‐day‐old Arabidopsis seedlings, grown on standard ½MS medium (1% sucrose, 1% agar) under long‐day conditions (16 h at 22°C, 8 h at 20°C), were cut just above the primordial root collar using a sterile surgical blade. The aerial parts and root systems were separately transplanted onto a grafting‐specific medium (½MS with 0.5% sucrose and 1% agar), ensuring direct contact between the scion and selected rootstock. The grafted plants were then incubated under short‐day conditions (8 h light, 16 h dark) at 27°C for 7 days. Subsequently, they were transferred to regular ½MS medium and maintained under long‐day conditions for additional days before observation.

### Ferric‐chelate reductase activity measurements

FCR activity was measured according to Gao et al. (2020). Ten mg of fresh root tissues were incubated for 1 h in the dark with gentle shaking in 2 mL of FCR buffer (100 mM Fe^3+^‐EDTA, 300 mM ferrozine, and 10 mM MES, pH 5.5). The concentration of Fe^2+^‐ferrozine complex (which displays a purple coloration) was determined after reading absorbance at 560 nm using a Clariostar microplate reader (BMG LabTech).

### Photosynthetic pigment content and photosynthetic activity

Leaves (15 mg) were harvested from 10‐day‐old plants grown on ½ MS medium containing 1% sucrose, and their pigments were extracted in 1.5 mL of 100% acetone at room temperature, in the dark under gentle shaking for 72 h. Absorbance of chlorophyll *a*, chlorophyll *b*, and carotenoids was measured at 644, 661, and 480 nm, respectively, using a Clariostar microplate reader (BMG LabTech), and pigment contents calculated according to Lichtenthaler (1987). Eight independent biological replicates were analyzed, each requiring approximately 15 WT and 22 *bola1‐4 bola4‐1* aerial parts.

Photosynthetic activity was measured using a FluorCam7 camera (Photon System Instruments) according to Maxwell and Johnson (2000). The maximal quantum yield of PSII (QYmax) was measured on dark‐adapted plants as Fv/Fm = (Fm‐Fo)/Fm, where Fm is the maximum fluorescence level obtained with a pulse of intense white light and Fo is the initial fluorescence level in the dark. Seventy independent 10‐day‐old seedlings for each genotype were analyzed per biological replicate.

### Image treatment

All images of plants grown *in vitro* were acquired by direct scanning plants within Petri dishes using an Epson V700 Photo scanner. For better contrast in some experiments, the Petri dish background was removed and replaced by black fields using Adobe Photoshop CS4 software.

## ACCESSION NUMBERS

Arabidopsis Genome Initiative (AGI) gene numbers: *bHLH39* (AT3G56980), *BOLA1* (AT1G55805), *BOLA4* (AT5G17560), *FER1* (AT5G01600), *FIT* (AT2G28160), *FRO2* (AT1G01580), *IRT1* (AT4G19690), *NAS4* (AT1G56430), *FRD3* (AT3G08040), *OPT3* (AT4G16370), *PP2AA3* (AT1G13320).

## AUTHOR CONTRIBUTIONS

Conceived and designed the experiments: MTB, ARE, BT, NB and FV. Performed the experiments: ARE, MTB, BT, NB, JB and FV. Contributed reagents/materials/analysis tools: DM, CD and FV. Analyzed the data: ARE, MTB, BT, JB, NB, CD and FV. Writing‐original draft: MBT, ARE and FV. Writing‐review and editing: CD and FV.

## CONFLICT OF INTEREST

The authors declare no conflicts of interest.

## Supporting information


**Data S1** Supporting experimental procedure.

**Figure S1.** *BOLA1* and *BOLA4* absolute transcript levels in Arabidopsis, from germinating seeds to senescent tissues. (A) Values recorded for all tissues. (B) All values given as histograms, in which *BOLA1* is represented by blue bars and *BOLA4* by orange bars. das, day after soaking; dpg, day post‐germination. (A, B) Data according to TraVA (http://travadb.org/, Klepikova et al., 2016).
**Figure S2.** *BOLA1* and *BOLA4* absolute transcript levels in Arabidopsis, from inflorescences to dry seeds. (A) Values recorded for all tissues. (B) All values given as histograms, in which *BOLA1* is represented by blue bars and *BOLA4* by orange bars. das, day after soaking; dpg, day post‐germination. (A, B) Data according to TraVA (http://travadb.org/, Klepikova et al., 2016).
**Figure S3.** Loss‐of‐function mutants in *BOLA* genes used in this study. A) Salk *bola1* and *bola4* mutants showing precise T‐DNA insertion sites. B) Nucleotide sequence analysis of the *bola4‐2* mutant allele. The nucleotide sequences of *BOLA4* exon 1 from wild‐type and the *bola4‐2* mutant were aligned using CLUSTALW 2.1, revealing a 5‐nucleotide deletion in *bola4‐2* after nucleotide 95 within the sgRNA1 target sequence (highlighted in bold blue). The ATG start codons are underlined. C) Comparison of the translated exon1 sequences from wild type and *bola4‐2*. Numbers to the right of the sequences indicate amino acid positions.
**Figure S4.** Validation of T‐DNA insertions in single *bola* mutants. (A) A schematic representation of the *BOLA1* and *BOLA4* genes, indicating the positions of T‐DNA insertions and CRISPR‐Cas9 cleavage sites. Gray boxes: untranslated regions, white boxes: exons, black lines: intergenic regions (including promoters) and introns. The regions of *BOLA* transcripts targeted by RT‐PCR experiments are depicted with black double arrows below the gene schematics, along with the expected sizes of cDNA amplicons expected in the control wild‐type (WT) line. (B) PCR genotyping was performed on genomic DNA isolated from the WT line and the four single *bola* mutants. The PCR was conducted using oligonucleotide pairs designed to amplify either the non‐mutated endogenous *BOLA1* or *BOLA4* regions or the T‐DNA borders flanking the *BOLA* genes. The names of the lines analyzed are listed at the top of the image, while the genes analyzed are indicated on the right. The size of the PCR products is shown on the left, with a 1 Kb ladder (L) used as a reference. The analysis confirmed the presence of T‐DNAs in the three Salk mutants (*bola1‐3, bola1‐4, bola4‐1*) and their absence in both the WT line and the CRISPR‐Cas9 *bola4‐2* mutant. For *bola1‐4*, PCR and sequencing identified a unique insertion of a two‐T‐DNA inverted repeat after the nucleotide +315 from the ATG start codon, with missing right borders. The pCAS9 plasmid DNA, which expresses the Cas9 nuclease, serves as a positive control to confirm the absence of Cas9 in the *bola4‐2* mutant. In this mutant, the DNA band at 1166 bp, marked with a red asterisk, indicates the presence of *BOLA4*. Sequencing of this fragment confirmed the presence of a 5‐nucleotide difference compared to the wild‐type BOLA4 (1171 bp).
**Figure S5.** Validation of knock‐out mutations in *bola* mutants. Gene expression analyses of *BOLA1* and *BOLA4* by RT‐PCR (9‐day‐old plantlets). RT‐PCR reactions were performed on cDNA stocks using oligonucleotides pairs designed to detect a 601 bp cDNA fragment for *BOLA1* and a 212 bp cDNA fragment for *BOLA4*, by comparison with a 69 bp cDNA fragment for the *PP2AA3* used as control. The analysis confirmed the loss‐of‐transcript in *bola1‐3, bola1‐4* and *bola4‐1* mutants compared to the wild type (WT), concluding that they are knock‐out mutants. As expected, a *BOLA4* transcript that carries a 5‐nucleotide deletion and early stop codon was detected in the *bola4‐2* line (marked by one red asterisk), but a lower extend compared to the WT and *bola1* mutants.
**Figure S6.** Verification of nucleotide sequence integrity for potential off‐target genes of the *BOLA4* sgRNA1 in the *bola4‐2* background. (A) List of off‐target genes predicted by Cas‐OFFinder. The first line (highlighted in gray) corresponds to *BOLA4* gene information. sgRNA1 target sequence used to generate the *bola4‐2* line was designed from a compilation of the records obtained by 4 different sgRNA design software on *BOLA4*: CHOPCHOP (https://chopchop.cbu.uib.no/, Labun et al., 2019), Cas‐designer (http://www.rgenome.net/cas‐designer/, Park et al., 2015), CRISPRscan (http://www.crisprscan.org/, Moreno‐Mateos et al., 2015), and WU‐Crispr (https://crisprdb.org/index.html, Chen & Wang, 2022). Nucleotide differences or absences between the *BOLA4* sgRNA1 target sequence and similar sequences in the identified off‐target genes are represented by red letters and dashes, respectively. A minimum of four mismatches or two bulges were required for Cas‐OFFinder settings to identify potential off‐target genes. No off‐targets were detected with a cumulative one‐base mismatch and one or two DNA bulges. (B) Sequencing of non‐target genes around the variant sequence identified as being able to be mistakenly recognized by the *BOLA4* guide RNA in the *bola4‐2* mutant genetic background. The sequence obtained for each off‐target gene was then compared with the *Arabidopsis thaliana* (taxid 3702) database on National Center for Biotechnology Information BlastN platform.
**Figure S7.** Comparison of growth between the wild‐type plant and the single *bola* mutants. (A) Plant growth on ½ MS medium. WT plants and single *bola* mutants were photographed 7 days after seed stratification and transfer to standard culture conditions. Scale bar = 1 cm. (B) Root growth measurements for all genotypes. Means within each condition with the same letter are not significantly different according to one‐way ANOVA with post hoc Tukey test, *P* < 0.01, *n* > 25. Error bars show means ± SD.
**Figure S8.** Morphological traits of *bola1‐4 bola4‐1* and *bola1‐3 bola4‐2* double mutants. (A) Both double mutants exhibited similar growth defects compared to the wild‐type plant (WT), including slow growth and a significant reduction in root development, resulting in dwarf plants. Light was not responsible for the dwarf phenotype, as this trait persisted even when roots were shielded from light. The scale bar is in centimeters. (B) Focus on the collar area of six‐day‐old plants: while the WT developed only a primary root, both double *bola* mutants have already initiated one or more lateral roots. Plants were imaged 6 days after the transfer of vernalized seeds to growth chamber.
**Figure S9.** Comparative analysis of root growth over time between Arabidopsis wild‐type plants and *bola* double mutants. A) Primary root length. B) Schematic representation of the wild type and *bola1‐4 bola4‐1* root systems.
**Figure S10.** BOLA1, like BOLA4, is located in plastids and in mitochondria in Arabidopsis. (A) Localization of BOLA1‐GFP and BOLA4‐GFP fusion proteins expressed from genomic copies of their gene under the control of their respective promoter (*proBOLA1:gBOLA1‐GFP* and *proBOLA4:gBOLA4‐GFP*) in wild type (WT) leaf protoplasts. (B) Localization of the BOLA1‐GFP fusion protein expressed either under the control of the *UBIQUITIN10* gene promoter (*proUBQ10:gBOLA1‐GFP*) in WT leaf protoplasts, or under the control of its own promoter (*proBOLA1:gBOLA1‐GFP*) in *bola4‐1* leaf protoplasts. (A, B) Arrows indicate mitochondria where the fluorescence of the BOLA1‐GFP and BOLA4‐GFP fusion proteins overlaps with that of the MitoTracker™ CMROS Orange fluorescence (MitoTracker, colored pink). Chloroplasts (ChloroP, colored red) are labeled by chlorophyll autofluorescence. The overlay of their respective fluorescence with that of the GFP fusion proteins is shown in two separate panels. Image acquisition on the confocal microscope 24 h after transfection is shown here with maximum Z‐axis projection. Note that the fluorescence of both BOLA1‐GFP and BOLA4‐GFP proteins, which naturally accumulate in chloroplasts (Couturier et al., 2014), is saturated in this compartment, because the laser power was increased to the standard detection of GFP fluorescence in mitochondria to record the presence of BOLA proteins in this specific compartment. All images are representative of five biological replicates, each based on approximately 20 cells displaying identical protein localization. Scale bar = 10 μm.
**Figure S11.** Subcellular localization of BOLA1 and BOLA4 in Arabidopsis tissues. Fluorescence patterns of BOLA1‐GFP and BOLA4‐GFP fusion proteins were analyzed in 7‐day‐old *bola4‐1* and WT cotyledons (A), hypocotyls (B), and roots (C, D). (A–D) BOLA1‐GFP was expressed under the *UBQ10* promoter (upper panels), whereas BOLA4‐GFP was expressed under its native promoter (*proBOLA4*; lower panels). Fluorescence of BOLA‐GFP fusion proteins (GFP) was analyzed in combination with autofluorescence of the chlorophyll (ChloroP) without (A) or with (B) propidium iodide (PI) in aerial tissues. The MitoTracker™ CMROS Orange (MitoT) stain did not penetrate into epidermal cells of aerial tissues, so the presence of BOLA in mitochondria was detected only through the GFP fluorescence in these tissues. Fluorescence of BOLA‐GFP fusion proteins in roots was analyzed in combination with PI (C) or MitoT (D). Images are representative of at least 10 plants from two independent transgenic lines. Scale bars = 50 μm.
**Figure S12.** The *Arabidopsis thaliana* BOLA1 and BOLA4 proteins accumulate throughout the whole‐plant body, whatever the iron concentration in the medium. (A, B) GUS staining patterns of BOLA1‐GUS and BOLA4‐GUS fusion proteins expressed under their own promoter. Seedlings were 7‐day‐old on sterile ½MS media with (A) 50 μM [Fe‐EDTA] (control condition) or with (B) 250 μM [Fe‐EDTA] (iron excess condition). Images are representative of at least 20 plants from three independent transgenic lines per BOLA‐GUS fusion protein analyzed.
**Figure S13.** Iron excess promotes *bola1‐4 bola4‐1* mutant growth independently of the Fe chelate used. (A) Effect of iron excess on 12‐day‐old WT and *bola1‐4 bola4‐1* plants grown vertically on ½ MS medium containing 1% sucrose, under sufficient (50 μM Fe‐EDTA or Fe‐EDDHA) or excess (250 μM Fe‐EDTA or 250 or 350 μM Fe‐EDDHA) iron conditions. Scale bars = 1 cm. B) Pictures of *bola1‐4 bola4‐1* plants growth on 50 μM, 250 μM or 350 μM Fe‐EDDHA. The arrows indicate the primary root. Scale bars = 2 mm. (C, D) Fresh weight (C) and primary root length (D) of plants described in (A). (C, D) Means within each condition with the same letter are not significantly different according to Kruskal–Wallis test with post hoc analysis (Conover, 1999), *P* < 0.05, with Holm corrections (*n* = 12). Error bars show means ±SD.
**Figure S14.** The double *bola1*‐*4 bola4‐1* mutation induces an iron deficiency response in *Arabidopsis thaliana*. Expression of iron marker genes was analyzed in roots of 12‐day‐old wild‐type (WT) and *bola1‐4 bola4‐1* mutant. A) Expression of genes involved in iron uptake (*FRO2*, *IRT1*), regulation of iron homeostasis (*bHLH39*, *FIT*) and iron storage (*FER1*). B) Expression of genes involved in nicotianamine transport (*NAS4*) and citrate efflux (*FRD3*) in the roots, and phloem‐specific iron (*OPT3*) transport in the shoots. (A, B) Transcript abundance was assessed by RT‐qPCR under control (50 μM Fe‐EDTA) or high (250 μM Fe‐EDTA) iron conditions, using the *PP2AA3* gene as control. Means within each condition with the same letter are not significantly different according to Kruskal–Wallis test with post hoc analysis (Conover, 1999), *P* < 0.01, with Holm corrections. Error bars show means ± SD. All analyses were done in 4 independent biological repeats, with each experiment done in triplicates. A biological repeat required 15 WT and 75 *bola1‐4 bola4‐1* seedlings.
**Figure S15.** Iron detection in three‐day‐old wild type and *bola1‐4 bola4‐1* double mutant. Plants were stained using the Perls/DAB method. Scale bars = 0.2 mm.
**Figure S16.** The double *bola1‐4 bola4‐1* mutation alters iron partitioning in Arabidopsis roots. (A) Whole‐plant Perls staining showing Fe distribution in seven‐day‐old WT and *bola1‐4 bola4‐1* double mutant seedlings grown under sufficient (50 μM Fe‐EDTA) or iron excess (250 μM Fe‐EDTA) conditions. Right panels (250 μM Fe) show that Fe excess partially rescues Fe distribution along the mutant root, resembling that of the WT root. Scale bars = 1 cm. (B) Focus on Perls staining of seven‐day‐old wild‐type (a‐d, i‐l) and *bola1‐4 bola4‐1* (e–h, m–p) primary roots under sufficient (a–h) or excess (i–p) Fe‐EDTA. (a, e, i, m) Views from the bottom of the hypocotyl to the upper part of the primary root, with a focus on the collar area (b, f, j, n). (c, g, k, o) Maturation zones. (d, h, l, p) Root tips, with insets (l', p') highlighting the meristematic and elongations zones. Scale bars = 0.2 mm.
**Figure S17.** Reciprocal grafting experiments between wild‐type plants and the *bola1‐4 bola4‐1* double mutant. (A) *BOLA1* and *BOLA4* gene expression in grafted plants shown in (B) was analyzed by RT‐PCR on cDNAs obtained from shoot (S) and root (R) transcripts of the different genotypes. Oligonucleotide pairs were designed to detect a 217 bp cDNA fragment of *BOLA1*, a 212 bp cDNA fragment of *BOLA4*, and a 69 bp cDNA fragment of *PP2AA3* used as control (Table S2). (B) Picture of the grafted plants 5 weeks post‐grafting. Shoot scion (S) and rootstock (R) genotypes (green letters for WT, orange letters for *bola1‐4 bola4‐1*) are indicated above and below the lines, respectively. WT/WT and *bola1‐4 bola4‐1/bola1‐4 bola4‐1* grafted plants were used as positive and negative controls, respectively. Scale bar = 1 cm.
**Figure S18.** Photosynthetic activity in the *bola1‐4 bola4‐1* double mutant. (A) Pigments content measured wild type and *bola1‐4 bola4‐1* leaves. Means with the same letter are not significantly different according to Kruskal–Wallis test with post hoc analysis (Conover, 1999), *P* < 0.05, with Bonferroni corrections. Error bars show means ± SD. *n* = 8 independent biological replicates, each requiring 15 WT and 22 *bola1‐4 bola4‐1* areal parts. B) Photosynthetic potential of the *bola1‐4 bola4‐1* mutant compared to the wild‐type plant. The maximum quantum yield (QY max) corresponds to the maximum photon absorption capacity by Photosystem II. Means are not significantly different according to a t‐test (*P* < 0.05). Error bars show means ± SD. *n* = 2 independent biological replicates, each performed on 35 seedlings per genotype. (C) False‐color images generated by the Fluorcam7 Software (Photon System Instrument) to obtain QY max values under actinic light (a, c), and plants used for QY max measurement under white light (b, d). (A, B, C) All experiments were done using 10‐day‐old seedlings grown horizontally on ½ MS media with 1% sucrose.
**Figure S19.** Immunoblot analysis of the abundance of the mitochondrial GRXS15 and SDH2, and the cytosolic ACO1 in the WT and *bola1‐4 bola4‐1* plants. All proteins analyzed were obtained from 15‐day‐old plants. (A) Mitochondrial proteins were obtained from roots and extracted according to Havird et al. (2019). (B) Total proteins were extracted from rosette leaves using a RIPA lysis buffer. (A, B) Proteins (40 μg in 1X Laemmli buffer) were separated on 15% (GRXS15) or 10% (SDH2, ACO1, Rubisco) SDS‐PAGE before transferring to PVDF membranes and revealed by cheluminescence using Goat anti‐Rabbit IgG (H&L), HRP conjugated antibodies (Agrisera). The polyclonal antibody against Arabidopsis GRXS15 was obtained from N. Rouhier (IAM Nancy, France). Antibodies against ACO1, Rubisco and SDH2‐1/2–2 were obtained from Agrisera and used according to the furnisher's recommendation. A PageRuler Prestained Protein Ladder (ThermoFisher Scientific) was used as the molecular mass marker in gels. The Precision Plus Protein™ WesternC™ Blotting Standards and StrepTactin‐HRP Conjugate (BioRad) were used to reveal protein markers on membranes post‐transfer. Commassie staining of the proteins in gels are shown as loading controls.

**Table S1.** Primers used for PCR genotyping.
**Table S2.** Primers used for gene expression analyses.
**Table S3.** Primers used for PCR diagnostics on transgenic plants.
**Table S4.** Primers used for cloning *BOLA1* and *BOLA* sequences.
**Table S5.** Primers used to amplify and sequence *BOLA4* sgRNA1 off‐targets.

## Data Availability

All data generated and/or analyzed during the current study are available from the corresponding author upon request.
