# Supplementary material for: Arabidopsis thaliana BOLA proteins, new actors in plant growth and iron homeostasis
Source: Plant J. 2026 Nov 4;128(3):e71196. doi: 10.1111/tpj.71196 (PMC13736203; doi:10.1111/tpj.71196)
Supplement: Supplementary file 1 [file TPJ-128-0-s001.pdf]

Supporting Tables for for

Arabidopsis thaliana BOLA proteins, new actors in plant growth and iron homeostasis

Maël TAUPIN-BROGGINI[1#], Anaïs RODRIGUEZ-EUZET[1#], Brigitte TOURAINE[1], Nathalie BERGER[1], Julie BALS[1], Daniel MARINO[2], Christian DUBOS[1], Florence VIGNOLS[1,*]

[1] Institute for Plant Sciences of Montpellier, Univ Montpellier, CNRS, INRAE, Institut Agro, Montpellier, France

[2] Department of Plant Biology and Ecology, University of the Basque Country, Bilbao, Spain

[#] These authors equally contributed to the work (alphabetical order)

Suuporting tables available for this article:

Table S1. Primers used for PCR genotyping.

Table S2. Primers used for gene expression analyses.

Table S3. Primers used for PCR diagnostics on transgenic plants.

Table S4. Primers used for cloning *BOLA1* and *BOLA* sequences.

Table S5. Primers used to amplify and sequence *BOLA4* sgRNA1 off-targets.

Supplemental tables:

Table S1. Primers used for PCR genotyping.

Genes	Forward primer (5'-3')	Reverse primer (5'-3')	Reference
BOLA1-30F/31R	TTGGATGAGCAGCTTAGAACC	TGTTCCAACACACAATTCCAG	This study
BOLA4-34F/35R	TGGTGAACATGTTATCAAATCAAA	TCATCATTGGAAGACAAAAATGAG	This study
CAS9	GCAACTCCCGCTTCGCTTGGAT	TTCTGCGTGGTCTGATTCTCCCGG	Baud et al. (2018)
LB1 (T-DNA)	CGGAACCACCATCAAACAG		This study

Table S2. Primers used for gene expression analyses.

Genes	Forward primer (5'-3')	Reverse primer (5'-3')	Reference
**RT-PCR**			
BOLA1(601)	TTCTCCGAGCGTCTTCATCT	TGTTCCAACACACAATTCCAG	This study
BOLA1(217)	GCCGTCTGAGTCTCCTTCTAAA	TGTTCCAACACACAATTCCAG	This study
BOLA4	AACGACATTGCCACAGAACA	AAAACGGCGCTTACACATCT	This study
PP2AA3	TAAGGTGGCCAAAATGATGC	GTTCTCCACAACCGCTTGGT	Reyt et al. (2015)
**qRT-PCR**			
BOLA1	TTCTCCGAGCGTCTTCATCT	TCCACTGGTTCGAGCTCTTT	This study
BOLA4	TGCAATCCATGGAGCTAAAG	CTCAAAAGCCGATGATACCA	This study
bHLH39	GACGGTTTCTCGAAGCTTG	GGTGGCTGCTTAACGTAACAT	Hong et al. (2013)
FIT	CAGTCACAAGCGAAGAAACTCA	CTTGTAAAGAGATGGAGCAACACC	Yuan et al. (2018)
FER1	TCGTTGAGAGTGAATTTCTGG	ACCCCAACATTGGTCATCTG	Tissot et al. (2019)
FRO2	AGTACGCCACAAGAATCGCT	CCACACTCGAACCTTCCACA	Cui et al. (2018)
FRD3	GGGATTGGCTTCTTCACCG	CCGATTCTTCGAAACACTTATTGAT	Charlier et al. (2015)
IRT1	CGGTTGGACTTCTAAATGC	CGATAATCGACATTCCACCG	Reyt et al. (2015)
NAS4	GGCTTCGACGTTGTGTTCTT	AGCAAAGCACCAGGAGACAT	Tissot et al. (2019)
OPT3	ATGTGGCATTGTTCAACGGCAGGGACA	GAAGCTGCACTGACTCCTTCCACAC	Chia et al. (2023)
PP2AA3	TAAGGTGGCCAAAATGATGC	GTTCTCCACAACCGCTTGGT	Reyt et al. (2015)

Table S3. Primers used for PCR diagnostics on transgenic plants.

Transgenic lines	Forward primer (5'-3')	Reverse primer (5'-3')	Reference
proBOLA1:gBOLA1	CTCGATGCACAGACAAAAACTG	GAAGGGTCTTGCGAAGGATAG	This study
proBOLA4:gBOLA4	ACAGCCTTCAAAACTACTTTGC	GAAGGGTCTTGCGAAGGATAG	This study
proBOLA1:gBOLA1-uidA	CTCGATGCACAGACAAAAACTG	CTGATACCAGACGTTGCCC	This study
proBOLA4:gBOLA4-uidA	ACAGCCTTCAAAACTACTTTGC	CTGATACCAGACGTTGCCC	This study
proBOLA1:gBOLA1-GFP	CTCGATGCACAGACAAAAACTG	CGGTGGTGCAGATGAACTTCAG	This study
proBOLA4:gBOLA4-GFP	ACAGCCTTCAAAACTACTTTGC	CGGTGGTGCAGATGAACTTCAG	This study
pUBQ10:gBOLA1-GFP	CTCCGAGCGTCTTCATCTCC	CGGTGGTGCAGATGAACTTCAG	This study

Table S4. Primers used for cloning *BOLA1* and *BOLA* sequences. *GsF* and *GsR* refer to sequences placed upstream of the forward *(F)* and reverse *(R)* primers for recombination of DNA fragments in the pDONR™207 vector. Additional bases to maintain open reading frames between BOLA and reporter proteins are underlined.

Constructs	Forward primer (5'-3')	Reverse primer (5'-3')
proBOLA1:gBOLA1	***GsF*** + AATCCACCACGACTCTCACC	***GsR*** + CTCCGAGAGCTCCCTCATC
proBOLA1:gBOLA1-uidA	***GsF*** + AATCCACCACGACTCTCACC	***GsR*** + CGTCTTTAGAAGGAGACTCAGAC
proBOLA1:gBOLA1-GFP	***GsF*** + AATCCACCACGACTCTCACC	***GsR*** + CGTCTTTAGAAGGAGACTCAGAC
proUBQ10:gBOLA1-GFP	***GsF*** +ATGTTTTCATCTTCGATTAGG	***GsR*** + CGTCTTTAGAAGGAGACTCAGAC
proBOLA4:gBOLA4	***GsF*** + CATTCTTCAATGGCTGGACAAG	***GsR*** + GAATTGATACGTTTCCATGCATAC
proBOLA4:gBOLA4-uidA	***GsF*** + CATTCTTCAATGGCTGGACAAG	***GsR*** + CAACTTCAGAAGGAGTCTTTGTTGTC
proBOLA4:gBOLA4-GFP	***GsF*** + CATTCTTCAATGGCTGGACAAG	***GsR*** + CAACTTCAGAAGGAGTCTTTGTTGTC

Gateway stretch	Forward primer (5'-3')	Reverse primer (5'-3')
GsF	*GGGGACAAGTTTGTACAAAAAAGCAGGCT*	
GsR		*GGGGACCACTTTGTACAAGAAAGCTGGGT*

Table S5. Primers used to amplify and sequence *BOLA4* sgRNA1 off-targets.

Targets	Forward primer (5'-3')	Reverse primer (5'-3')	Locus
BOLA4	TGGTGAACATGTTATCAAATCAAA	TCATCATTGGAAGACAAAAATGAG	AT5G17560
Off-target gene 1	ACCGTGGTATTTCACCTTCTTG	AGAGTATTTGGATGGACGAGGA	AT5G14950
Off-target gene 2	GATTGCGAAAACAGCCTCTC	GGAAATCGTCAAACCAGCTT	AT1G23180
Off-target gene 3	AGTAACGATATCGCCAACGACT	CATAGAGTGTCTGCCACCACAT	AT1G75880
Off-target gene 4	AACGAAAATGGATGCCAGAC	TGGTGGTTTGTGTTCCTCAA	AT2G11120
Off-target gene 5	TGCACCAAACTGGTTCTGTC	CACAAATGCTCCAAAACACG	AT2G28540
Off-target gene 6	TGACGCGATTCATCTTACTCAT	ACATGAGAGGAGAACTTGGGAA	AT2G37700
Off-target gene 7	ACAATCTTACAATTCGAGGCGT	TGAGGCTTTTTGAGGAGAGAAC	AT2G44980
Off-target gene 8	CACACAAATACCAAAAAGGCAA	GGAATCAGATCTTCATTTTCCG	AT4G23790
Off-target gene 9	TGATGAGCAAGACGTAGAAGGA	CCAAAAGTCAACGGTCAACATA	AT4G34950
Off-target gene 10	GATGCAGCTGTTATGAGGTCAG	CCAAACACTCTGGCTTCTCTCT	AT3G03740
Off-target gene 11	CCTGTTGATGTAGATGTCGGAA	TATCTGAGAACAGGGCAACTCA	AT3G10750
Off-target gene 12	CCATTGAAGCGTTAAGTGATGA	ACCTCCACAACCTTGTCACTCT	AT3G47890
Off-target gene 13	AATAAGCAAATGCAAAAGAGGC	GCAATTGATGCTGTGTCATCTT	AT3G60160
Off-target gene 14	TTTCAACCCGCCTAATGAAG	ATTCTTGTGCGCAACAGTCA	AT3G43563

References

Baud S, Mendoza MS, To A, Harscoët E, Lepiniec L, Dubreucq B (2015) WRINKLED1 specifies the regulatory action of LEAFY COTYLEDON2 towards fatty acid metabolism during seed maturation in Arabidopsis. Plant J **50**: 825-38

Charlier JB, Polese C, Nouet C, Carnol M, Bosman B, Krämer U, Motte P, Hanikenne M (2015) Zinc triggers a complex transcriptional and post-transcriptional regulation of the metal homeostasis gene *FRD3* in *Arabidopsis* relatives, J. Exp Bot **66**: 3865–3878

Chia JC, Yan J, Rahmati Ishka M, Faulkner MM, Simons E, Huang R, Smieska L, Woll A, Tappero R, Kiss A, Jiao C, Fei Z, Kochian LV, Walker E, Piñeros M, Vatamaniuk OK (2023) Loss of OPT3 function decreases phloem copper levels and impairs crosstalk between copper and iron homeostasis and shoot-to-root signaling in Arabidopsis thaliana. Plant Cell. **35**: 2157-2185

Cui Y, Chen CL, Cui M, Zhou WJ, Wu HL, Ling HQ (2018) Four IVa bHLH Transcription Factors Are Novel Interactors of FIT and Mediate JA Inhibition of Iron Uptake in *Arabidopsis*. Molec Plant **11**: 1166-1183

Hong S, Kim SA, Guerinot ML, McClung CR (2013) Reciprocal Interaction of the Circadian Clock with the Iron Homeostasis Network in Arabidopsis. Plant Physiol **161**: 893–903

Montague T G, Cruz JM, Gagnon JA, Church GM, Valen E (2014) CHOPCHOP: a CRISPR/Cas9 and TALEN web tool for genome editing. Nucleic Acids Res **42**: W401-407

Moreno-Mateos MA, Vejnar CE, Beaudoin JD, Fernandez JP, Mis EK, Khokha MK, Giraldez AJ (2015) CRISPRscan: designing highly efficient sgRNAs for CRISPR-Cas9 targeting in vivo. Nat Methods **12**: 982-988

Park J, Bae S, Kim JS (2015) Cas-Designer: A web-based tool for choice of CRISPR-Cas9 target sites. Bioinformatics **31**: 4014-4016

Reyt G, Boudouf S, Boucherez J, Gaymard F, Briat JF (2015) Iron- and ferritin-dependent reactive oxygen species distribution: impact on Arabidopsis root system architecture. Molec Plant **8**: 439-53

Tissot N, Robe K, Gao F, Grant-Grant S, Boucherez J, Bellegarde F, Maghiaoui A, Marcelin R, Izquierdo E, Benhamed M, et al. (2019) Transcriptional integration of the responses to iron availability in Arabidopsis by the bHLH factor ILR3. New Phytol **223**: 1433–1446

Wang ZP, Xing HL, Dong L, Zhang HY, Han CY, Wang XC, Chen QJ (2015) Egg cell-specific promoter-controlled CRISPR/Cas9 efficiently generates homozygous mutants for multiple target genes in Arabidopsis in a single generation. Genome Biol. **16**: 144

Wong N, Liu W, Wang X (2015) WU-CRISPR: characteristics of functional guide RNAs for the CRISPR/Cas9 system. Genome Biology **16**: 1-8

Yuan Y, Wu H, Wang N, Li J, Zhao W, Du J, Wang D, Ling H-Q (2008) FIT interacts with AtbHLH38 and AtbHLH39 in regulating iron uptake gene expression for iron homeostasis in Arabidopsis. Cell Res **18**: 385–397
