# Supplementary material for: Arabidopsis thaliana BOLA proteins, new actors in plant growth and iron homeostasis
Source: Plant J. 2026 Nov 4;128(3):e71196. doi: 10.1111/tpj.71196 (PMC13736203; doi:10.1111/tpj.71196)
Supplement: Supplementary file 2 [file TPJ-128-0-s002.pdf]

Supporting experimental procedure for:

Arabidopsis thaliana BOLA proteins, new actors in plant growth and iron homeostasis

Maël TAUPIN-BROGGINI[1#], Anaïs RODRIGUEZ-EUZET[1#], Brigitte TOURAINE[1], Nathalie BERGER[1], Julie BALS[1], Daniel MARINO[2], Christian DUBOS[1], Florence VIGNOLS[1,*]

[1] Institute for Plant Sciences of Montpellier, Univ Montpellier, CNRS, INRAE, Institut Agro, Montpellier, France

[2] Department of Plant Biology and Ecology, University of the Basque Country, Bilbao, Spain

* These authors equally contributed to the work (alphabetical order)

Methods S1

Crispr-Cas9 gene editing of *BOLA4*

To edit the Col-0 genome at the *BOLA4* locus by the Crispr-Cas9 strategy, we searched for single guide RNAs (sgRNA) from the records obtained by 4 different sgRNA design softwares on *BOLA4* (Supplemental Fig. S3B and S6). Criteria for the choice of the best *bola4* sgRNAs were their proximity to the ATG start codon, their location within codons, the probability to generate early stop codons following the CAS9-induced DNA repair, the smallest number of potential off-target genes as determined by the Cas-OFFinder software (http://www.rgenome.net/cas-offinder/, Bae et al., 2014). Two sgRNAs (sgRNA1, AGGAGCTGAGAATGAAGCGA and sgRNA2, GTGGTATCATCGGCTTTTGA) corresponding to these criteria were selected and cloned into the pRM-Cas9 binary vector (Wang et al., 2015) to generate the *pRM-cr-BOLA4* construct. Several different homozygous mutant alleles were obtained following plant transformation, among which we selected a *cr-bola4* homozygote displaying a 5-nucleotide deletion in the sgRNA1 target sequence in the first *BOLA4* exon (Supplemental Fig. S3B-C). In order to ensure that the sgRNA1 did not target other genes, potential off-target genes were searched using Cas-OFFinder (Supplemental Fig. S6A) and systematically sequenced around putative predicted mutation sites (Table S5 and Supplementary Fig. S6B). No differences with the WT sequences were observed.

Mitochondria isolation

Mitochondria were isolated from roots according to Harvid et al. (2019) without the use of BSA, and resuspended in ACA buffer (750 mM aminocaproic acid, 50 mM Bis-Tris, pH 7.0) prior use.

Reference

Havird, J.C., Noe, G.R., Link, L., Torres, A, Logan, D., Sloan, D.B. and Chicco, A.J. (2019) Do angiosperms with highly divergent mitochondrial genomes have altered mitochondrial function? *Mitochondrion*. **49**, 12–11.
