# Supplementary material for: Arabidopsis thaliana BOLA proteins, new actors in plant growth and iron homeostasis
Source: Plant J. 2026 Nov 4;128(3):e71196. doi: 10.1111/tpj.71196 (PMC13736203; doi:10.1111/tpj.71196)
Supplement: Supplementary file 3 [file TPJ-128-0-s003.pdf]

Figure S1. *BOLA1* and *BOLA4* absolute transcript levels in Arabidopsis, from germinating seeds to senescent tissues. **A)** Values recorded for all tissues. **B)** All values given as histograms, in which *BOLA1* is represented by blue bars and *BOLA4* by orange bars. das, day after soaking; dpg, day post germination. **(A,B)** Data according to TraVA (Klepikova *et al.*, 2016; http://travadb.org/).

A

Tissues	*BOLA1*	*BOLA4*
Germinating seeds. 1 das	962	1875
Germinating seeds 2 das	562	971
Germinating seeds 3 das	331	823
Seedling root	320	673
Root without apex	314	640
Root apex	378	811
Seedling hypocotyl	361	946
Seedling cotyledons	866	2147
Seedling meristem	3688	3584
SAM at 7 dpg	921	1013
SAM at 8 dpg	871	1004
SAM at 9 dpg	842	878
Meristem at 10 dpg	612	709
Meristem at 11 dpg	1200	1057
Meristem at 12 dpg	572	665
Internode	355	790
Petiole of the young leaf	839	1242
Petiole. intermediate 1	524	1224
Petiole. intermediate 2	458	1082
Petiole of the mature leaf	280	879
Leaf blade. intermediate 1	748	1817
Leaf blade. intermediate 2	718	2332
Leaf blade. the mature leaf	254	1189
Leaf blade of the young leaf	732	1751
Leaf vein. intermediate 2	506	1626
Vein of the mature leaf	229	880
Whole mature leaf	319	1086
Senescent internode.	259	673
Petiole of the senescent leaf	247	932
Vein of the senescent leaf	229	971

B

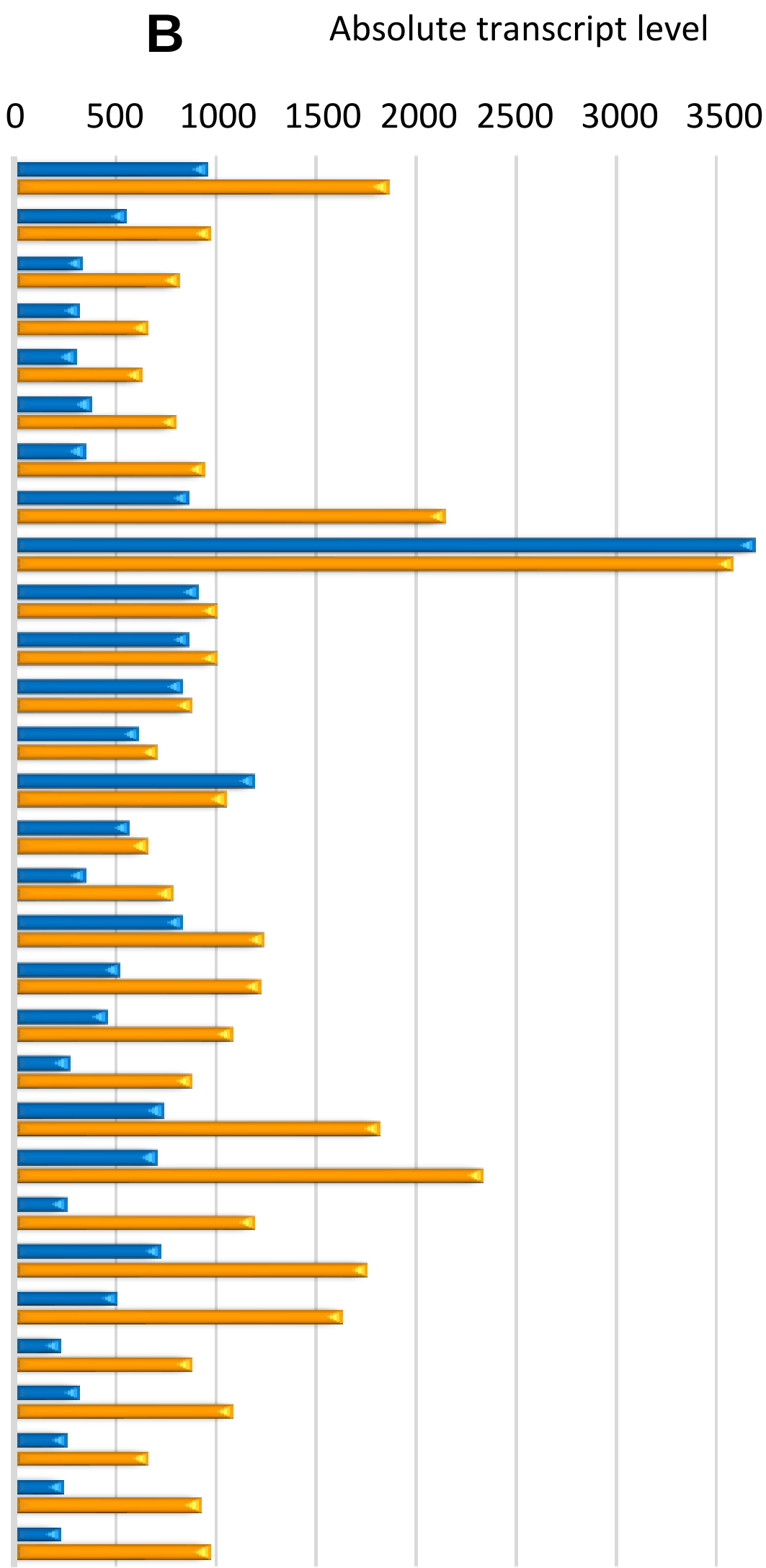

Figure S2. *BOLA1* and *BOLA4* absolute transcript levels in Arabidopsis, from inflorescences to dry seeds. **A)** Values recorded for all tissues. **B)** All values given as histograms, in which *BOLA1* is represented by blue bars and *BOLA4* by orange bars. das, day after soaking; dpg, day post germination. **(A,B)** Data according to TraVA (Klepikova *et al.*, 2016; http://travadb.org/).

A

Tissues	*BOLA1*	*BOLA4*
Inflorescence meristem at 13 dpg	583	584
Inflorescence meristem at 14 dpg	671	715
Inflorescence meristem at 15 dpg	655	778
Inflorescence meristem at 16 dpg	497	607
Axis of the inflorescence	512	921
Pedicel	531	1239
Carpels of the young flower	442	512
Carpels of the mature flower	403	668
Carpel of 6th and 7th flowers	424	617
Sepals of the young flower	611	1380
Sepals of the mature flower	315	1166
Petals of the mature flower	377	1102
Ovules from 6th and 7th flowers	470	634
Stigmatic tissue	375	1419
Stamen filaments. mature flower	669	1647
Anthers. young flower	784	1058
Anthers. mature flower (< opening)	245	723
Opened anthers	275	834
Flower 1	352	795
Flower 2	337	819
Flower 3	311	908
Flower 4	361	845
Flower 5	353	788
Flower 6-8	404	815
Flower 9-11	609	1029
Flower 12-14	371	652
Flower 15-18	447	756
Flower 19+	450	618
Pod of the silique 1	430	882
Pod of the senescent silique 1.	367	1430
Pod of the silique 3	445	964
Pod of the silique 5	462	1230
Pod of the silique 7	468	1163
Silique 2	687	951
Silique 4	568	1015
Silique 6	509	1075
Silique 8	423	979
Young seeds 1	411	860
Young seeds 2	387	767
Young seeds 3	434	709
Young seeds 4	503	747
Young seeds 5	597	939
Seeds 1	898	936
Seeds 3	749	970
Seeds 5	716	978
Seeds 7	502	974
Dry seeds	278	770

B

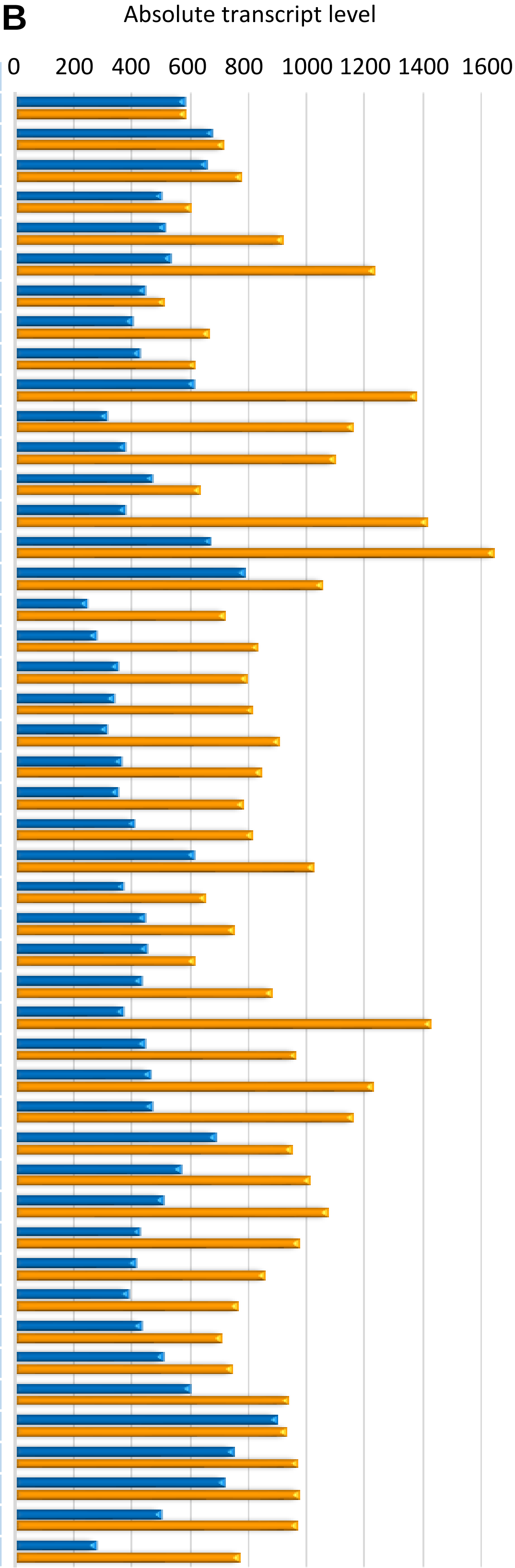

A

Mutant alleles	Origin	Genomic region	Mutation location in *BOLA* genes	Locus
bola1-1	SALK_022246	promoter	nuc^{-213} <T-DNA > nuc^{-212}	BOLA1 / AT1G55805.1
bola1-2	SALK_046137	nd	no match with At1g55805	-
bola1-3	SALK_059678	exon	Nuc^{+167} <T-DNA > nuc^{+168}	BOLA1 / AT1G55805.1
bola1-4	SALK_082155	exon	Nuc^{+315} <T-DNA > nuc^{+316}	BOLA1 / AT1G55805.1
bola1-5	SALK_017304	nd	no match with At1g55805	-
bola4-1	SALK_050846	exon1	nuc^{+33} <T-DNA > nuc^{+34}	BOLA4 / AT5G17560.1

B

Alignment of *BOLA4* coding region from Col-0 and *bola4-2*

```
Col-0      ATGGCGCAGACATTAATGGCGACAACTCGTCCGTACGTTCTTCTCTCTTCCACAAGAGCGAGTCTTTATCGCCTTTTTCA   80
bola4-2    ATGGCGCAGACATTAATGGCGACAACTCGTCCGTACGTTCTTCTCTCTTCCACAAGAGCGAGTCTTTATCGCCTTTTTCA
           ********************************************************************************

Col-0      ATCTTCAAAGAGACCCTTCGCTTCATTCTCAGCTCCTCTCGTGAGATTCACTAACTCGAGATGTGTAAGCGCCGTTTTGT   160
bola4-2    ATCTTCAAAGAGACC-----CTTCATTCTCAGCTCCTCTCGTGAGATTCACTAACTCGAGATGTGTAAGCGCCGTTTTGT
           ***************     ************************************************************

Col-0      CTCGTAAAGAGACTGCGCCGTCGTCGATTTACGGCAATAGAGTTTCTAGGGTTTCTGGGTTTGGTGCTTTGGACATTCGC   240
bola4-2    CTCGTAAAGAGACTGCGCCGTCGTCGATTTACGGCAATAGAGTTTCTAGGGTTTCTGGGTTTGGTGCTTTGGACATTCGC
           ********************************************************************************

Col-0      AGCCTTAATTTCAGTACTAAATCCTCTCAAATCAATGATGCTGGTTCTATTGATCAAACCCTTATGCAATCCATGGAGCT   320
bola4-2    AGCCTTAATTTCAGTACTAAATCCTCTCAAATCAATGATGCTGGTTCTATTGATCAAACCCTTATGCAATCCATGGAGCT
           ********************************************************************************
```

C

Translation of part of the wild-type *BOLA4* exon1

```
ATG GCG CAG ACA TTA ATG GCG ACA ACT CGT CCG TAC GTT CTT CTC TCT TCC ACA AGA GCG
 M   A   Q   T   L   M   A   T   T   R   P   Y   V   L   L   S   S   T   R   A     20
AGT CTT TAT CGC CTT TTT CAA TCT TCA AAG AGA CCC TTC GCT TCA TTC TCA GCT CCT CTC
 S   L   Y   R   L   F   Q   S   S   K   R   P   F   A   S   F   S   A   P   L     40
GTG AGA TTC ACT AAC TCG AGA TGT GTA AGC GCC GTT TTG TCT CGT AAA GAG ACT GCG CCG
 V   R   F   T   N   S   R   C   V   S   A   V   L   S   R   K   E   T   A   P     60
TCG TCG ATT TAC GGC AAT AGA GTT TCT AGG GTT TCT GGG TTT GGT GCT TTG GAC ATT CGC
 S   S   I   Y   G   N   R   V   S   R   V   S   G   F   G   A   L   D   I   R     80
```

BOLA4 exon1-intron1 sequence translation in *bola4-2*

```
ATG GCG CAG ACA TTA ATG GCG ACA ACT CGT CCG TAC GTT CTT CTC TCT TCC ACA AGA GCG
 M   A   Q   T   L   M   A   T   T   R   P   Y   V   L   L   S   S   T   R   A     20
AGT CTT TAT CGC CTT TTT CAA TCT TCA AAG AGA CCC TTC ATT CTC AGC TCC TCT CGT GAG
 S   L   Y   R   L   F   Q   S   S   K   R   P   F   I   L   S   S   S   R   E     40
ATT CAC TAA CTC GAG ATG TGT AAG CGC CGT TTT GTC TCG TAA AGA GAC TGC GCC GTC GTC
 I   H  Stop -   -   -   -   -   -   -   -   -   -   -   -   -   -   -   -   -     62
```

Figure S3. Loss-of-function mutants in *BOLA* genes used in this study. **A)** Salk *bola1* and *bola4* mutants showing precise T-DNA insertion sites. **B)** Nucleotide sequence analysis of the *bola4-2* mutant allele. The nucleotide sequences of *BOLA4* exon 1 from wild-type and the *bola4-2* mutant were aligned using CLUSTALW 2.1, revealing a 5-nucleotide deletion in *bola4-2* after nucleotide 95 within the sgRNA1 target sequence (highlighted in bold blue). The ATG start codons are underlined. **C)** Comparison of the translated exon1 sequences from wild type and *bola4-2*. Numbers to the right of the sequences indicate amino acid positions.

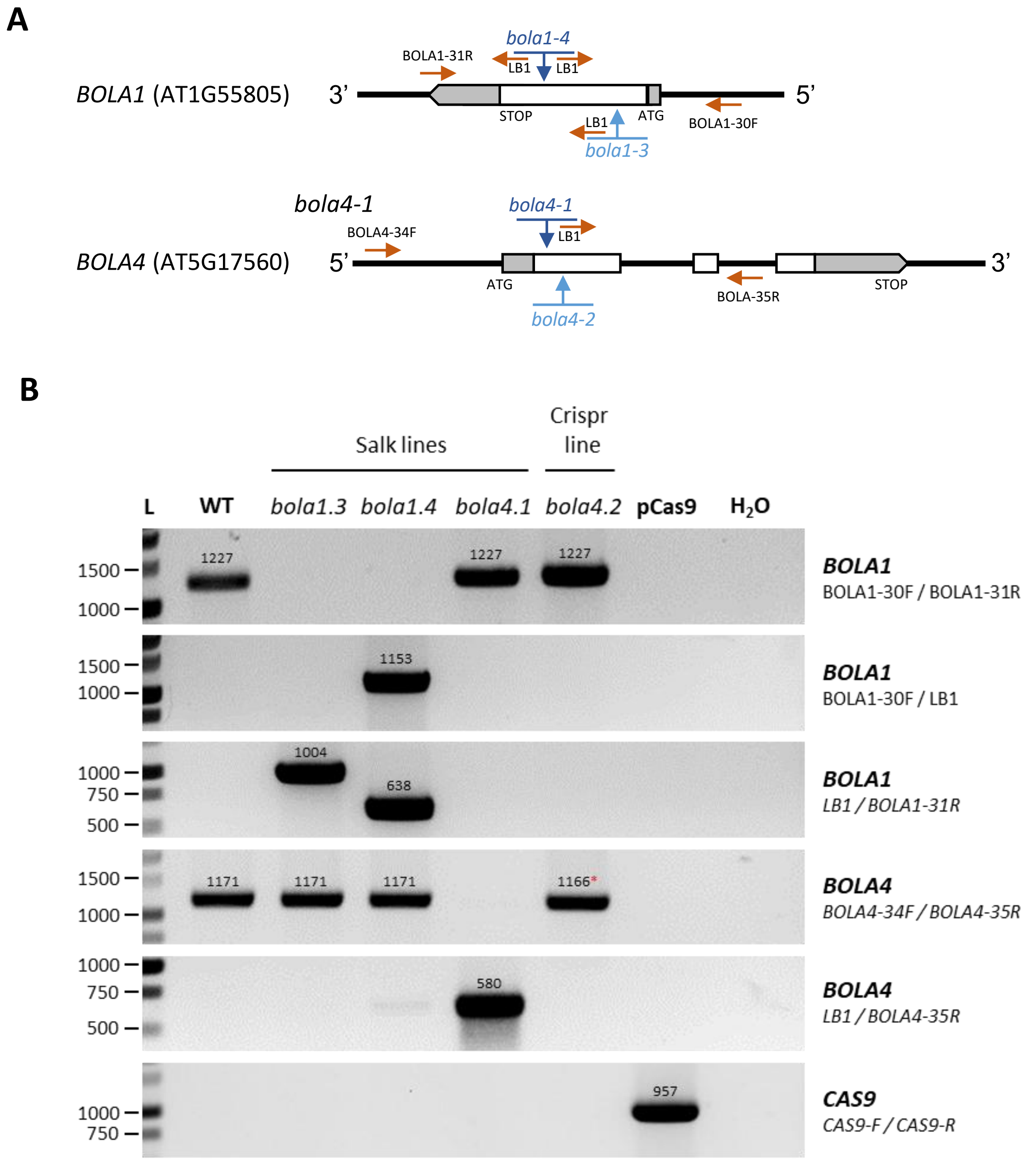

Figure S4. Validation of T-DNA insertions in single *bola* mutants. **A)** A schematic representation of the *BOLA1* and *BOLA4* genes, indicating the positions of T-DNA insertions and CRISPR-Cas9 cleavage sites. Grey boxes: untranslated regions, white boxes: exons, black lines: intergenic regions (including promoters) and introns. The regions of *BOLA* transcripts targeted by RT-PCR experiments are depicted with black double arrows below the gene schematics, along with the expected sizes of cDNA amplicons expected in the control wild-type (WT) line. **B)** PCR genotyping was performed on genomic DNA isolated from the WT line and the four single *bola* mutants. The PCR was conducted using oligonucleotide pairs designed to amplify either the non-mutated endogenous *BOLA1* or *BOLA4* regions or the T-DNA borders flanking the *BOLA* genes. The names of the lines analysed are listed at the top of the image, while the genes analysed are indicated on the right. The size of the PCR products is shown on the left, with a 1 Kb ladder (L) used as a reference. The analysis confirmed the presence of T-DNAs in the three Salk mutants (*bola1-3, bola1-4, bola4-1*) and their absence in both the WT line and the CRISPR-Cas9 *bola4-2* mutant. For *bola1-4*, PCR and sequencing identified a unique insertion of a two-T-DNA inverted repeat after the nucleotide +315 from the ATG start codon, with missing right borders. The pCAS9 plasmid DNA, which expresses the Cas9 nuclease, serves as a positive control to confirm the absence of Cas9 in the *bola4-2* mutant. In this mutant, the DNA band at 1166 bp, marked with a red asterisk, indicates the presence of *BOLA4*. Sequencing of this fragment confirmed the presence of a 5-nucleotide difference compared to the wild-type BOLA4 (1171 bp).

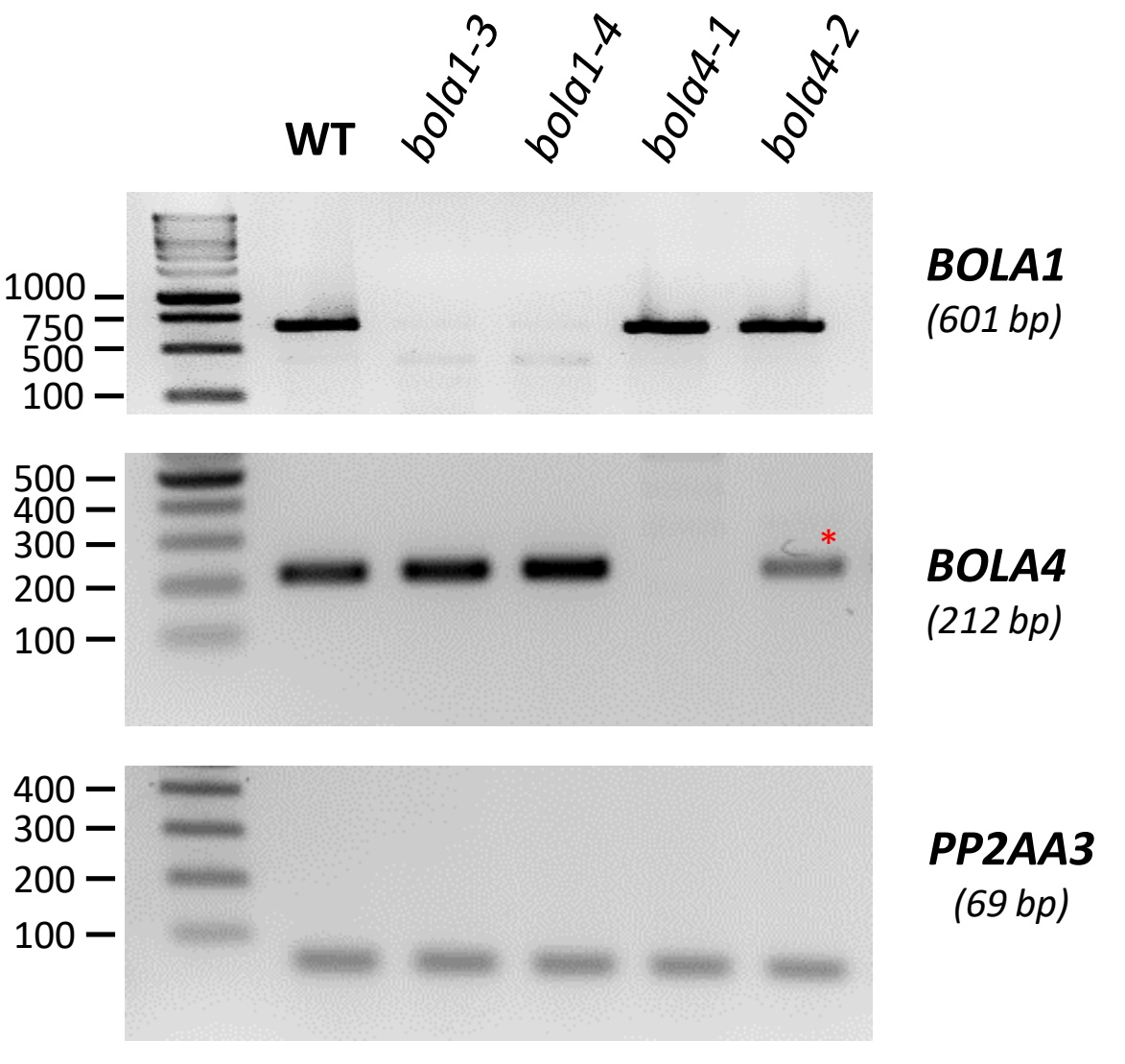

Figure S5. Validation of knock-out mutations in *bola* mutants. Gene expression analyses of *BOLA1* and *BOLA4* by RT-PCR (9-day-old plantlets). RT-PCR reactions were performed on cDNA stocks using oligonucleotides pairs designed to detect a 601 bp cDNA fragment for *BOLA1* and a 212 bp cDNA fragment for *BOLA4*, by comparison with a 69 bp cDNA fragment for the *PP2AA3* used as control. The analysis confirmed the loss-of-transcript in *bola1-3*, *bola1-4* and *bola4-1* mutants compared to the wild type (WT), concluding that they are knock-out mutants. As expected, a *BOLA4* transcript that carries a 5-nucleotide deletion and early stop codon was detected in the *bola4-2* line (marked by one red asterisk), but a lower extend compared to the WT and *bola1* mutants.

Figure S6. Verification of nucleotide sequence integrity for potential off-target genes of the *BOLA4* sgRNA1 in the *bola4-2* background. **A)** List of off-target genes predicted by Cas-OFFinder. The first line (highlighted in grey) corresponds to *BOLA4* gene information. sgRNA1 target sequence used to generate the *bola4-2* line was designed from a compilation of the records obtained by 4 different sgRNA design softwares on *BOLA4*: CHOPCHOP (http://chopchop.cbu.uib.no/, Montague et al., 2014), Cas-designer (http://www.rgenome.net/cas-designer/, Park et al., 2015), CRISPRscan (http://www.crisprscan.org/, Moreno-Mateos et al., 2015), and WU-Crispr (http://crispr.wustl.edu/, Wong et al., 2015). Nucleotide differences or absences between the *BOLA4* sgRNA1 target sequence and similar sequences in the identified off-target genes are represented by red letters and dashes, respectively. A minimum of four mismatches or two bulges were required for Cas-OFFinder settings to identify potential off-target genes. No off-targets were detected with a cumulative one-base mismatch and one or two DNA bulges. **B)** Sequencing of non-target genes around the variant sequence identified as being able to be mistakenly recognized by the *BOLA4* guide RNA in the *bola4-2* mutant genetic background. The sequence obtained for each off-target gene was then compared with the *Arabidopsis thaliana* (taxid 3702) database on National Center for Biotechnology Information BlastN platform.

A

Targets	Locus	Putative proteins	DNA strand	Mis-matches	Bulge	Variant sites for *BOLA4* sgRNA1 in off-target genes
Target gene	AT5G17560	BOLA4	-	0	0	AGGAGCTGAGAATGAAGCGAAGG
Off-target gene 1	AT5G14950	GMII, golgi alpha-mannosidase	+	4	0	AtGAGgTGAGAgaGAAGCGAAGG
Off-target gene 2	AT1G23180	ARM, armadillo repeat protein	+	4	0	AGaAcCTGAGAATGAAGCtgAGG
Off-target gene 3	AT1G75880	GGL12, lipase family member	-	4	0	AaGAGCgGtGAAaGAAGCGACGG
Off-target gene 4	AT2G11120	Putative Athila retroelement	-	4	0	AaGgGCTGAGAATGAAGaaAGGG
Off-target gene 5	AT2G28540	NOT4C, RNA binding	+	4	0	AGGtGCTGAaAATGAAtaGATGG
Off-target gene 6	AT2G37700	Fatty acid hydroxylase superfamily	+	4	0	AGGAcCTGAGAATGAAGgagAGG
Off-target gene 7	AT2G44980	ASG3, SNF2 domain-containing	+	4	0	AGaAGCaGAGAATGAAGCttTGG
Off-target gene 8	AT4G23790	Trochome Birefringence-Like 24	-	4	0	tGGAGtgGAGAATGAAGaGACGG
Off-target gene 9	AT4G34950	MFS1, Major facilitator superfamily	-	4	0	AGGAtCTGAGAtTGAAGtGgAGG
Off-target gene 10	AT3G03740	BPM4, MATH-BTB domain proteins	+	4	0	AGaAGCTcAGAcTGAAGtGACGG
Off-target gene 11	AT3G10750	FBD domain family	-	4	0	AGGAGCaGcGAtTGAAGgGACGG
Off-target gene 12	AT3G47890	Ubiquitin carboxyl-term hydrolase	-	4	0	AaGAatTGAGAATGAAGCtAAGG
Off-target gene 13	AT3G60160	ABCC9, MRP subfamily	-	4	0	AGGAGCTcAtAAcGAAGCGcTGG
Off-target gene 14	AT3G43563	Transposable element	-	0	2	AGGAGCTGAGAATGAAG--AAGG

B

Off-target 1, AT5G14950

Sequence obtained with the Forward primer

GGAGGTCACATGGTAACGGTTTGGCTAAAGGAGCAAAGGAACCGTATTGTGGAACACGCACAGATATGTCTTGCG
GTTTCTTGGCAATGAATGTGTTTATGGGGTAGTTTAAGTGAGCACCT**ATGAGGTGAGAGAGAAGCGAAGG**GTTCC
TCGGGTTAGTGTTGGAAGCAGGGTCTGCTTGAGAAATGTTAGATTCCGCAAGAAGGTGAAATACCACGGT

BlastN result

Arabidopsis thaliana Golgi alpha-mannosidase II (GMII) mRNA, complete cds
Sequence ID: DQ029214.1Length: 3673Number of Matches: 1
Gene-associated gene details
Range 1: 3092 to 3311GenBankGraphics Next Match Previous Match

Alignment statistics for match #1

Score	Expect	Identities	Gaps	Strand
407 bits(220)	8e-112	220/220(100%)	0/220(0%)	Minus

```
Query  1     GGAGGTCACATGGTAACGGTTTGGCTAAAGGAGCAAAGGAACCGTATTGTGGAACACGCA  60
             ||||||||||||||||||||||||||||||||||||||||||||||||||||||||||||
Sbjct  3311  GGAGGTCACATGGTAACGGTTTGGCTAAAGGAGCAAAGGAACCGTATTGTGGAACACGCA  3252
```

```
Query  61    CAGATATGTCTTGCGGTTTCTTGGCAATGAATGTGTTTATGGGGTAGTTTAAGTGAGCAC  120
             ||||||||||||||||||||||||||||||||||||||||||||||||||||||||||||
Sbjct  3251  CAGATATGTCTTGCGGTTTCTTGGCAATGAATGTGTTTATGGGGTAGTTTAAGTGAGCAC  3192

Query  121   CTATGAGGTGAGAGAGAAGCGAAGGGTTCCTCGGGTTAGTGTTGGAAGCAGGGTCTGCTT  180
             ||||||||||||||||||||||||||||||||||||||||||||||||||||||||||||
Sbjct  3191  CTATGAGGTGAGAGAGAAGCGAAGGGTTCCTCGGGTTAGTGTTGGAAGCAGGGTCTGCTT  3132

Query  181   GAGAAATGTTAGATTCCGCAAGAAGGTGAAATACCACGGT  220
             ||||||||||||||||||||||||||||||||||||||||
Sbjct  3131  GAGAAATGTTAGATTCCGCAAGAAGGTGAAATACCACGGT  3092
```

Off-target 2, AT1G23180

Sequence obtained with the Reverse primer

CATCGAGTTTGGTATAATCAGTAATCCAAACATGGACACGATCATCTCCAAGGATATCACAACTGTTCTTGATCT
TGCTCTCCGGCAGAAAGTCCTGGAAGGTACAAAACTTATCTTTTGATATAACTCTGTTTGAATTTTATAACAGCA
TACAATACTTTTTATGGAAATGC**AGAACCTGAGAATGAAGCTGAGG**AGTTAGAGAAACATCTGCTTAAGCTAGAA
GAAGCTGGTTTGACGATTTCC

BlastN result

Arabidopsis thaliana ARM repeat superfamily protein (AT1G23180), mRNA
Sequence ID: NM_001332592.1Length: 3099Number of Matches: 1
Gene-associated gene details
Genome Data Viewer-aligned genomic context
Range 1: 2177 to 2422GenBankGraphics Next Match Previous Match

Alignment statistics for match #1

Score	Expect	Identities	Gaps	Strand
455 bits(246)	4e-126	246/246(100%)	0/246(0%)	Plus

```
Query  1     CATCGAGTTTGGTATAATCAGTAATCCAAACATGGACACGATCATCTCCAAGGATATCAC  60
             ||||||||||||||||||||||||||||||||||||||||||||||||||||||||||||
Sbjct  2177  CATCGAGTTTGGTATAATCAGTAATCCAAACATGGACACGATCATCTCCAAGGATATCAC  2236

Query  61    AACTGTTCTTGATCTTGCTCTCCGGCAGAAAGTCCTGGAAGGTACAAAACTTATCTTTTG  120
             ||||||||||||||||||||||||||||||||||||||||||||||||||||||||||||
Sbjct  2237  AACTGTTCTTGATCTTGCTCTCCGGCAGAAAGTCCTGGAAGGTACAAAACTTATCTTTTG  2296

Query  121   ATATAACTCTGTTTGAATTTTATAACAGCATACAATACTTTTTATGGAAATGCAGAACCT  180
             ||||||||||||||||||||||||||||||||||||||||||||||||||||||||||||
Sbjct  2297  ATATAACTCTGTTTGAATTTTATAACAGCATACAATACTTTTTATGGAAATGCAGAACCT  2356

Query  181   GAGAATGAAGCTGAGGAGTTAGAGAAACATCTGCTTAAGCTAGAAGAAGCTGGTTTGACG  240
             ||||||||||||||||||||||||||||||||||||||||||||||||||||||||||||
Sbjct  2357  GAGAATGAAGCTGAGGAGTTAGAGAAACATCTGCTTAAGCTAGAAGAAGCTGGTTTGACG  2416

Query  241   ATTTCC  246
             ||||||
Sbjct  2417  ATTTCC  2422
```

Off-target 3, AT1G75880

Sequence obtained with the Forward primer

TTATTCGTGACATTAGTTATTGCCTTATTACCAACCTTACAAGTTACAATTGTAAATTGTCTTTACCTGAGCAAA
AGAACGAGCATTGTCGGCCAT**AAGAGCGGTGAAAGAAGCGACGG**TGTAGTGTAGCCGGACCGGGGGGAGAGTAAA
GAAGTCGTTGGCGATATCGTTACT

BlastN result

Arabidopsis thaliana clone RAFL15-09-P20 (R20737) putative family II extracellular lipase 1 (EXL1) (At1g75880) mRNA, complete cds
Sequence ID: BT004215.1Length: 1402Number of Matches: 1
Gene-associated gene details
Range 1: 682 to 789GenBankGraphics Next Match Previous Match

Alignment statistics for match #1

Score	Expect	Identities	Gaps	Strand
200 bits(108)	1e-49	108/108(100%)	0/108(0%)	Minus

```
Query  67   CTGAGCAAAAGAACGAGCATTGTCGGCCATAAGAGCGGTGAAAGAAGCGACGGTGTAGTG  126
            ||||||||||||||||||||||||||||||||||||||||||||||||||||||||||||
Sbjct  789  CTGAGCAAAAGAACGAGCATTGTCGGCCATAAGAGCGGTGAAAGAAGCGACGGTGTAGTG  730
```

```
Query  127   TAGCCGGACCGGGGGGAGAGTAAAGAAGTCGTTGGCGATATCGTTACT  174
             ||||||||||||||||||||||||||||||||||||||||||||||||
Sbjct  729   TAGCCGGACCGGGGGGAGAGTAAAGAAGTCGTTGGCGATATCGTTACT  682
```

Off-target 4, AT2G11120

Sequence obtained with the Reverse primer

AAGAGCTCACAACCATCACAGTGAGGTGTAACATCGATTTCAACCCTCCTAATGAGGCACTTTTCTTCATGGATGGGGCACCCCCGACAAGAGAAGCTCCGAACAATGAAGA**AAGGGCTGAGAATGAAGAAAGGG**CTGGGAATTAAGAAGTGGAGGAGATGGATTGGGAGAACTACAATGCGAGTAGGTTCCATTTTGAGGAACACAAACCACCA

BlastN result

Arabidopsis thaliana At2g11120 mRNA, partial sequence
Sequence ID: AY500291.1Length: 929Number of Matches: 1
Range 1: 75 to 289GenBankGraphics Next Match Previous Match

Alignment statistics for match #1

Score	Expect	Identities	Gaps	Strand
398 bits(215)	5e-109	215/215(100%)	0/215(0%)	Plus

```
Query  1     AAGAGCTCACAACCATCACAGTGAGGTGTAACATCGATTTCAACCCTCCTAATGAGGCAC  60
             ||||||||||||||||||||||||||||||||||||||||||||||||||||||||||||
Sbjct  75    AAGAGCTCACAACCATCACAGTGAGGTGTAACATCGATTTCAACCCTCCTAATGAGGCAC  134

Query  61    TTTTCTTCATGGATGGGGCACCCCCGACAAGAGAAGCTCCGAACAATGAAGAAAGGGCTG  120
             ||||||||||||||||||||||||||||||||||||||||||||||||||||||||||||
Sbjct  135   TTTTCTTCATGGATGGGGCACCCCCGACAAGAGAAGCTCCGAACAATGAAGAAAGGGCTG  194

Query  121   AGAATGAAGAAAGGGCTGGGAATTAAGAAGTGGAGGAGATGGATTGGGAGAACTACAATG  180
             ||||||||||||||||||||||||||||||||||||||||||||||||||||||||||||
Sbjct  195   AGAATGAAGAAAGGGCTGGGAATTAAGAAGTGGAGGAGATGGATTGGGAGAACTACAATG  254

Query  181   CGAGTAGGTTCCATTTTGAGGAACACAAACCACCA  215
             |||||||||||||||||||||||||||||||||||
Sbjct  255   CGAGTAGGTTCCATTTTGAGGAACACAAACCACCA  289
```

Off-target 5, AT2G28540

Sequence obtained with the Reverse primer

CTATCTATCTAGTGCCGGGCAATAATAATGTTTATAACATCTAGTGCGGCTTAGGCGTATTGGTATGTT**AGGTGCTGAAAATGAATAGATGG**CTTTATGTTCGGCATATTTCCACTGTTTTGAGTTAATGTGTTTCTTGGTTATGGAAATATTATACTTCCGGGATATTGACTTTGGCGTGTTTTGGAGCATTTGTG

BlastN result

Arabidopsis thaliana chromosome 2 clone T17D12 map mi54, complete sequence
Sequence ID: AC006587.5Length: 86579Number of Matches: 1
Range 1: 57467 to 57663GenBankGraphics Next Match Previous Match

Alignment statistics for match #1

Score	Expect	Identities	Gaps	Strand
364 bits(197)	4e-99	197/197(100%)	0/197(0%)	Plus

```
Query  1      CTATCTATCTAGTGCCGGGCAATAATAATGTTTATAACATCTAGTGCGGCTTAGGCGTAT  60
              ||||||||||||||||||||||||||||||||||||||||||||||||||||||||||||
Sbjct  57467  CTATCTATCTAGTGCCGGGCAATAATAATGTTTATAACATCTAGTGCGGCTTAGGCGTAT  57526

Query  61     TGGTATGTTAGGTGCTGAAAATGAATAGATGGCTTTATGTTCGGCATATTTCCACTGTTT  120
              ||||||||||||||||||||||||||||||||||||||||||||||||||||||||||||
Sbjct  57527  TGGTATGTTAGGTGCTGAAAATGAATAGATGGCTTTATGTTCGGCATATTTCCACTGTTT  57586

Query  121    TGAGTTAATGTGTTTCTTGGTTATGGAAATATTATACTTCCGGGATATTGACTTTGGCGT  180
              ||||||||||||||||||||||||||||||||||||||||||||||||||||||||||||
Sbjct  57587  TGAGTTAATGTGTTTCTTGGTTATGGAAATATTATACTTCCGGGATATTGACTTTGGCGT  57646

Query  181    GTTTTGGAGCATTTGTG  197
              |||||||||||||||||
Sbjct  57647  GTTTTGGAGCATTTGTG  57663
```

Off-target 6, AT2G37700

Sequence obtained with the Forward primer

ACGGTTTCTCTCGACGACAAAGGTTTGAAAAGAGA**AGGACCTGAGAATGAAGGAGAGG**ATAAGAGCGAAGGGCTT
CATGAGAAATAGGTAACACCGGGAAGAGAGCGGGTGAGAGGAGAGGGAAGCAAAGCCAAGCCGGAGATGGTAAAT
AGAGTCGAGTGATGTTAGATGAGTAAGATGAATCGCGTCA

BlastN result
Arabidopsis thaliana Fatty acid hydroxylase superfamily (AT2G37700), mRNA
Sequence ID: NM_001124992.2Length: 1789Number of Matches: 1
Gene-associated gene details
Genome Data Viewer-aligned genomic context
Range 1: 906 to 1094GenBankGraphics Next Match Previous Match

Alignment statistics for match #1

Score	Expect	Identities	Gaps	Strand
350 bits(189)	1e-94	189/189(100%)	0/189(0%)	Minus

```
Query  2     CGGTTTCTCTCGACGACAAAGGTTTGAAAAGAGAAGGACCTGAGAATGAAGGAGAGGATA  61
             ||||||||||||||||||||||||||||||||||||||||||||||||||||||||||||
Sbjct  1094  CGGTTTCTCTCGACGACAAAGGTTTGAAAAGAGAAGGACCTGAGAATGAAGGAGAGGATA  1035

Query  62    AGAGCGAAGGGCTTCATGAGAAATAGGTAACACCGGGAAGAGAGCGGGTGAGAGGAGAGG  121
             ||||||||||||||||||||||||||||||||||||||||||||||||||||||||||||
Sbjct  1034  AGAGCGAAGGGCTTCATGAGAAATAGGTAACACCGGGAAGAGAGCGGGTGAGAGGAGAGG  975

Query  122   GAAGCAAAGCCAAGCCGGAGATGGTAAATAGAGTCGAGTGATGTTAGATGAGTAAGATGA  181
             ||||||||||||||||||||||||||||||||||||||||||||||||||||||||||||
Sbjct  974   GAAGCAAAGCCAAGCCGGAGATGGTAAATAGAGTCGAGTGATGTTAGATGAGTAAGATGA  915

Query  182   ATCGCGTCA  190
             |||||||||
Sbjct  914   ATCGCGTCA  906
```

Off-target 7, AT2G37700

Sequence obtained with the Forward primer
GTGTTAAGCTCTTTGGGAGCACTTTTGTTTATAATTCCAAATATGATGAAACTTTAAAGAAAGAAAGTAATTAAG
TTCGTACCTGATTTCTG**AGAAGCAGAGAATGAAGCTTTGG**CTAAGCAACTTTCAAGGTCTGGAATTGAAATGCTA
CTACGTGGGACTTTACGCCTCGAATTGTAAGATTGT

BlastN result
Arabidopsis thaliana SNF2 domain-containing protein / helicase domain-containing protein (ASG3), mRNA
Sequence ID: NM_001337110.1Length: 2806Number of Matches: 1
Gene-associated gene details
Genome Data Viewer-aligned genomic context
Range 1: 2393 to 2496GenBankGraphics Next Match Previous Match

Alignment statistics for match #1

Score	Expect	Identities	Gaps	Strand
193 bits(104)	2e-47	104/104(100%)	0/104(0%)	Minus

```
Query  83    CTGATTTCTGAGAAGCAGAGAATGAAGCTTTGGCTAAGCAACTTTCAAGGTCTGGAATTG  142
             ||||||||||||||||||||||||||||||||||||||||||||||||||||||||||||
Sbjct  2496  CTGATTTCTGAGAAGCAGAGAATGAAGCTTTGGCTAAGCAACTTTCAAGGTCTGGAATTG  2437

Query  143   AAATGCTACTACGTGGGACTTTACGCCTCGAATTGTAAGATTGT  186
             ||||||||||||||||||||||||||||||||||||||||||||
Sbjct  2436  AAATGCTACTACGTGGGACTTTACGCCTCGAATTGTAAGATTGT  2393
```

Off-target 8, AT4G23790

Sequence obtained with the Forward primer
GGCGGCAGAAACCGAGCTTCGAATTTTCCGGTGACCGACGCAAAGATTGGAGAAAACTCGC**TGGAGTGGAGAATG**
AAGAGACGGAACGCGAAAAAAGATATCAGAATAGCGGAGATGAGCTTGATGAGAAGCACATTCTTATGATTCATG
GAGATTGATGACAACTTCAACTTCATCTCAAGAAGAAGATGAAGATATGCAAACAGTTCAGTGAGTTGTTTTTGC
CTTTTTGGTATTTGTGTG

BlastN result
Arabidopsis thaliana TRICHOME BIREFRINGENCE-LIKE 24 (TBL24), mRNA
Sequence ID: NM_118510.4Length: 1781Number of Matches: 1

Gene-associated gene details
Genome Data Viewer-aligned genomic context
Range 1: 173 to 415GenBankGraphics Next Match Previous Match

Alignment statistics for match #1

Score	Expect	Identities	Gaps	Strand
449 bits(243)	7e-122	243/243(100%)	0/243(0%)	Minus

```
Query  1    GGCGGCAGAAACCGAGCTTCGAATTTTCCGGTGACCGACGCAAAGATTGGAGAAAACTCG  60
            ||||||||||||||||||||||||||||||||||||||||||||||||||||||||||||
Sbjct  415  GGCGGCAGAAACCGAGCTTCGAATTTTCCGGTGACCGACGCAAAGATTGGAGAAAACTCG  356

Query  61   CTGGAGTGGAGAATGAAGAGACGGAACGCGAAAAAAGATATCAGAATAGCGGAGATGAGC  120
            ||||||||||||||||||||||||||||||||||||||||||||||||||||||||||||
Sbjct  355  CTGGAGTGGAGAATGAAGAGACGGAACGCGAAAAAAGATATCAGAATAGCGGAGATGAGC  296

Query  121  TTGATGAGAAGCACATTCTTATGATTCATGGAGATTGATGACAACTTCAACTTCATCTCA  180
            ||||||||||||||||||||||||||||||||||||||||||||||||||||||||||||
Sbjct  295  TTGATGAGAAGCACATTCTTATGATTCATGGAGATTGATGACAACTTCAACTTCATCTCA  236

Query  181  AGAAGAAGATGAAGATATGCAAACAGTTCAGTGAGTTGTTTTTGCCTTTTTGGTATTTGT  240
            ||||||||||||||||||||||||||||||||||||||||||||||||||||||||||||
Sbjct  235  AGAAGAAGATGAAGATATGCAAACAGTTCAGTGAGTTGTTTTTGCCTTTTTGGTATTTGT  176

Query  241  GTG  243
            |||
Sbjct  175  GTG  173
```

Off-target 9, AT4G34950

Sequence obtained with the Reverse primer

CTTTACTAAGATC**AGGATCTGAGATTGAAGTGGAGG**AAACAATCGTAGGTGCTGCAGCGGCGGCGGATAACGAAT
TGCCACCGTCTCTTAAGCCGTTAAGTAACGAGGAGGAAGAGAATCACGGAACTATAGTGACGACGGAGAAGAAAA
GACCGGTTCTTGGAGAAGAACACACCATAATGGAAGCTATGTTGACCGTTGACTTTTGG

BlastN result

Arabidopsis thaliana At4g34950 mRNA, complete cds
Sequence ID: BT012649.1Length: 1236Number of Matches: 1
Range 1: 848 to 1056GenBankGraphics Next Match Previous Match

Alignment statistics for match #1

Score	Expect	Identities	Gaps	Strand
387 bits(209)	1e-105	209/209(100%)	0/209(0%)	Plus

```
Query  1     CTTTACTAAGATCAGGATCTGAGATTGAAGTGGAGGAAACAATCGTAGGTGCTGCAGCGG  60
             ||||||||||||||||||||||||||||||||||||||||||||||||||||||||||||
Sbjct  848   CTTTACTAAGATCAGGATCTGAGATTGAAGTGGAGGAAACAATCGTAGGTGCTGCAGCGG  907

Query  61    CGGCGGATAACGAATTGCCACCGTCTCTTAAGCCGTTAAGTAACGAGGAGGAAGAGAATC  120
             ||||||||||||||||||||||||||||||||||||||||||||||||||||||||||||
Sbjct  908   CGGCGGATAACGAATTGCCACCGTCTCTTAAGCCGTTAAGTAACGAGGAGGAAGAGAATC  967

Query  121   ACGGAACTATAGTGACGACGGAGAAGAAAAGACCGGTTCTTGGAGAAGAACACACCATAA  180
             ||||||||||||||||||||||||||||||||||||||||||||||||||||||||||||
Sbjct  968   ACGGAACTATAGTGACGACGGAGAAGAAAAGACCGGTTCTTGGAGAAGAACACACCATAA  1027

Query  181   TGGAAGCTATGTTGACCGTTGACTTTTGG  209
             |||||||||||||||||||||||||||||
Sbjct  1028  TGGAAGCTATGTTGACCGTTGACTTTTGG  1056
```

Off-target 10, AT3G03740

Sequence obtained with the Forward primer

ATCTCCCCAAGTCTGTGCTTGTCTTCCGTTTGTCTCAGCTCCACCGTCTGAGAATTGGCCCCATACGCTTCTCGT
CTTCCCTCCTCCTCCGCTTAACTCCTCTTCGCAACCCGCAACTGTTTTC**AGAAGCTCAGACTGAAGTGACGG**GCA
GTGTTCCCTTAGATAATCAAACCCATCTGACCTCATAACAGCTGCATC

BlastN result

Arabidopsis thaliana ecotype Col-0 At3g03740 gene, partial cds

Sequence ID: EU351264.1Length: 1011Number of Matches: 1
Range 1: 763 to 960GenBankGraphics Next Match Previous Match

Alignment statistics for match #1

Score	Expect	Identities	Gaps	Strand
366 bits(198)	1e-99	198/198(100%)	0/198(0%)	Minus

```
Query  1    ATCTCCCCAAGTCTGTGCTTGTCTTCCGTTTGTCTCAGCTCCACCGTCTGAGAATTGGCC  60
            ||||||||||||||||||||||||||||||||||||||||||||||||||||||||||||
Sbjct  960  ATCTCCCCAAGTCTGTGCTTGTCTTCCGTTTGTCTCAGCTCCACCGTCTGAGAATTGGCC  901

Query  61   CCATACGCTTCTCGTCTTCCCTCCTCCTCCGCTTAACTCCTCTTCGCAACCCGCAACTGT  120
            ||||||||||||||||||||||||||||||||||||||||||||||||||||||||||||
Sbjct  900  CCATACGCTTCTCGTCTTCCCTCCTCCTCCGCTTAACTCCTCTTCGCAACCCGCAACTGT  841

Query  121  TTTCAGAAGCTCAGACTGAAGTGACGGGCAGTGTTCCCTTAGATAATCAAACCCATCTGA  180
            ||||||||||||||||||||||||||||||||||||||||||||||||||||||||||||
Sbjct  840  TTTCAGAAGCTCAGACTGAAGTGACGGGCAGTGTTCCCTTAGATAATCAAACCCATCTGA  781

Query  181  CCTCATAACAGCTGCATC  198
            ||||||||||||||||||
Sbjct  780  CCTCATAACAGCTGCATC  763
```

Off-target 11, AT3G10750

Sequence obtained with the Forward primer

GCCTAACATGAGAATCTTGGTCTTTATACACCACATTGACA**AGGAGCAGCGATTGAAGGGACGG**GAGGCAAGCCG
CGGAAGGAACATCAACAAGAATCTTGCCCATAAGATTCAATTTCACAAGCGTTTTGCTGGTGTAAAGCCTCTTAC
TGGCAGGATCTGAGAGAAAACTAAGACTTAGGGTTAGCTCTCTAACGTACCGGTCAACTGCATTCGAGACCCACT
TTCCGACATCTACAT

BlastN result

Arabidopsis thaliana FBD domain family (AT3G10750), partial mRNA
Sequence ID: NM_111912.2Length: 1422Number of Matches: 1
Gene-associated gene details
Genome Data Viewer-aligned genomic context
Range 1: 169 to 415GenBankGraphics Next Match Previous Match

Alignment statistics for match #1

Score	Expect	Identities	Gaps	Strand
457 bits(247)	1e-126	247/247(100%)	0/247(0%)	Minus

```
Query  1    GCCTAACATGAGAATCTTGGTCTTTATACACCACATTGACAAGGAGCAGCGATTGAAGGG  60
            ||||||||||||||||||||||||||||||||||||||||||||||||||||||||||||
Sbjct  415  GCCTAACATGAGAATCTTGGTCTTTATACACCACATTGACAAGGAGCAGCGATTGAAGGG  356

Query  61   ACGGGAGGCAAGCCGCGGAAGGAACATCAACAAGAATCTTGCCCATAAGATTCAATTTCA  120
            ||||||||||||||||||||||||||||||||||||||||||||||||||||||||||||
Sbjct  355  ACGGGAGGCAAGCCGCGGAAGGAACATCAACAAGAATCTTGCCCATAAGATTCAATTTCA  296

Query  121  CAAGCGTTTTGCTGGTGTAAAGCCTCTTACTGGCAGGATCTGAGAGAAAACTAAGACTTA  180
            ||||||||||||||||||||||||||||||||||||||||||||||||||||||||||||
Sbjct  295  CAAGCGTTTTGCTGGTGTAAAGCCTCTTACTGGCAGGATCTGAGAGAAAACTAAGACTTA  236

Query  181  GGGTTAGCTCTCTAACGTACCGGTCAACTGCATTCGAGACCCACTTTCCGACATCTACAT  240
            ||||||||||||||||||||||||||||||||||||||||||||||||||||||||||||
Sbjct  235  GGGTTAGCTCTCTAACGTACCGGTCAACTGCATTCGAGACCCACTTTCCGACATCTACAT  176
```

Off-target 12, AT3G47890

Sequence obtained with the Forward primer

CAGACGTTGTAACGAGTTAGAGGAAGAGGAGAGGAAGCTTGCAAAAACTCTAGAGTATCAAAGAAGAATTGAGAA
TGAAGCTAAGGAGAAGCACATCGCCGAACAAAAAAAGAAATACAGCTGTTCAGATCCGATGAATGTGACAGAGGC
AGTGTATGATGATTGCATAGAAAATTTCTTTGATGATGCTGATTTACAAGAACAAGAAAAACCCATAAATCAGGT
AACAATAGAGTGACAAGGTTGTGGAGGT

BlastN result

Arabidopsis thaliana Ubiquitin carboxyl-terminal hydrolase-related protein (AT3G47890), mRNA
Sequence ID: NM_001339339.1Length: 5250Number of Matches: 1
Gene-associated gene details

Genome Data Viewer-aligned genomic context
Range 1: 3229 to 3378GenBankGraphics Next Match Previous Match

Alignment statistics for match #1

Score	Expect	Identities	Gaps	Strand
297 bits(150)	2e-79	150/150(100%)	0/150(0%)	Plus

```
Query  1     CAGACGTTGTAACGAGTTAGAGGAAGAGGAGAGGAAGCTTGCAAAAACTCTAGAGTATCA  60
             ||||||||||||||||||||||||||||||||||||||||||||||||||||||||||||
Sbjct  3229  CAGACGTTGTAACGAGTTAGAGGAAGAGGAGAGGAAGCTTGCAAAAACTCTAGAGTATCA  3288

Query  61    AAGAAGAATTGAGAATGAAGCTAAGGAGAAGCACATCGCCGAACAAAAAAAGAAATACAG  120
             ||||||||||||||||||||||||||||||||||||||||||||||||||||||||||||
Sbjct  3289  AAGAAGAATTGAGAATGAAGCTAAGGAGAAGCACATCGCCGAACAAAAAAAGAAATACAG  3348

Query  121   CTGTTCAGATCCGATGAATGTGACAGAGGC  150
             ||||||||||||||||||||||||||||||
Sbjct  3349  CTGTTCAGATCCGATGAATGTGACAGAGGC  3378
```

Off-target 13, AT3G47890

Sequence obtained with the Reverse primer

TTAACATTAGTGTGTGTACTTTGTATACTAGGTGATGCAAAACGGAAGAGTGATGCAAGCAGGCAAGTTTGAAGA
GCTTCTAAAGCAAAACATAGGTTTTGAAGTTCTTGT**AGGAGCTCATAACGAAGCGCTGG**ACTCAATTCTATCAAT
TGAAAAGTCAAGCAGAAACTTCAAAGAGGGATCAAAAGATGACACAGCATCAATTGC

BlastN result

Arabidopsis thaliana multidrug resistance-associated protein 9 (ABCC9), mRNA
Sequence ID: NM_001340029.1Length: 5232Number of Matches: 1
Gene-associated gene details
Genome Data Viewer-aligned genomic context
Range 1: 2843 to 3019GenBankGraphics Next Match Previous Match

Alignment statistics for match #1

Score	Expect	Identities	Gaps	Strand
351 bits(177)	2e-95	177/177(100%)	0/177(0%)	Plus

```
Query  31    GGTGATGCAAAACGGAAGAGTGATGCAAGCAGGCAAGTTTGAAGAGCTTCTAAAGCAAAA  90
             ||||||||||||||||||||||||||||||||||||||||||||||||||||||||||||
Sbjct  2843  GGTGATGCAAAACGGAAGAGTGATGCAAGCAGGCAAGTTTGAAGAGCTTCTAAAGCAAAA  2902

Query  91    CATAGGTTTTGAAGTTCTTGTAGGAGCTCATAACGAAGCGCTGGACTCAATTCTATCAAT  150
             ||||||||||||||||||||||||||||||||||||||||||||||||||||||||||||
Sbjct  2903  CATAGGTTTTGAAGTTCTTGTAGGAGCTCATAACGAAGCGCTGGACTCAATTCTATCAAT  2962

Query  151   TGAAAAGTCAAGCAGAAACTTCAAAGAGGGATCAAAAGATGACACAGCATCAATTGC  207
             |||||||||||||||||||||||||||||||||||||||||||||||||||||||||
Sbjct  2963  TGAAAAGTCAAGCAGAAACTTCAAAGAGGGATCAAAAGATGACACAGCATCAATTGC  3019
```

Off-target 14, AT3G43563

Sequence obtained with the Reverse primer

TGGAACGAGCACCCCCGACGAGAGAGGCTCCAGACAATGAAGAAAGGGTTGAGAGTGAAGA**AGGAGCTGAGAATG**
AAGAAGGAGATGAGAATGAAGAAGCAGAGGAGATGAATTGGGAGAACTACAATGCGAGTAGGTTCCATTTCAAGG
AACACAAGCCACCACCAAGAGTGAGTAAGAGTCTGACTGTTGCGCACAAGAAT

BlastN result

Arabidopsis thaliana DNA chromosome 3, BAC clone F22J12
Sequence ID: AL391734.1Length: 100231Number of Matches: 5
Range 1: 17337 to 17539GenBankGraphics Next Match Previous Match

Alignment statistics for match #1

Score	Expect	Identities	Gaps	Strand
417 bits(203)	3e-115	203/203(100%)	0/203(0%)	Minus

```
Query  22     TGGAACGAGCACCCCCGACGAGAGAGGCTCCAGACAATGAAGAAAGGGTTGAGAGTGAAG  81
              ||||||||||||||||||||||||||||||||||||||||||||||||||||||||||||
Sbjct  17539  TGGAACGAGCACCCCCGACGAGAGAGGCTCCAGACAATGAAGAAAGGGTTGAGAGTGAAG  17480
```

```
Query  82     AAGGAGCTGAGAATGAAGAAGGAGATGAGAATGAAGAAGCAGAGGAGATGAATTGGGAGA  141
              ||||||||||||||||||||||||||||||||||||||||||||||||||||||||||||
Sbjct  17479  AAGGAGCTGAGAATGAAGAAGGAGATGAGAATGAAGAAGCAGAGGAGATGAATTGGGAGA  17420

Query  142    ACTACAATGCGAGTAGGTTCCATTTCAAGGAACACAAGCCACCACCAAGAGTGAGTAAGA  201
              ||||||||||||||||||||||||||||||||||||||||||||||||||||||||||||
Sbjct  17419  ACTACAATGCGAGTAGGTTCCATTTCAAGGAACACAAGCCACCACCAAGAGTGAGTAAGA  17360

Query  202    GTCTGACTGTTGCGCACAAGAAT  224
              |||||||||||||||||||||||
Sbjct  17359  GTCTGACTGTTGCGCACAAGAAT  17337
```

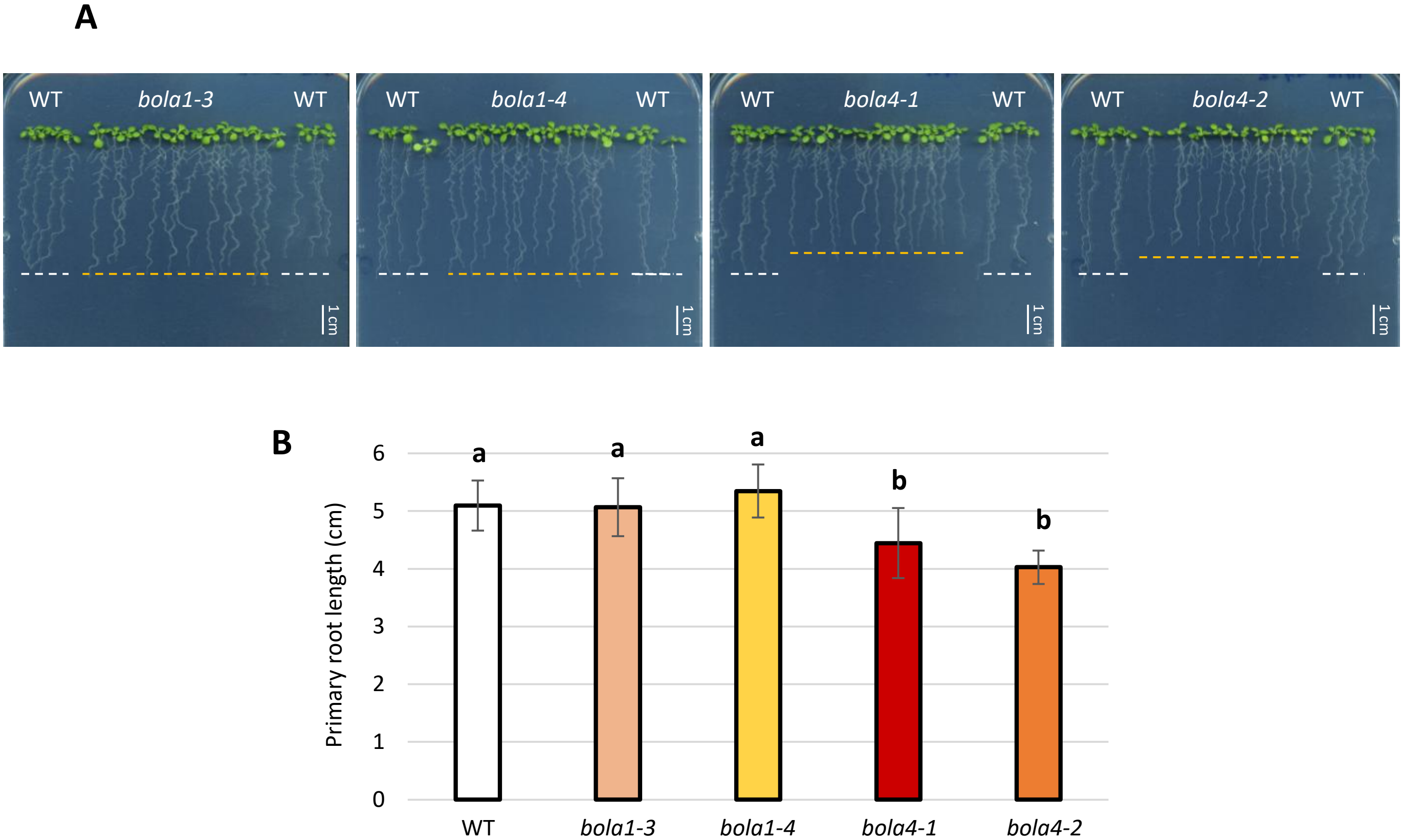

Figure S7. Comparison of growth between the wild-type plant and the single *bola* mutants. **A)** Plant growth on ½ MS medium. WT plants and single *bola* mutants were photographed 7 days after seed stratification and transfer to standard culture conditions. Scale bar = 1 cm. **B)** Root growth measurements for all genotypes. Means within each condition with the same letter are not significantly different according to one-way ANOVA with post hoc Tukey test, $p < 0.01$, $n > 25$. Error bars show means ± SD.

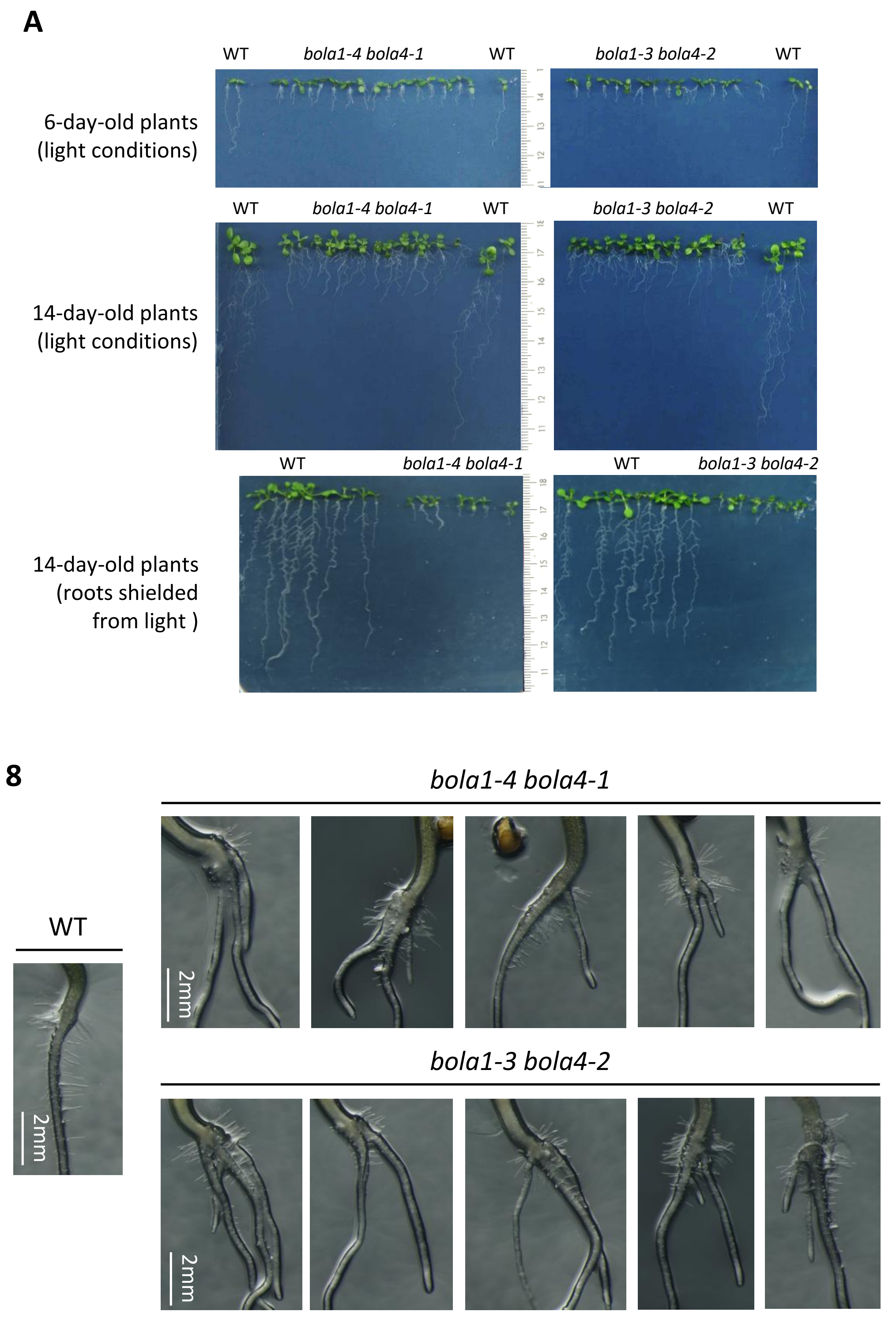

Figure S8. Morphological traits of *bola1-4 bola4-1* and *bola1-3 bola4-2* double mutants. **A)** Both double mutants exhibited similar growth defects compared to the wild-type plant (WT), including slow growth and a significant reduction in root development, resulting in dwarf plants. Light was not responsible for the dwarf phenotype, as this trait persisted even when roots were shielded from light. The scale bar is in centimeters. **B)** Focus on the collar area of six-day-old plants: while the WT developed only a primary root, both double *bola* mutants have already initiated one or more lateral roots. Plants were imaged six days after the transfer of vernalized seeds to growth chamber.

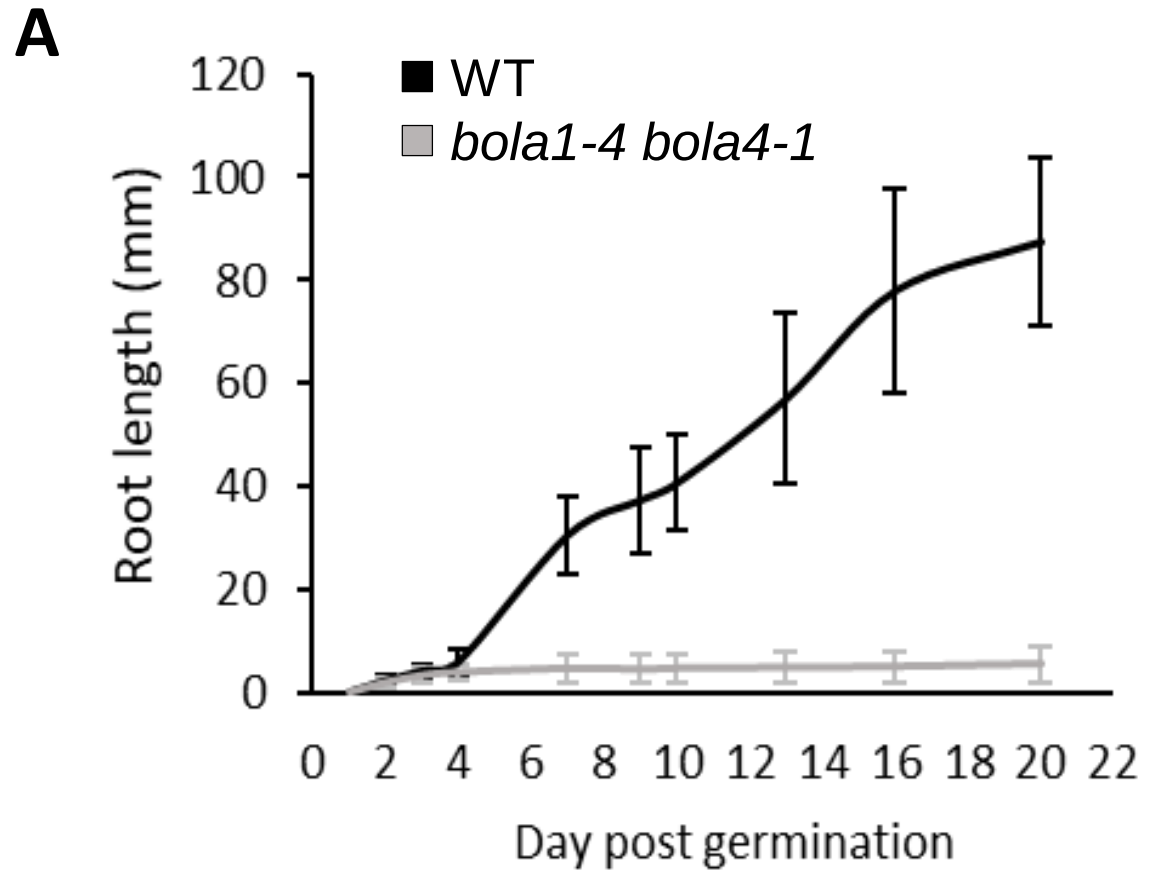

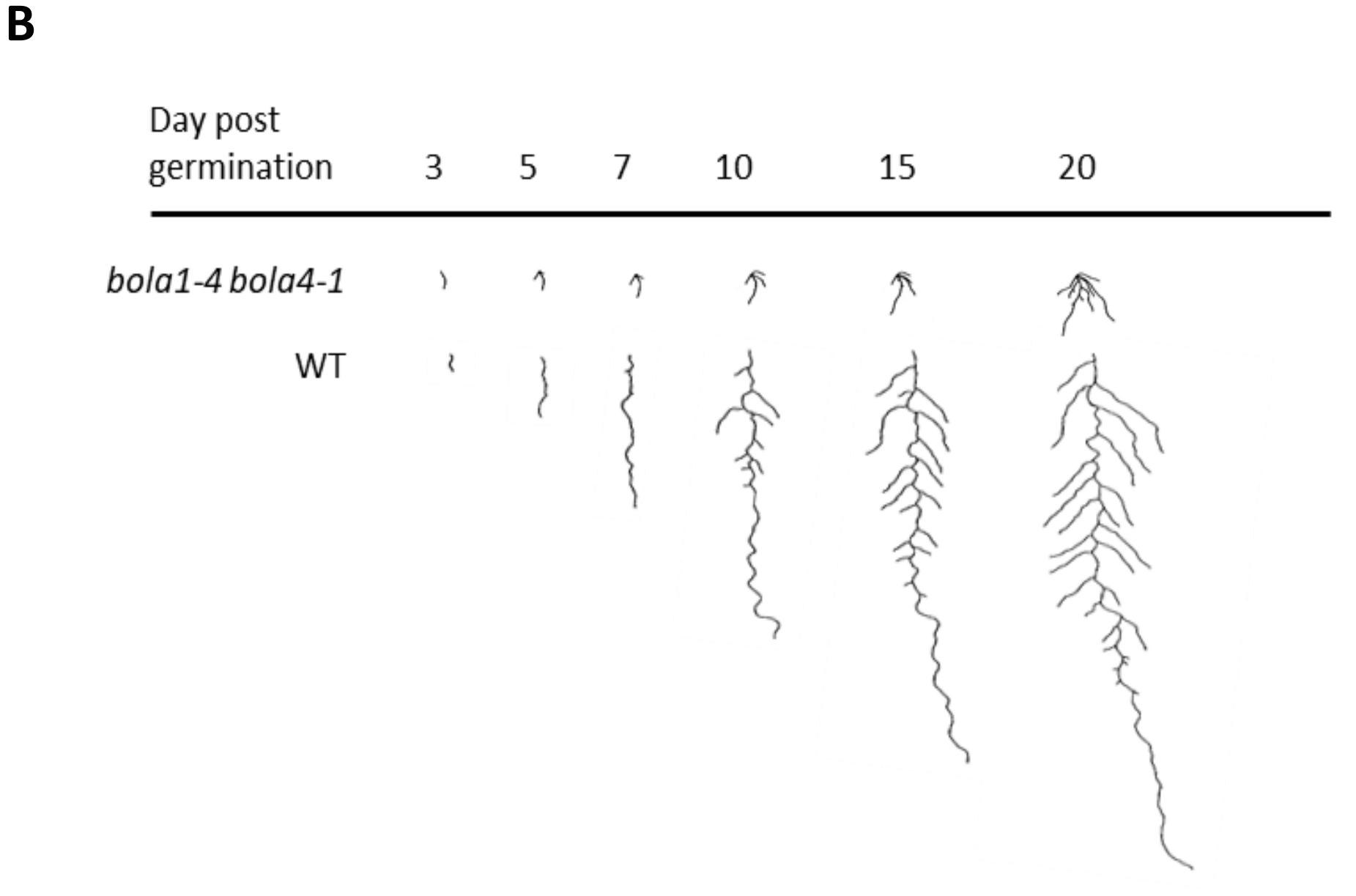

Figure S9. Comparative analysis of root growth over time between Arabidopsis wild-type plants and *bola* double mutants. **A)** Primary root length. **B) S**chematic representation of the wild type and *bola1-4 bola4-1* root systems.

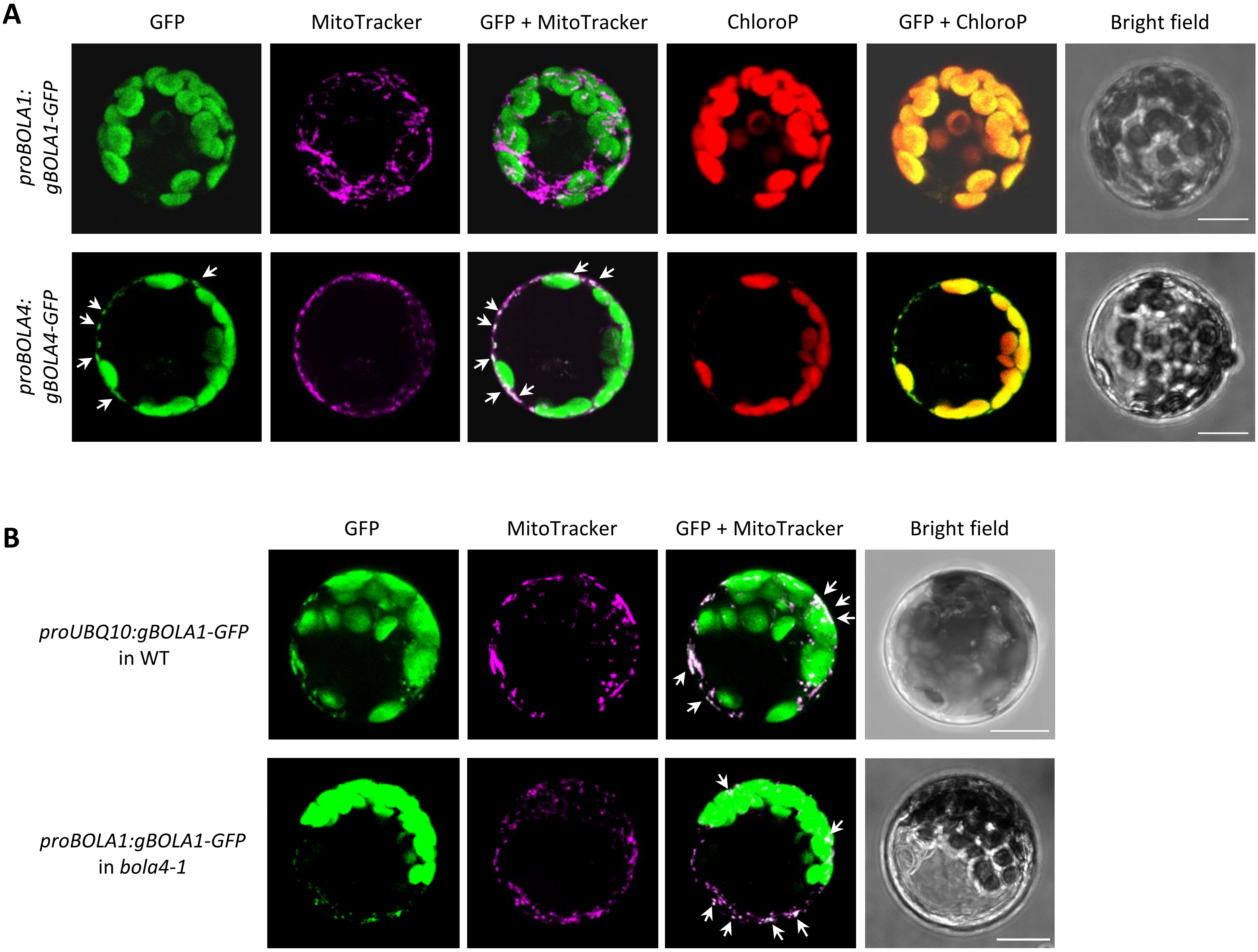

Figure S10. BOLA1, like BOLA4, is located in plastids and in mitochondria in Arabidopsis. **A)** Localization of BOLA1-GFP and BOLA4-GFP fusion proteins expressed from genomic copies of their gene under the control of their respective promoter (*proBOLA1:gBOLA1-GFP* and *proBOLA4:gBOLA4-GFP*) in wild type (WT) leaf protoplasts. **B)** Localization of the BOLA1-GFP fusion protein expressed either under the control of the *UBIQUITIN10* gene promoter (*proUBQ10:gBOLA1-GFP*) in WT leaf protoplasts, or under the control of its own promoter (*proBOLA1:gBOLA1-GFP*) in *bola4-1* leaf protoplasts. **(A,B)** Arrows indicate mitochondria where the fluorescence of the BOLA1-GFP and BOLA4-GFP fusion proteins overlaps with that of the MitoTracker™ CMROS Orange fluorescence (MitoTracker, coloured pink). Chloroplasts (ChloroP, coloured red) are labelled by chlorophyll auto-fluorescence. The overlay of their respective fluorescence with that of the GFP fusion proteins is shown in two separate panels. Image acquisition on the confocal microscope 24h after transfection is shown here with maximum Z-axis projection. Note that the fluorescence of both BOLA1-GFP and BOLA4-GFP proteins, which naturally accumulate in chloroplasts (Couturier *et al*., 2014), is saturated in this compartment, because the laser power was increased up to the standard detection of GFP fluorescence in mitochondria to record the presence of BOLA proteins in this specific compartment. All images are representative of five biological replicates, each based on approximately 20 cells displaying identical protein localization. Scale bar = 10 µm.

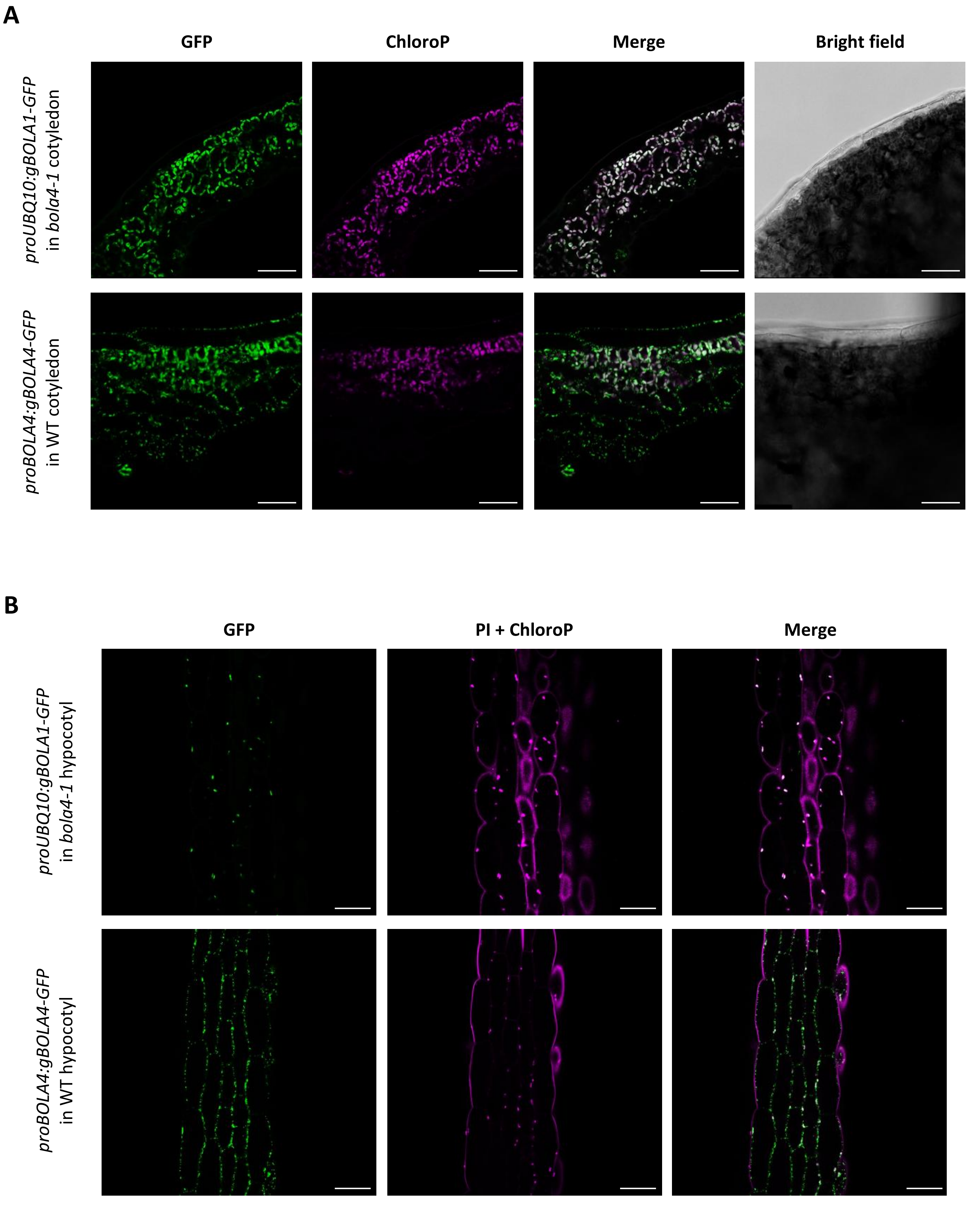

A
GFP
ChloroP
Merge
Bright field
proUBQ10:gBOLA1-GFP
in bola4-1 cotyledon
proBOLA4:gBOLA4-GFP
in WT cotyledon
B
GFP
PI + ChloroP
Merge
proUBQ10:gBOLA1-GFP
in bola4-1 hypocotyl
proBOLA4:gBOLA4-GFP
in WT hypocotyl

C

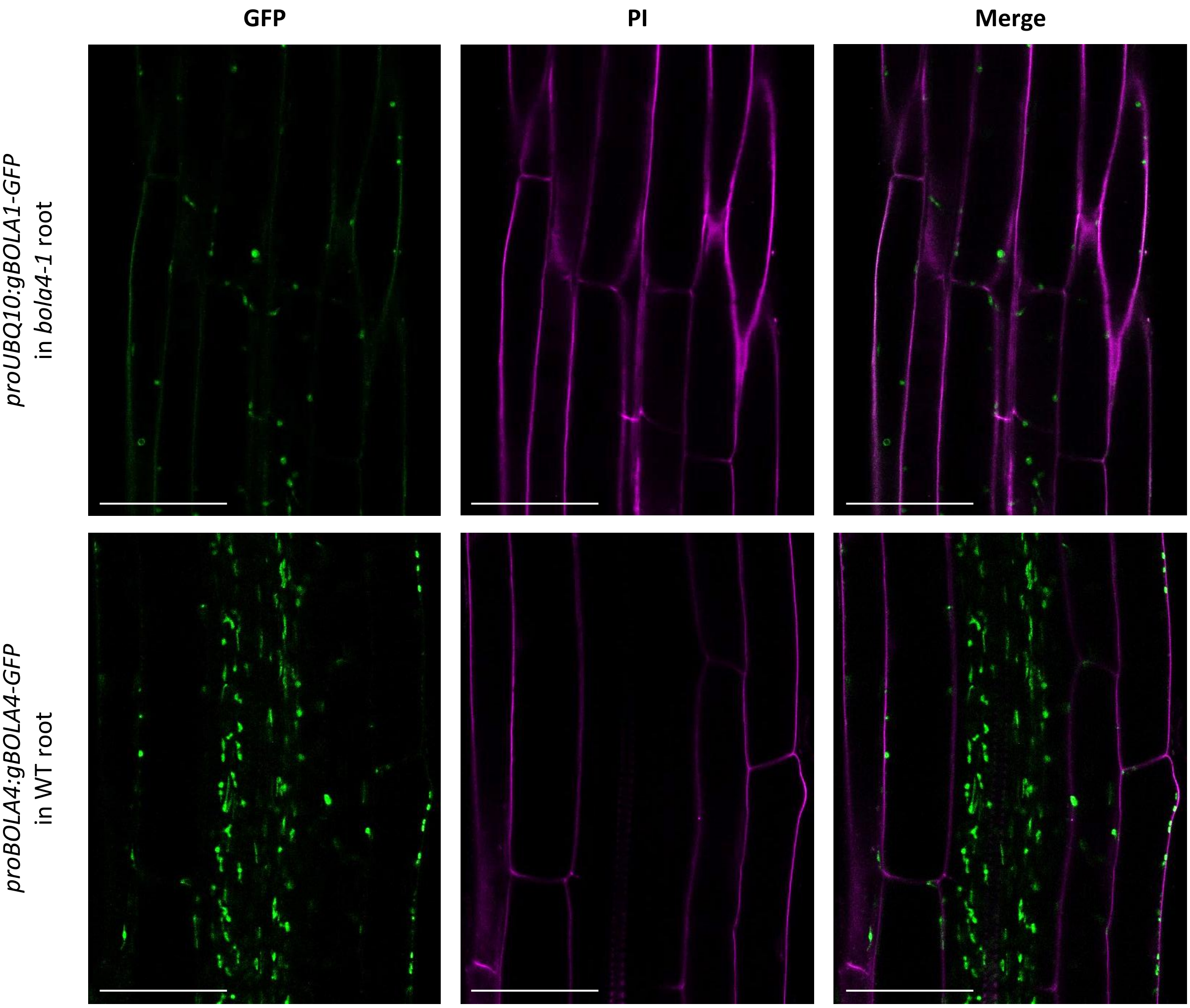
GFP
PI
Merge
proUBQ10:gBOLA1-GFP in bola4-1 root
proBOLA4:gBOLA4-GFP in WT root

D

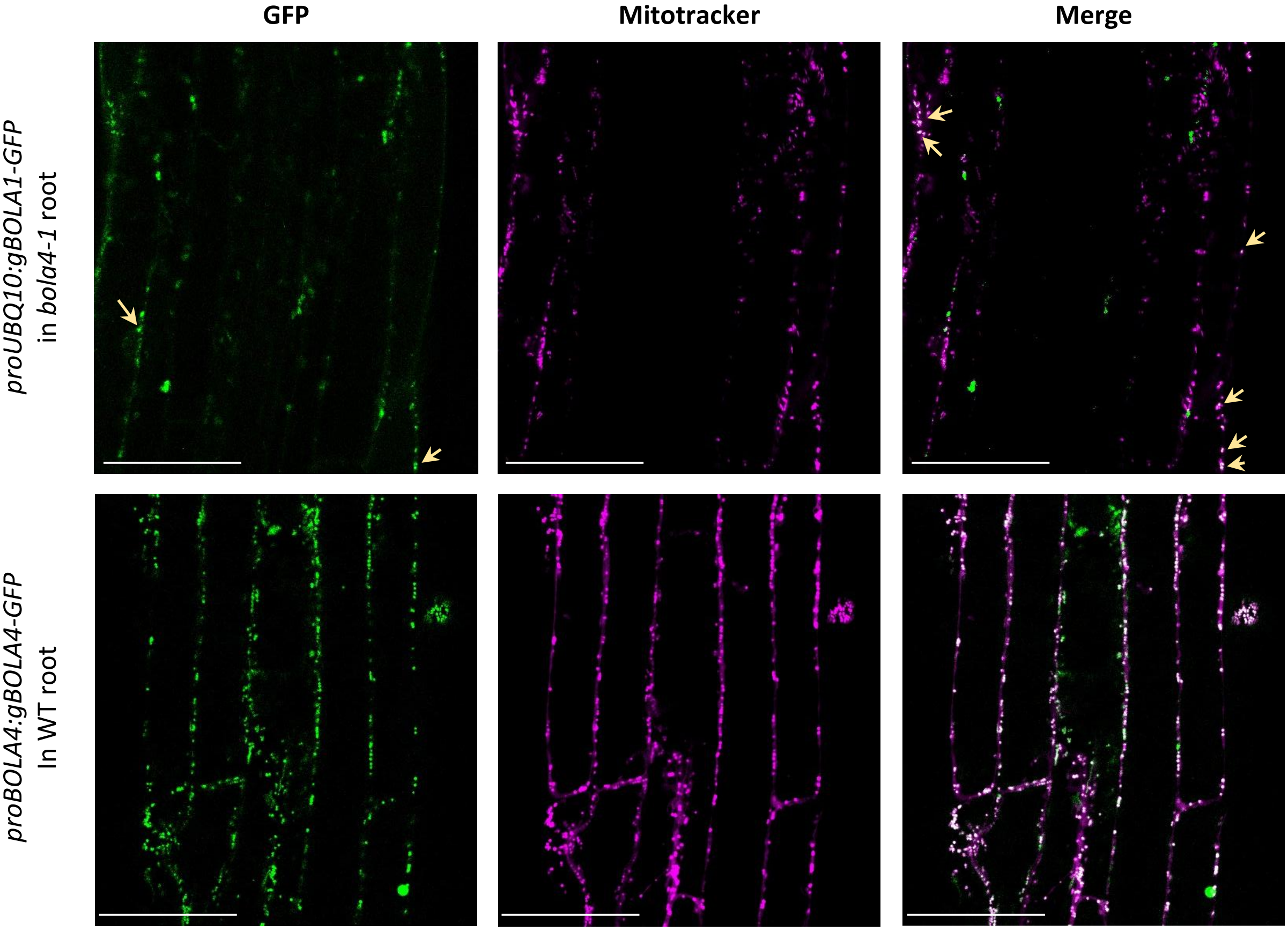
GFP
Mitotracker
Merge
proUBQ10:gBOLA1-GFP in bola4-1 root
proBOLA4:gBOLA4-GFP In WT root

Supplemental Figure S11. Subcellular localization of BOLA1 and BOLA4 in Arabidopsis tissues. Fluorescence patterns of BOLA1–GFP and BOLA4–GFP fusion proteins were analysed in 7-day-old *bola4-1* and WT cotyledons **(A)**, hypocotyls **(B)** and roots **(C,D)**. (**A-D)** BOLA1–GFP was expressed under the *UBQ10* promoter (upper panels), whereas BOLA4–GFP was expressed under its native promoter (*proBOLA4*; lower panels). Fluorescence of BOLA-GFP fusion proteins (GFP) was analysed in combination with autofluorescence of the chlorophyll (ChloroP) without **(A)** or with **(B)** propidium iodide (PI) in aerial tissues. The MitoTracker™ CMROS Orange (MitoT) stain did not penetrate into epidermal cells of aerial tissues, so the presence of BOLA in mitochondria was detected only through the GFP fluorescence in these tissues. Fluorescence of BOLA-GFP fusion proteins in roots was analysed in combination with PI **(C)** or MitoT **(D)**. Images are representative of at least 10 plants from two independent transgenic lines. Scale bars = 50 µm.

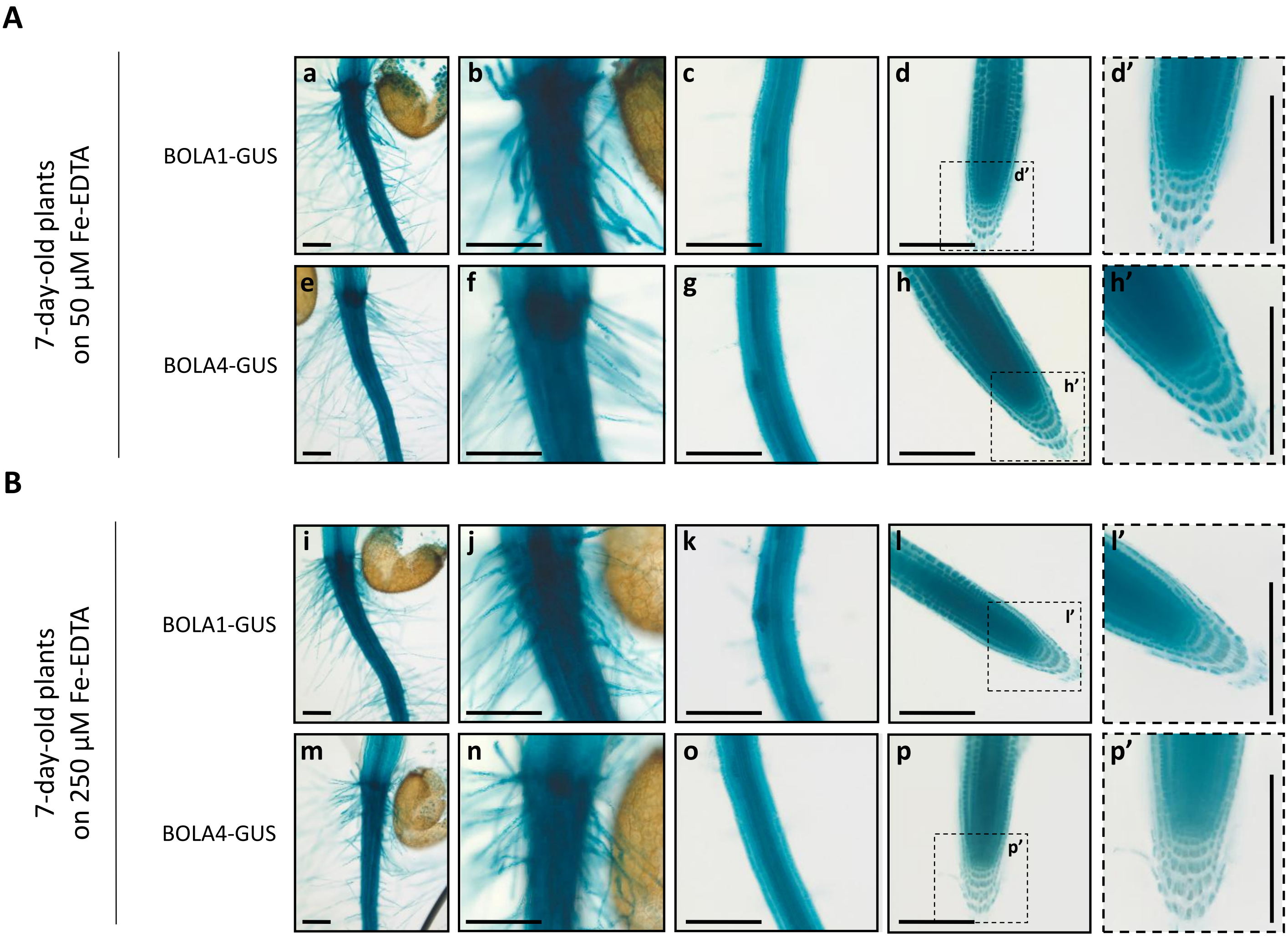

Figure S12. The *Arabidopsis thaliana* BOLA1 and BOLA4 proteins accumulate throughout the whole plant body, whatever the iron concentration in the medium. **(A,B)** GUS staining patterns of BOLA1-GUS and BOLA4-GUS fusion proteins expressed under their own promoter. Seedlings were 7 day-old on sterile ½MS media with **(A)** 50 µM [Fe-EDTA] (control condition) or with **(B)** 250 µM [Fe-EDTA] (iron excess condition). Images are representative of at least 20 plants from three independent transgenic lines per BOLA-GUS fusion protein analysed.

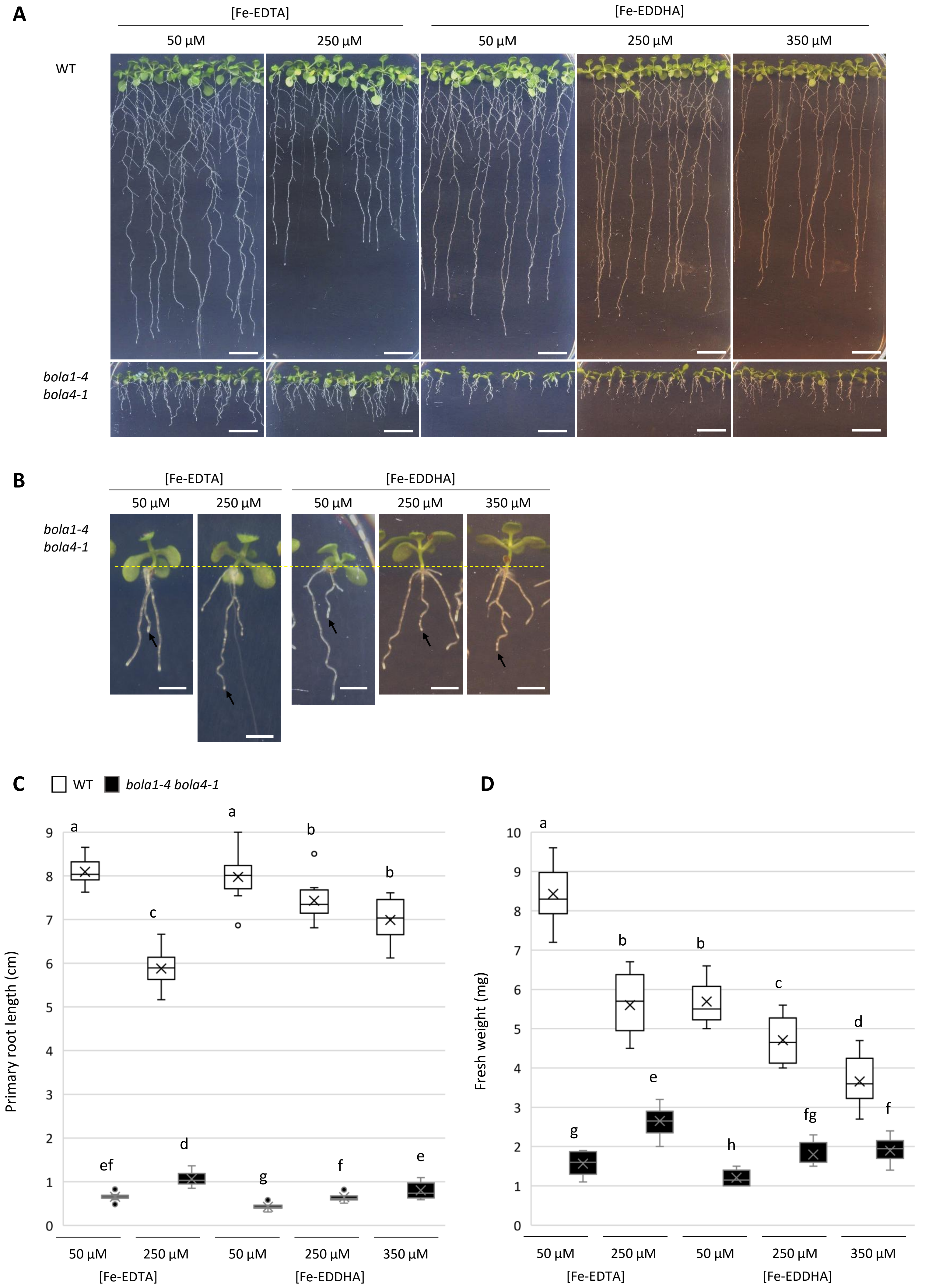

A
[Fe-EDTA]
[Fe-EDDHA]
50 µM
250 µM
50 µM
250 µM
350 µM
WT
bola1-4
bola4-1
B
[Fe-EDTA]
[Fe-EDDHA]
50 µM
250 µM
50 µM
250 µM
350 µM
bola1-4
bola4-1
C
WT
bola1-4 bola4-1
Primary root length (cm)
0
1
2
3
4
5
6
7
8
9
a
c
a
b
b
ef
d
g
f
e
50 µM
250 µM
50 µM
250 µM
350 µM
[Fe-EDTA]
[Fe-EDDHA]
D
Fresh weight (mg)
0
1
2
3
4
5
6
7
8
9
10
a
b
b
c
d
g
e
h
fg
f
50 µM
250 µM
50 µM
250 µM
350 µM
[Fe-EDTA]
[Fe-EDDHA]

Figure S13. Iron excess promotes *bola1-4 bola4-1* mutant growth independently of the Fe chelate used. **A)** Effect of iron excess on twelve-day-old WT and *bola1-4 bola4-1* plants grown vertically on ½ MS medium containing 1% sucrose, under sufficient (50 µM Fe-EDTA or Fe-EDDHA) or excess (250 µM Fe-EDTA or 250 or 350 µM Fe-EDDHA) iron conditions. Scale bars = 1 cm. **B)** Pictures of *bola1-4 bola4-1* plants growth on 50 µM, 250 µM or 350 µM Fe-EDDHA. The arrows indicate the primary root. Scale bars = 2 mm. **(C,D)** Fresh weight **(C)** and primary root length **(D)** of plants described in **(A)**. **(C,D)** Means within each condition with the same letter are not significantly different according to Kruskal-Wallis test with post-hoc analysis (Conover, 1999), $p < 0.05$, with Holm corrections ($n = 12$). Error bars show means ±SD.

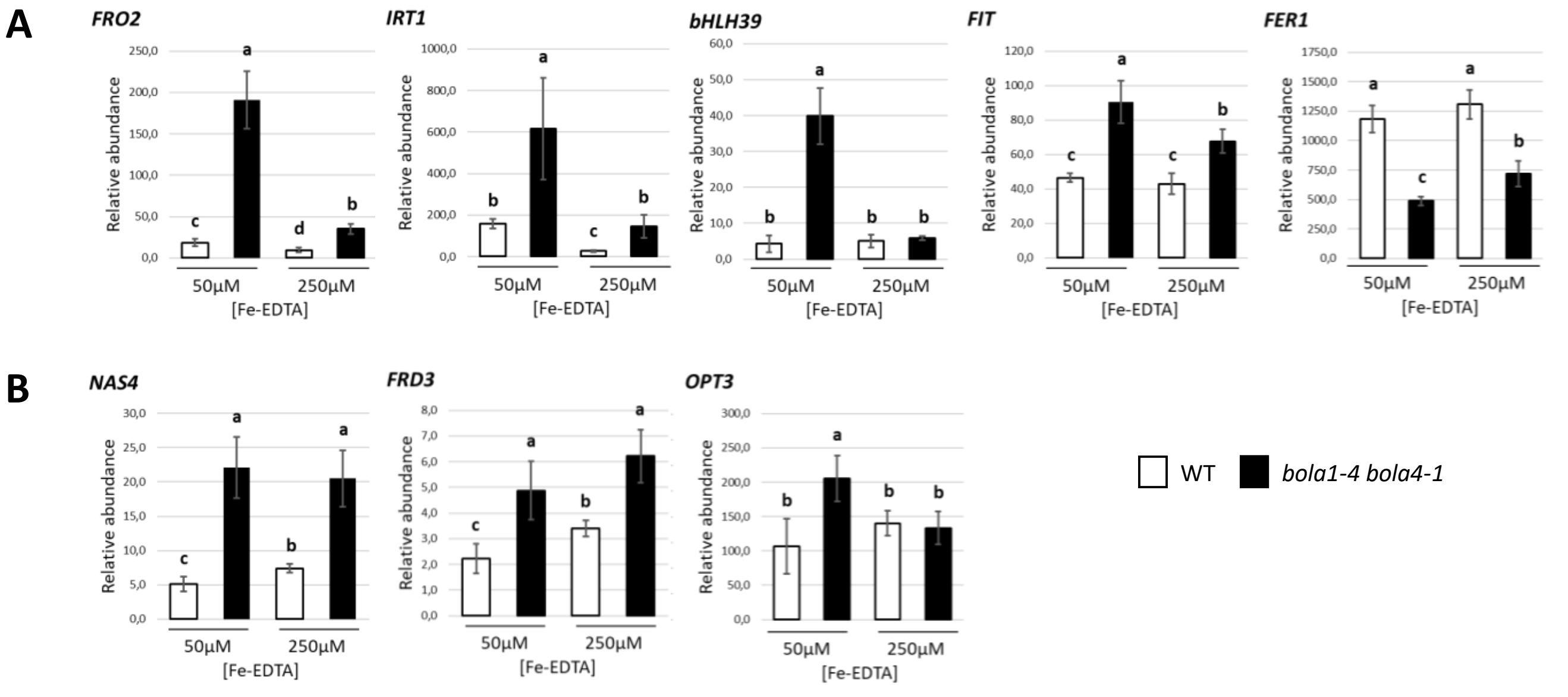

Supplemental Figure S14. The double *bola1-4 bola4-1* mutation induces an iron deficiency response in *Arabidopsis thaliana*. Expression of iron marker genes was analyzed in roots of twelve-day-old wild type (WT) and *bola1-4 bola4-1* mutant. **A)** Expression of genes involved in iron uptake (*FRO2*, *IRT1*), regulation of iron homeostasis *(bHLH39*, *FIT*) and iron storage (*FER1*). **B)** Expression of genes involved in nicotianamine transport (*NAS4*) and citrate efflux (*FRD3*) in the roots, and phloem-specific iron (*OPT3*) transport in the shoots. **(A,B)** Transcript abundance was assessed by RT-qPCR under control (50 µM Fe-EDTA) or high (250 µM Fe-EDTA) iron conditions, using the *PP2AA3* gene as control. Means within each condition with the same letter are not significantly different according to Kruskal-Wallis test with post-hoc analysis (Conover, 1999), $p < 0.01$, with Holm corrections. Error bars show means ± SD. All analyses were done in 4 independent biological repeats, with each experiment done in triplicates. A biological repeat required 15 WT and 75 *bola1-4 bola4-1* seedlings.

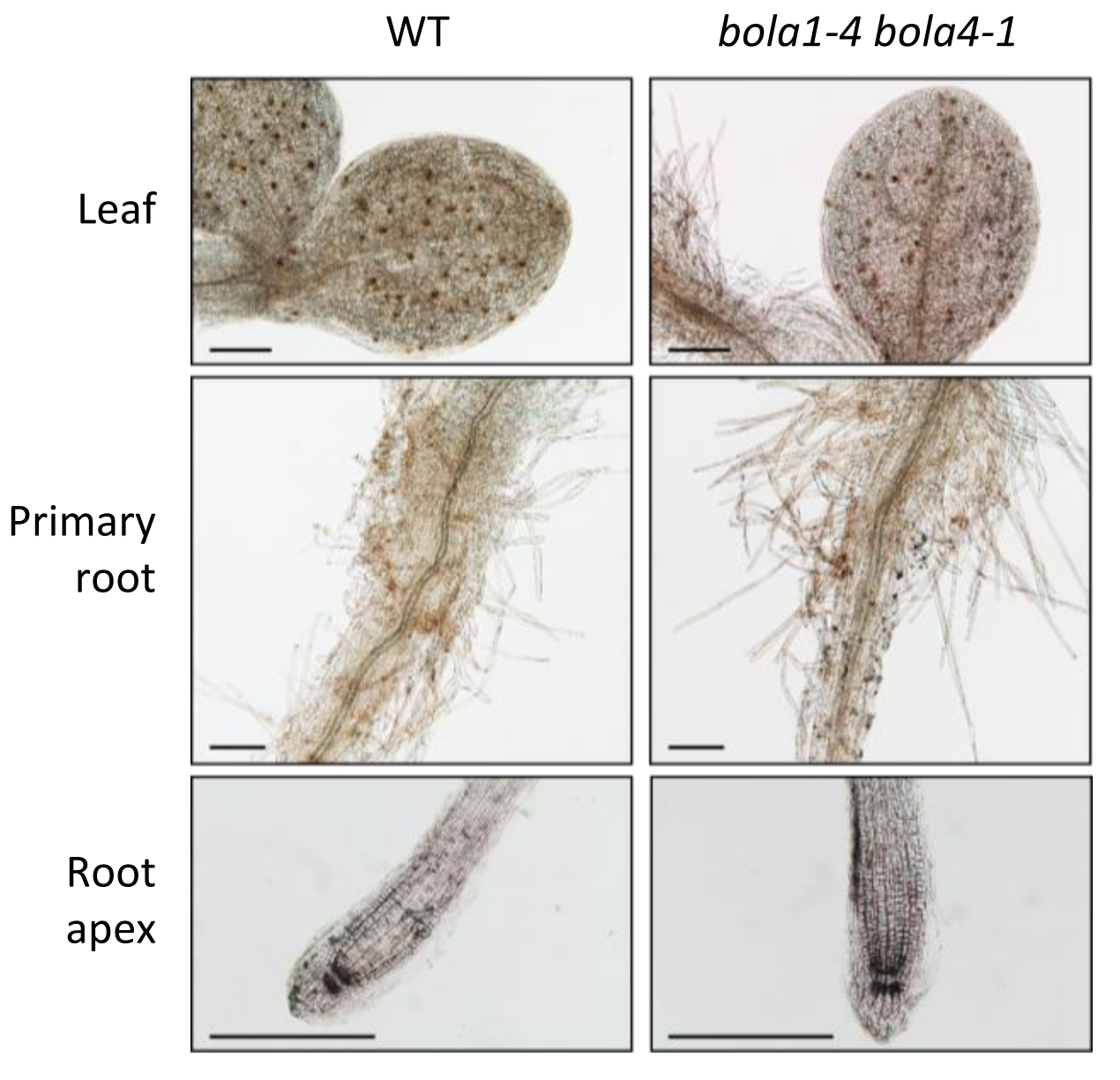

Supplemental Figure S15. Iron detection in three-day-old wild type and *bola1-4 bola4-1* double mutant. Plants were stained using the Perls/DAB method. Scale bars = 0.2 mm.

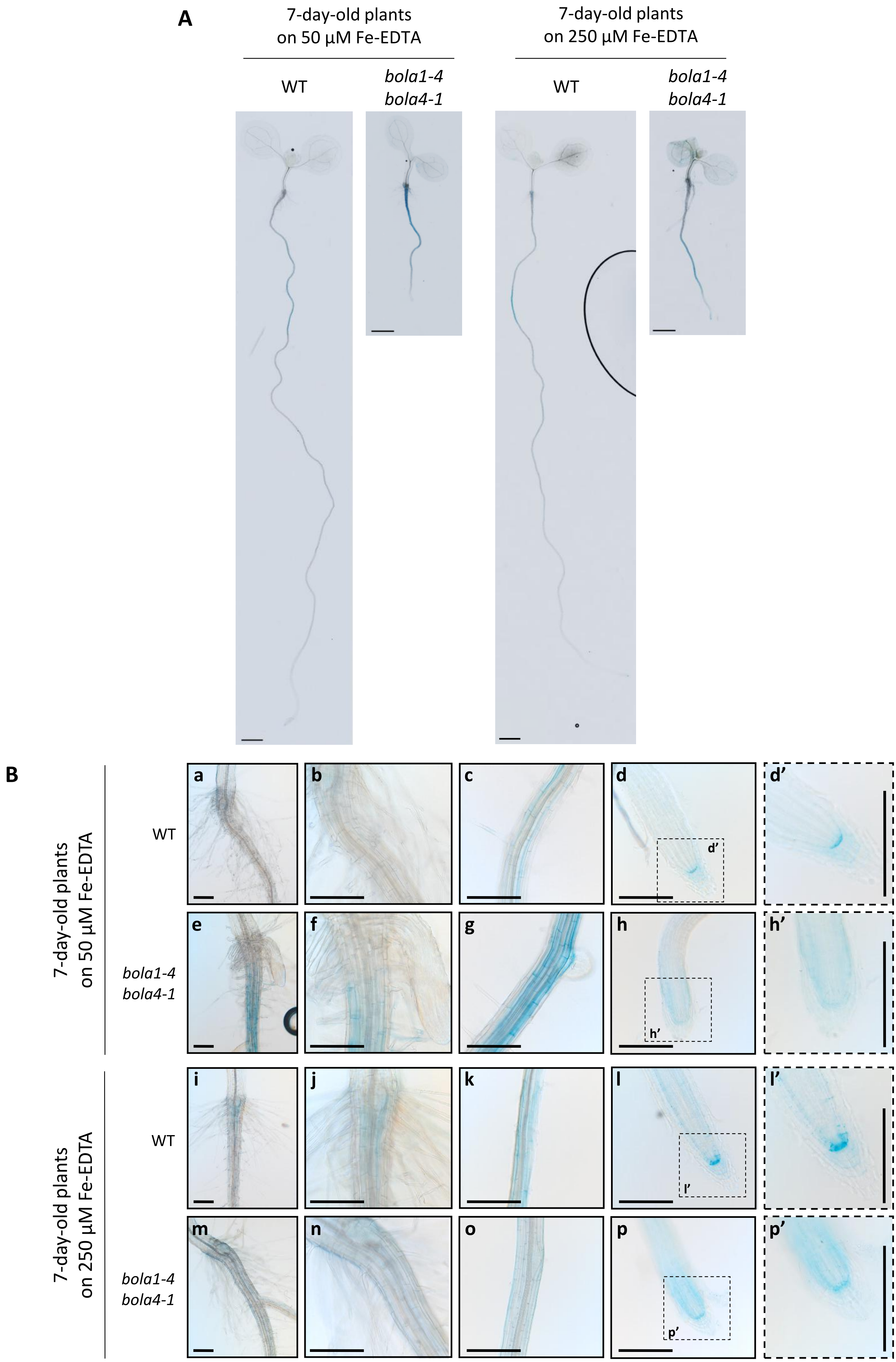
A
7-day-old plants
on 50 µM Fe-EDTA
7-day-old plants
on 250 µM Fe-EDTA
WT
bola1-4
bola4-1
WT
bola1-4
bola4-1
B
7-day-old plants
on 50 µM Fe-EDTA
WT
bola1-4
bola4-1
7-day-old plants
on 250 µM Fe-EDTA
WT
bola1-4
bola4-1
a
b
c
d
d'
e
f
g
h
h'
i
j
k
l
l'
m
n
o
p
p'

Supplemental Figure S16. The double *bola1-4 bola4-1* mutation alters iron partitioning in Arabidopsis roots. **A)** Whole-plant Perls staining showing Fe distribution in seven-day old WT and *bola1-4 bola4-1* double mutant seedlings grown under sufficient (50 µM Fe-EDTA) or iron excess (250 µM Fe-EDTA) conditions. Right panels (250 µM Fe) show that Fe excess partially rescues Fe distribution along the mutant root, resembling that of the WT root. Scale bars = 1 cm. **B)** Focus on Perls stainings of seven-day-old wild-type **(a-d, i-l)** and *bola1-4 bola4-1* **(e-h, m-p)** primary roots under sufficient (**a-h**) or excess (**i-p**) Fe-EDTA. **(a,e,i,m)** Views from the bottom of the hypocotyl to the upper part of the primary root, with a focus on the collar area **(b,f,j,n)**. **(c,g,k,o)** Maturation zones. **(d,h,l,p)** Root tips, with insets (**l',p'**) highlighting the meristematic and elongations zones. Scale bars = 0.2 mm.

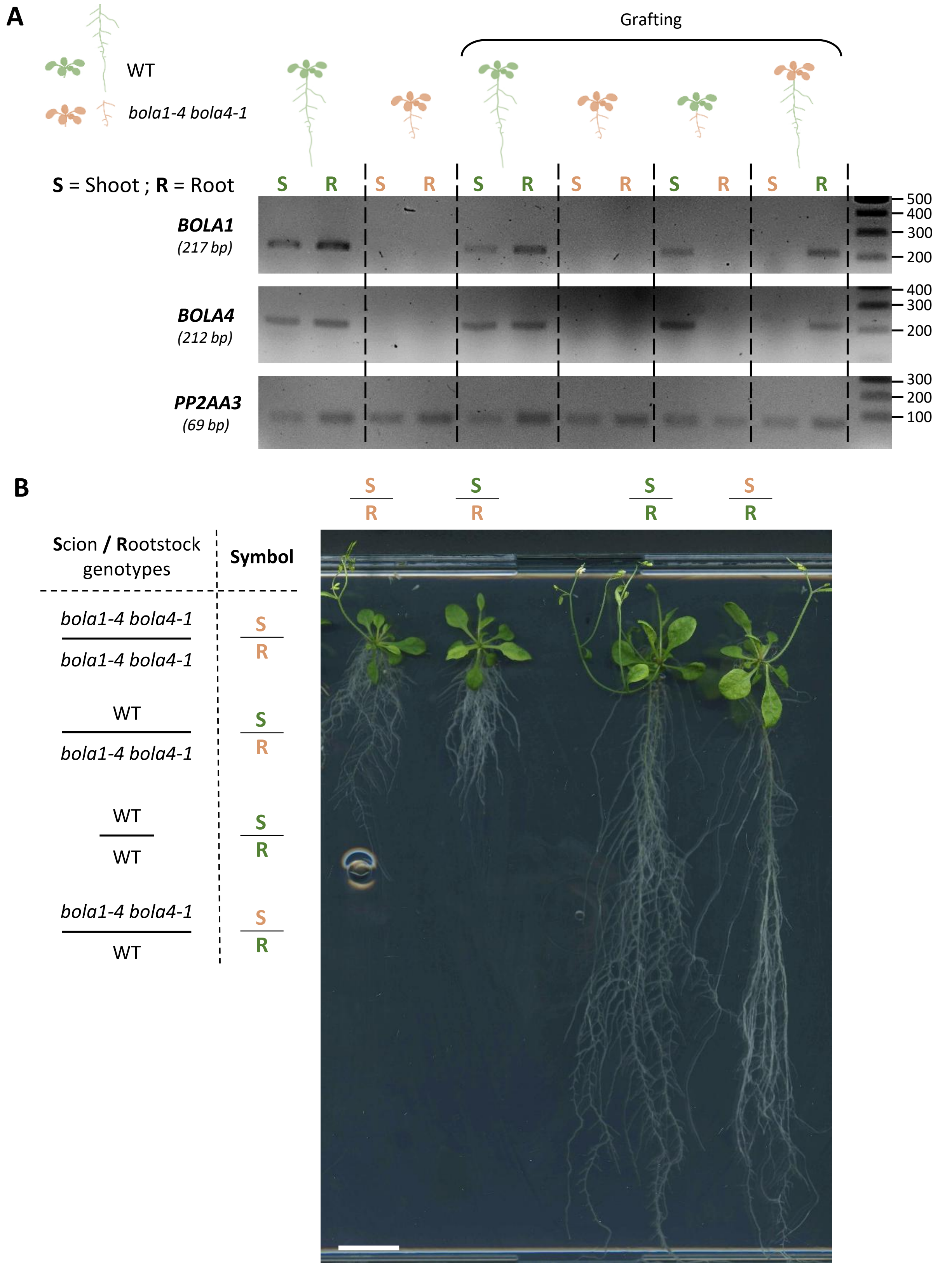

Figure S17. Reciprocal grafting experiments between wild-type plants and the *bola1-4 bola4-1* double mutant. **A)** *BOLA1* and *BOLA4* gene expression in grafted plants shown in **(B)** was analyzed by RT-PCR on cDNAs obtained from shoot (S) and root (R) transcripts of the different genotypes. Oligonucleotide pairs were designed to detect a 217 bp cDNA fragment of *BOLA1*, a 212 bp cDNA fragment of *BOLA4*, and a 69 bp cDNA fragment of *PP2AA3* as control (Supplemental Table S2). **B)** Picture of the grafted plants five weeks post-grafting. Shoot scion (S) and rootstock (R) genotypes (green letters for WT, orange letters for *bola1-4 bola4-1*) are indicated above and below the lines, respectively. WT/WT and *bola1-4 bola4-1/bola1-4 bola4-1* grafted plants were used as positive and negative controls, respectively. Scale bar = 1 cm.

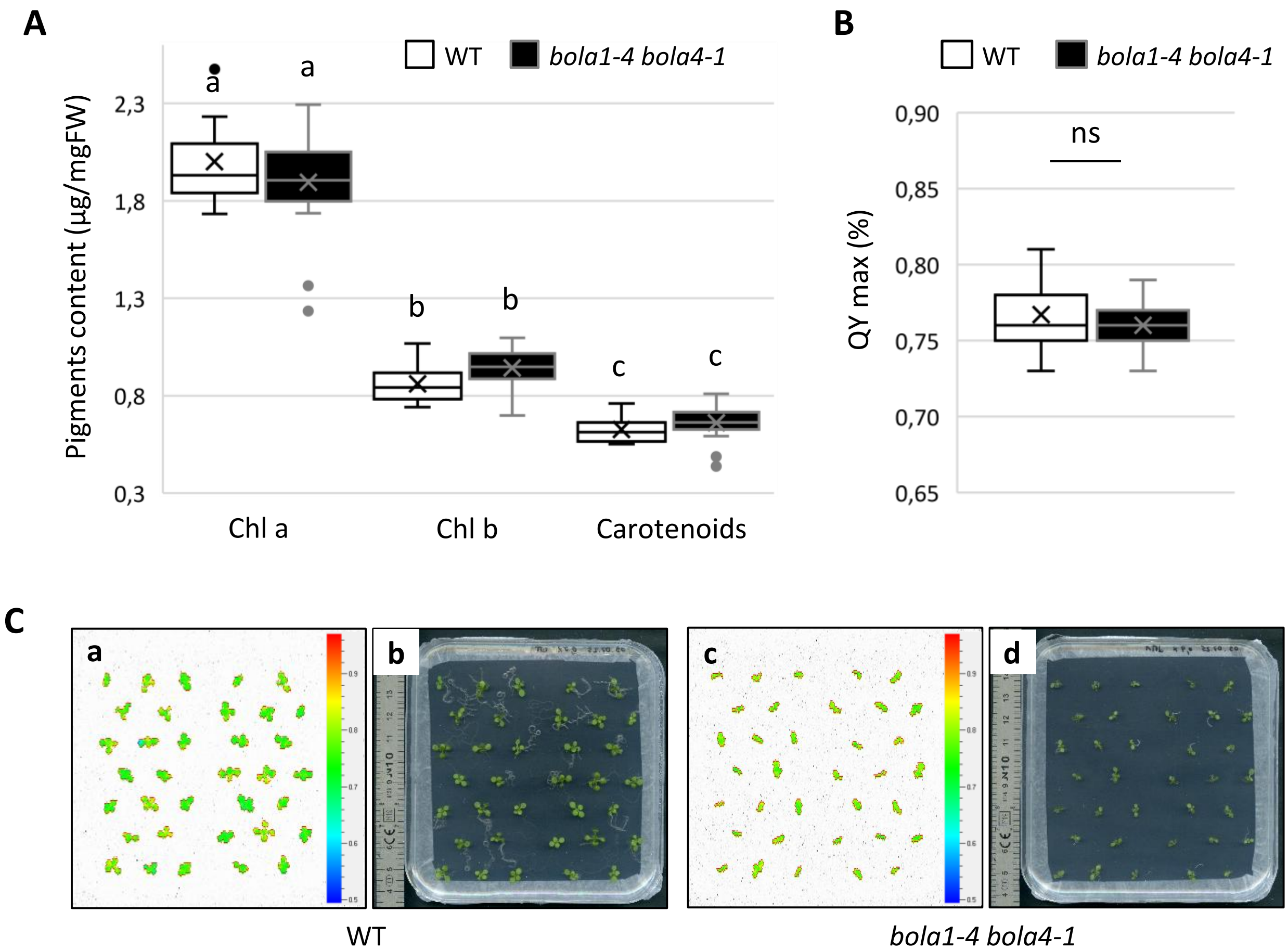

Figure S18. Photosynthetic activity in the *bola1-4 bola4-1* double mutant. **A)** Pigments content measured wild type and *bola1-4 bola4-1* leaves. Means with the same letter are not significantly different according to Kruskal-Wallis test with post-hoc analysis (Conover, 1999), $p < 0.05$, with Bufferoni corrections. Error bars show means ± SD. n = 8 independent biological replicates, each requiring 15 WT and 22 *bola1-4 bola4-1* areal parts. **B)** Photosynthetic potential of the *bola1-4 bola4-1* mutant compared to the wild-type plant. The maximum quantum yield (QY max) corresponds to the maximum photon absorption capacity by Photosystem II. Means are not significantly different according to a t-test ($p <$ 0,05). Error bars show means ± SD. n = 2 independent biological replicates, each performed on 35 seedlings per genotype. **C)** False-color images generated by the Fluorcam7 Software (Photon System Instrument) to obtain QY max values under actinic light **(a,c)**, and plants used for QY max measurement under white light **(b,d)**. **(A,B,C)** All experiments were done using 10-day-old seedlings grown horizontally on ½ MS media with 1% sucrose.

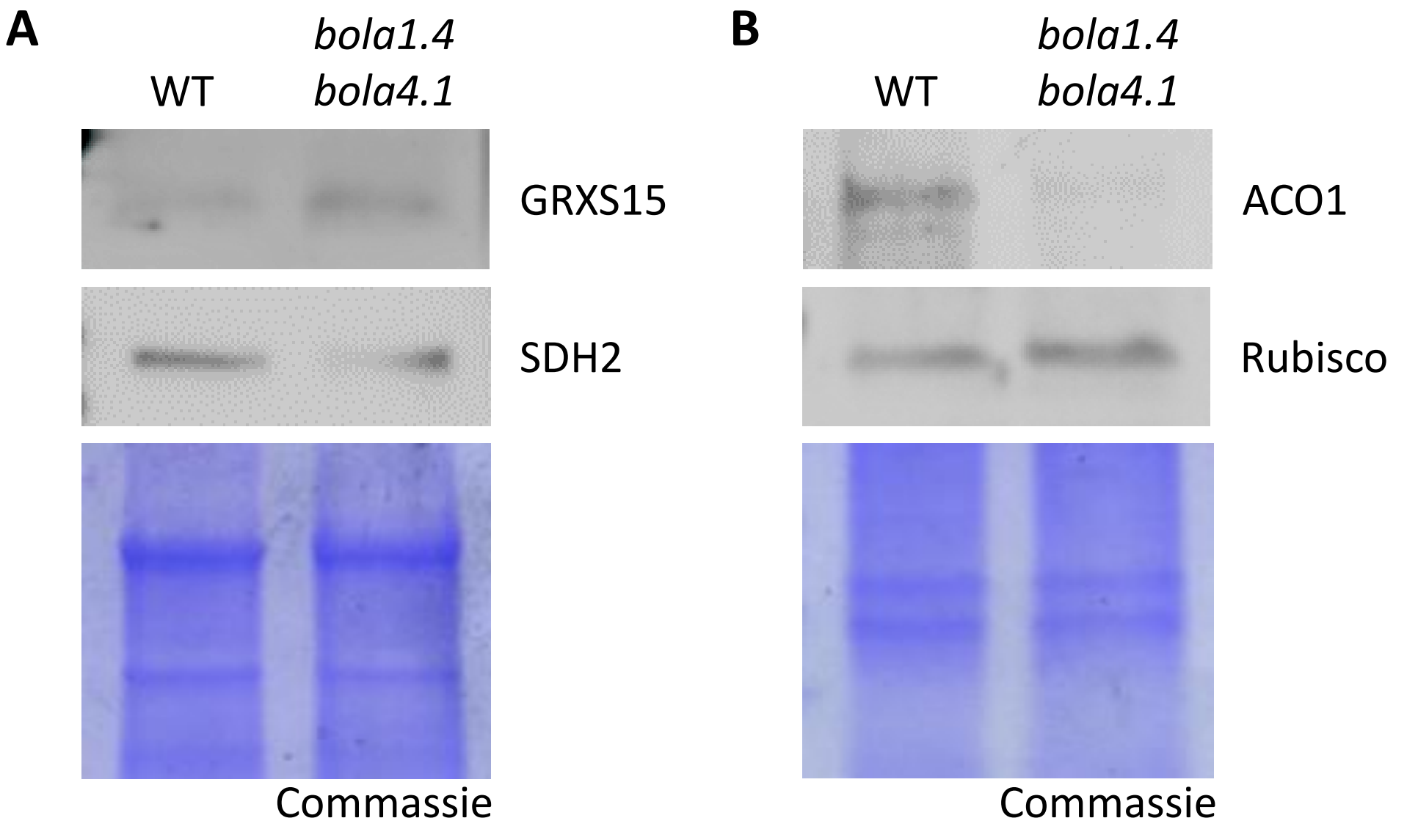

Figure S19. Immunoblot analysis of the abundance of the mitochondrial GRXS15 and SDH2, and the cytosolic ACO1 in the WT and *bola1-4 bola4-1* plants. All proteins analysed were obtained from 15-day-old plants. **A)** Mitochondrial proteins were obtained from roots and extracted according to Havird et al. (2019). **B)** Total proteins were extracted from rosette leaves using a RIPA lysis buffer. **(A,B)** Proteins (40 µg in 1X Laemmli buffer) were separated on 15% (GRXS15) or 10% (SDH2, ACO1, Rubisco) SDS-PAGE before transferring to PVDF membranes and revealed by cheluminescence using Goat anti-Rabbit IgG (H&L), HRP conjugated antibodies (Agrisera). The polyclonal antibody against Arabidopsis GRXS15 was obtained from N. Rouhier (IAM Nancy, France). Antibodies against ACO1, Rubisco and SDH2-1/2-2 were obtained from Agrisera and used according to the furnisher's recommendation. A PageRuler Prestained Protein Ladder (ThermoFisher Scientific) was used as the molecular mass marker in gels. The Precision Plus Protein™ WesternC™ Blotting Standards and StrepTactin-HRP Conjugate (BioRad) were used to reveal protein markers on membranes post-transfer. Commassie staining of the proteins in gels are shown as loading controls.
